# Supplementary material for: Benchmarking informatics workflows for data-independent acquisition single-cell proteomics
Source: Nat Commun. 2025 Nov 21;16:10276. doi: 10.1038/s41467-025-65174-4 (PMC12639053; doi:10.1038/s41467-025-65174-4)
Supplement: Supplementary file 1 — Supplementary Information [file 41467_2025_65174_MOESM1_ESM.pdf]

Supplementary Information

Benchmarking informatics workflows for data-independent acquisition single-cell proteomics

Jianwei Wang et al.

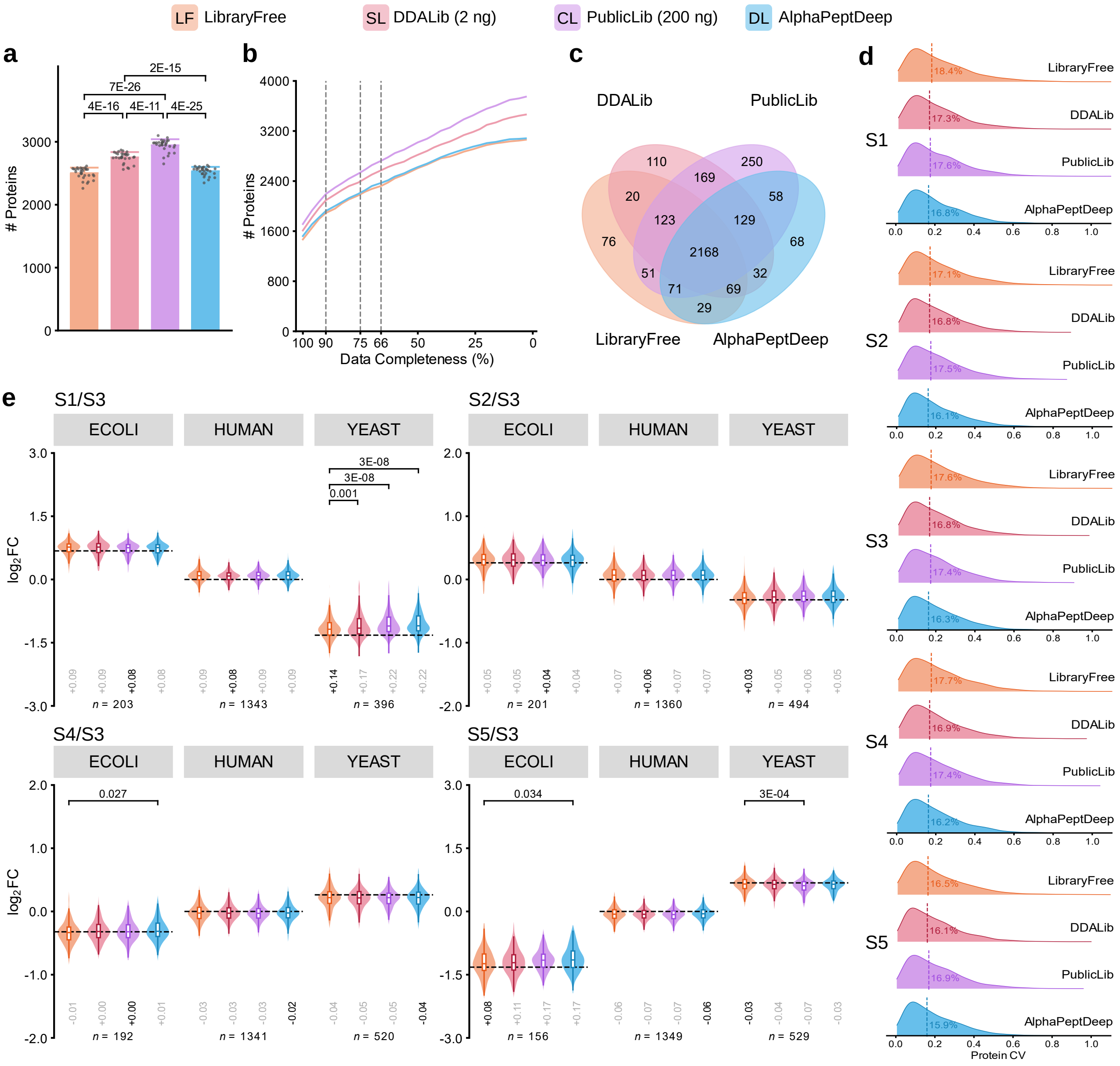

Supplementary Figure 1. Performance comparison of different searching strategies using DIA-NN at the protein level.

a Numbers of quantified proteins per run. The bars indicate the mean values and the error bars indicate the standard deviations. Significant differences (t-test p-value < 0.05, two-sided, no adjustments) are indicated. **b** Numbers of proteins quantified in at least specified percentages (data completeness) of runs. **c** Overlap of the proteins quantified in at least 50% runs. **d** Distribution of the coefficient of variation (CV). CV values were calculated only for proteins quantified in at least 3 runs per sample. The median values are indicated. **e** Measured fold change (FC) values of protein quantities using sample S3 as reference. FC values were calculated only for proteins quantified in at least 3 runs for each sample of the comparison. Numbers (*n*) of proteins are indicated for each species. The boxes mark the first and third quantile and the lines inside the boxes mark the median; the whiskers extend from the box to the farthest point lying within 1.5 times the inter-quartile range; outliers are not shown. The theoretical ratios are highlighted as dashed lines. Differences between the measured median FC values and theoretical values are indicated, among which the smallest ones are darkened. Significant differences (t-test p-value < 0.05 and Cohen's |*d*| > 0.2, two-sided, no adjustments) are indicated. Source Data are provided as a Source Data file.

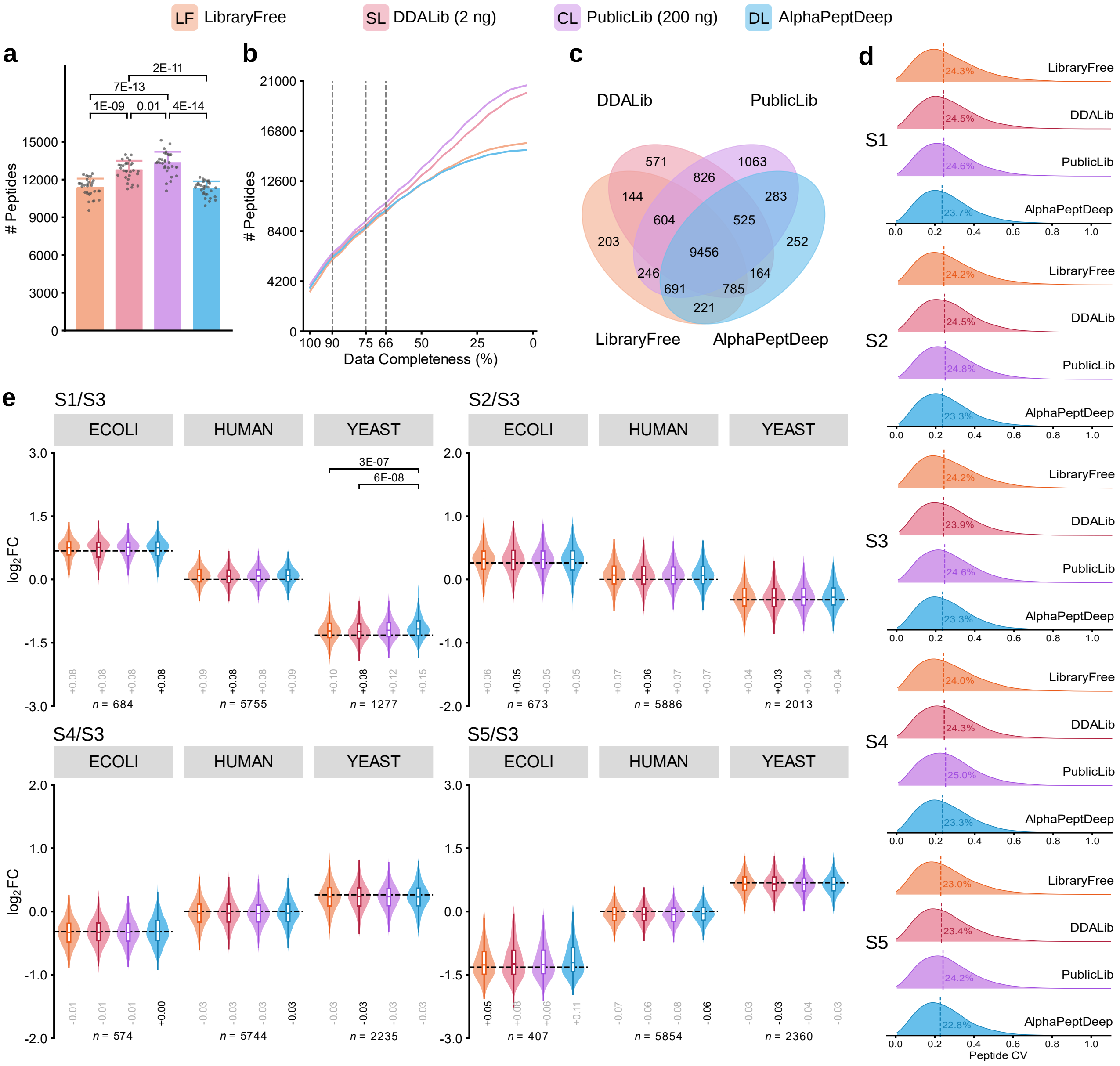

Supplementary Figure 2. Performance comparison of different searching strategies using DIA-NN at the peptide level.

a Numbers of quantified peptides per run. The bars indicate the mean values and the error bars indicate the standard deviations. Significant differences (t-test p-value < 0.05, two-sided, no adjustments) are indicated. **b** Numbers of peptides quantified in at least specified percentages (data completeness) of runs. **c** Overlap of the peptides quantified in at least 50% runs. **d** Distribution of the coefficient of variation (CV). CV values were calculated only for peptides quantified in at least 3 runs per sample. The median values are indicated. **e** Measured fold change (FC) values of peptide quantities using sample S3 as reference. FC values were calculated only for peptides quantified in at least 3 runs for each sample of the comparison. Numbers (n) of peptides are indicated for each species. The boxes mark the first and third quantile and the lines inside the boxes mark the median; the whiskers extend from the box to the farthest point lying within 1.5 times the inter-quartile range; outliers are not shown. The theoretical ratios are highlighted as dashed lines. Differences between the measured median FC values and theoretical values are indicated, among which the smallest ones are darkened. Significant differences (t-test p-value < 0.05 and Cohen's $|d|$ > 0.2, two-sided, no adjustments) are indicated. Source data are provided as a Source Data file.

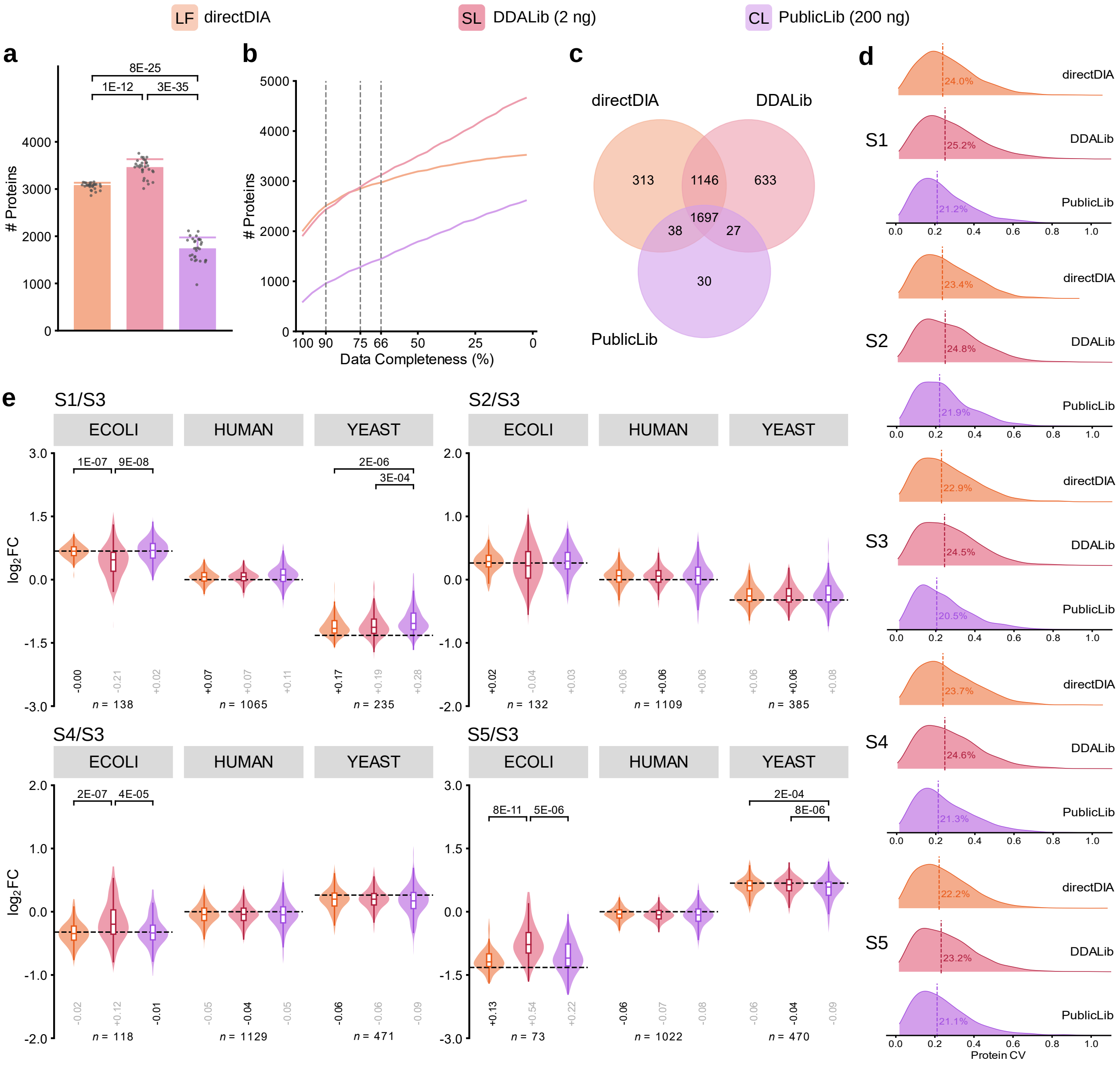

Supplementary Figure 3. Performance comparison of different searching strategies using Spectronaut at the protein level.

a Numbers of quantified proteins per run. The bars indicate the mean values and the error bars indicate the standard deviations. Significant differences (t-test p-value < 0.05, two-sided, no adjustments) are indicated. **b** Numbers of proteins quantified in at least specified percentages (data completeness) of runs. **c** Overlap of the proteins quantified in at least 50% runs. **d** Distribution of the coefficient of variation (CV). CV values were calculated only for proteins quantified in at least 3 runs per sample. The median values are indicated. **e** Measured fold change (FC) values of protein quantities using sample S3 as reference. FC values were calculated only for proteins quantified in at least 3 runs for each sample of the comparison. Numbers (*n*) of proteins are indicated for each species. The boxes mark the first and third quantile and the lines inside the boxes mark the median; the whiskers extend from the box to the farthest point lying within 1.5 times the inter-quartile range; outliers are not shown. The theoretical ratios are highlighted as dashed lines. Differences between the measured median FC values and theoretical values are indicated, among which the smallest ones are darkened. Significant differences (t-test p-value < 0.05 and Cohen's |*d*| > 0.2, two-sided, no adjustments) are indicated. Source data are provided as a Source Data file.

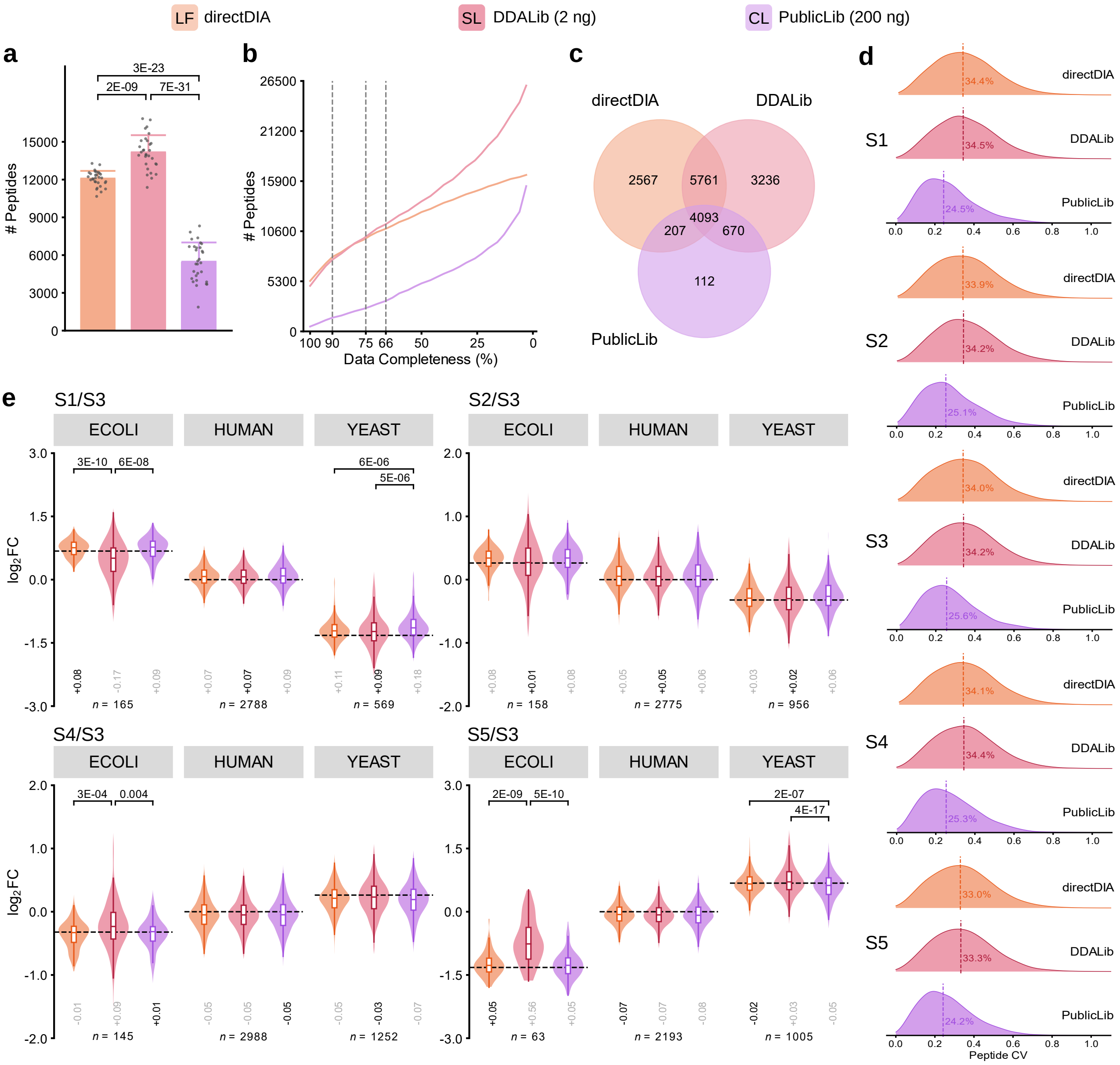

Supplementary Figure 4. Performance comparison of different searching strategies using Spectronaut at the peptide level.

a Numbers of quantified peptides per run. The bars indicate the mean values and the error bars indicate the standard deviations. Significant differences (t-test p-value < 0.05, two-sided, no adjustments) are indicated. **b** Numbers of peptides quantified in at least specified percentages (data completeness) of runs. **c** Overlap of the peptides quantified in at least 50% runs. **d** Distribution of the coefficient of variation (CV). CV values were calculated only for peptides quantified in at least 3 runs per sample. The median values are indicated. **e** Measured fold change (FC) values of peptide quantities using sample S3 as reference. FC values were calculated only for peptides quantified in at least 3 runs for each sample of the comparison. Numbers (*n*) of peptides are indicated for each species. The boxes mark the first and third quantile and the lines inside the boxes mark the median; the whiskers extend from the box to the farthest point lying within 1.5 times the inter-quartile range; outliers are not shown. The theoretical ratios are highlighted as dashed lines. Differences between the measured median FC values and theoretical values are indicated, among which the smallest ones are darkened. Significant differences (t-test p-value < 0.05 and Cohen's |*d*| > 0.2, two-sided, no adjustments) are indicated. Source data are provided as a Source Data file.

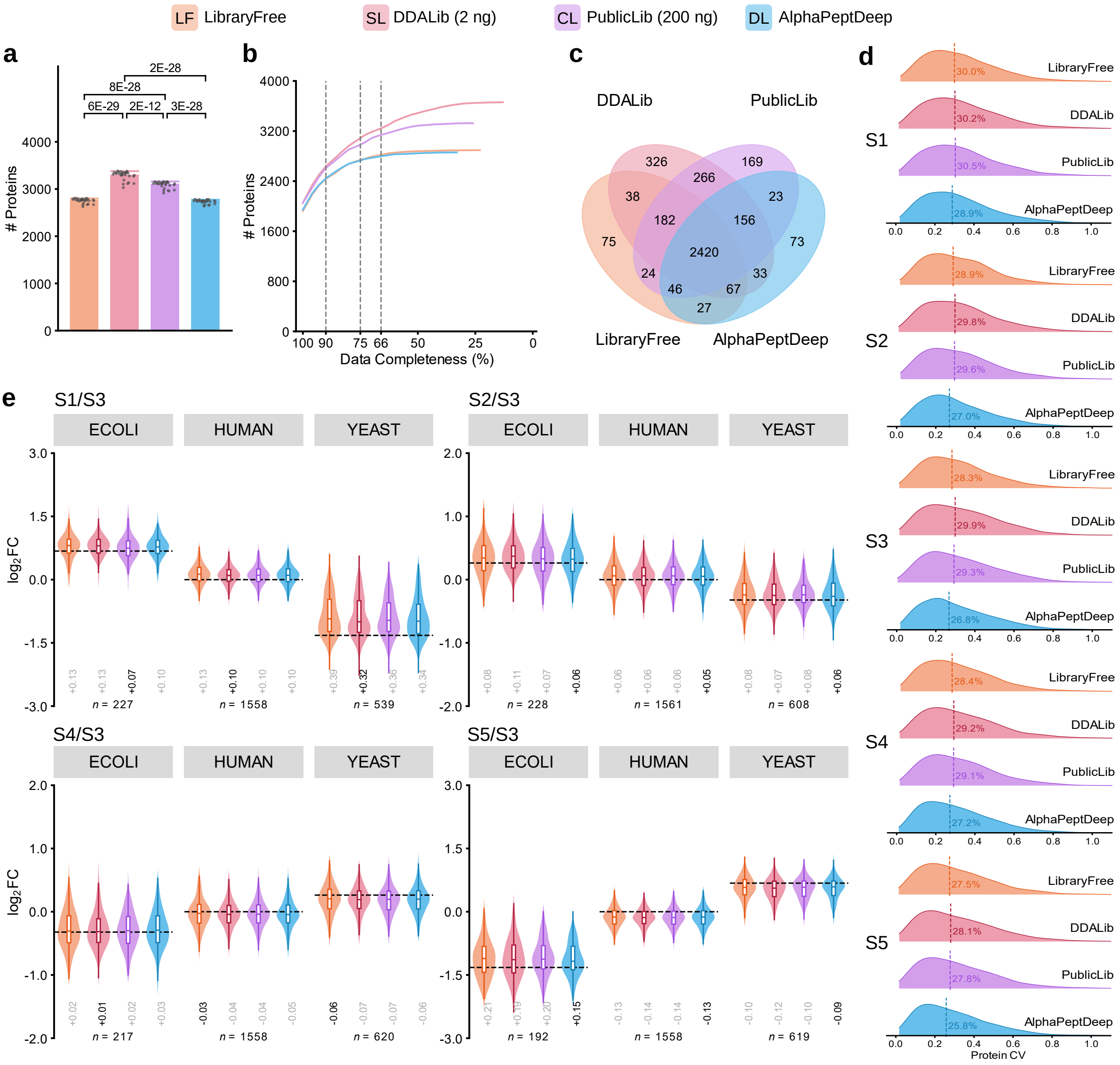

Supplementary Figure 5. Performance comparison of different searching strategies using PEAKS at the protein level.

a Numbers of quantified proteins per run. The bars indicate the mean values and the error bars indicate the standard deviations. Significant differences (t-test p-value < 0.05, two-sided, no adjustments) are indicated. **b** Numbers of proteins quantified in at least specified percentages (data completeness) of runs. **c** Overlap of the proteins quantified in at least 50% runs. **d** Distribution of the coefficient of variation (CV). CV values were calculated only for proteins quantified in at least 3 runs per sample. The median values are indicated. **e** Measured fold change (FC) values of protein quantities using sample S3 as reference. FC values were calculated only for proteins quantified in at least 3 runs for each sample of the comparison. Numbers (*n*) of proteins are indicated for each species. The boxes mark the first and third quantile and the lines inside the boxes mark the median; the whiskers extend from the box to the farthest point lying within 1.5 times the inter-quartile range; outliers are not shown. The theoretical ratios are highlighted as dashed lines. Differences between the measured median FC values and theoretical values are indicated, among which the smallest ones are darkened. Significant differences (t-test p-value < 0.05 and Cohen's |*d*| > 0.2, two-sided, no adjustments) are indicated. Source data are provided as a Source Data file.

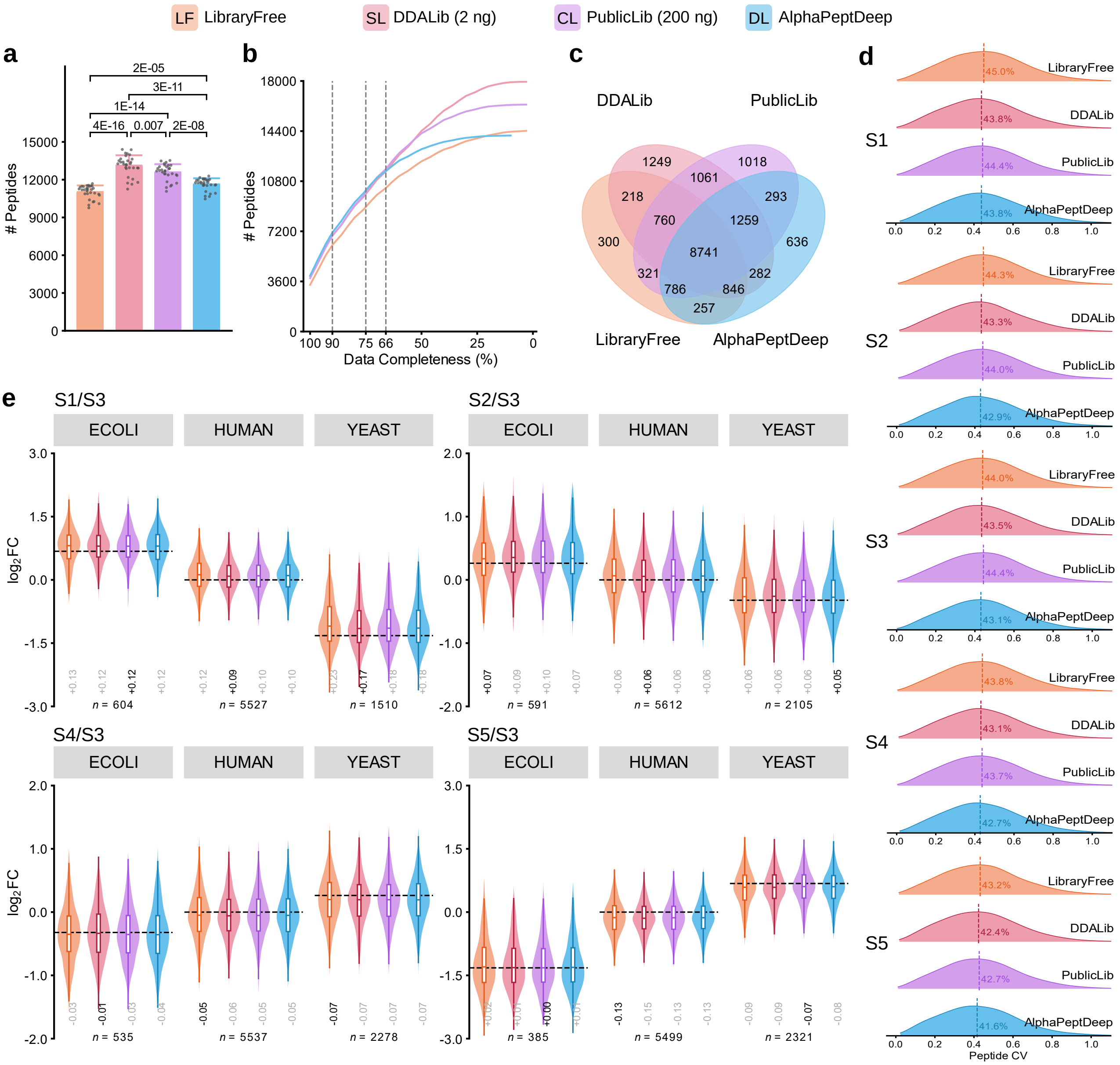

Supplementary Figure 6. Performance comparison of different searching strategies using PEAKS at the peptide level.

a Numbers of quantified peptides per run. The bars indicate the mean values and the error bars indicate the standard deviations. Significant differences (t-test p-value < 0.05, two-sided, no adjustments) are indicated. **b** Numbers of peptides quantified in at least specified percentages (data completeness) of runs. **c** Overlap of the peptides quantified in at least 50% runs. **d** Distribution of the coefficient of variation (CV). CV values were calculated only for peptides quantified in at least 3 runs per sample. The median values are indicated. **e** Measured fold change (FC) values of peptide quantities using sample S3 as reference. FC values were calculated only for peptides quantified in at least 3 runs for each sample of the comparison. Numbers (*n*) of peptides are indicated for each species. The boxes mark the first and third quantile and the lines inside the boxes mark the median; the whiskers extend from the box to the farthest point lying within 1.5 times the inter-quartile range; outliers are not shown. The theoretical ratios are highlighted as dashed lines. Differences between the measured median FC values and theoretical values are indicated, among which the smallest ones are darkened. Significant differences (t-test p-value < 0.05 and Cohen's |*d*| > 0.2, two-sided, no adjustments) are indicated. Source data are provided as a Source Data file.

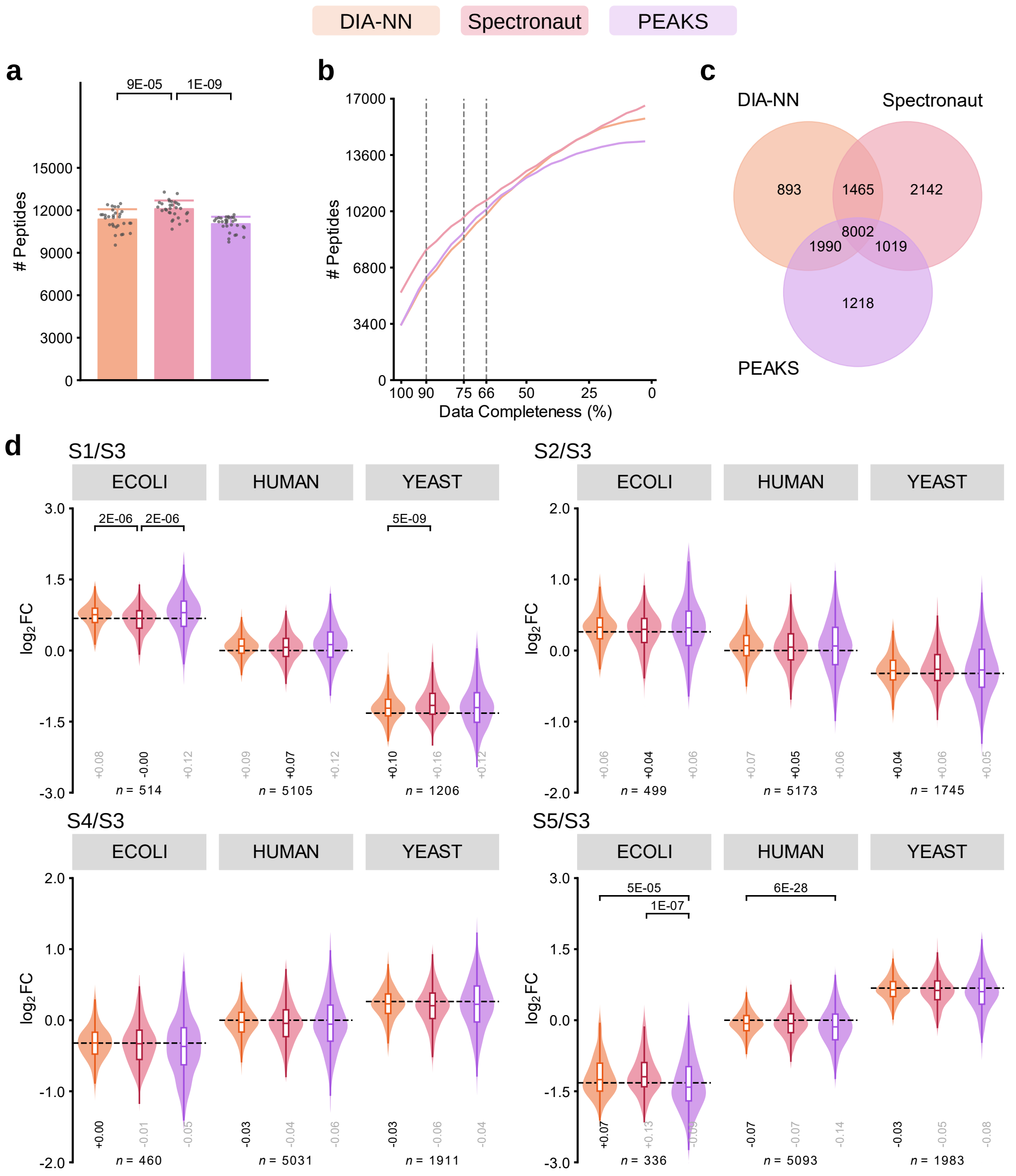

Supplementary Figure 7. Performance comparison of different DIA data analysis software tools using the library free strategy at the peptide level.

a Numbers of quantified peptides per run. The bars indicate the mean values and the error bars indicate the standard deviations. Significant differences (t-test p-value < 0.05, two-sided, no adjustments) are indicated. **b** Numbers of peptides quantified in at least specified percentages (data completeness) of runs. **c** Overlap of the peptides quantified in at least 50% runs. **d** Measured fold change (FC) values of peptide quantities using sample S3 as reference. FC values were calculated only for peptides quantified in at least 3 runs for each sample of the comparison. Numbers (*n*) of peptides are indicated for each species. The boxes mark the first and third quantile and the lines inside the boxes mark the median; the whiskers extend from the box to the farthest point lying within 1.5 times the inter-quartile range; outliers are not shown. The theoretical ratios are highlighted as dashed lines. Differences between the measured median FC values and theoretical values are indicated, among which the smallest ones are darkened. Significant differences (t-test p-value < 0.05 and Cohen's |*d*| > 0.2, two-sided, no adjustments) are indicated. Source data are provided as a Source Data file.

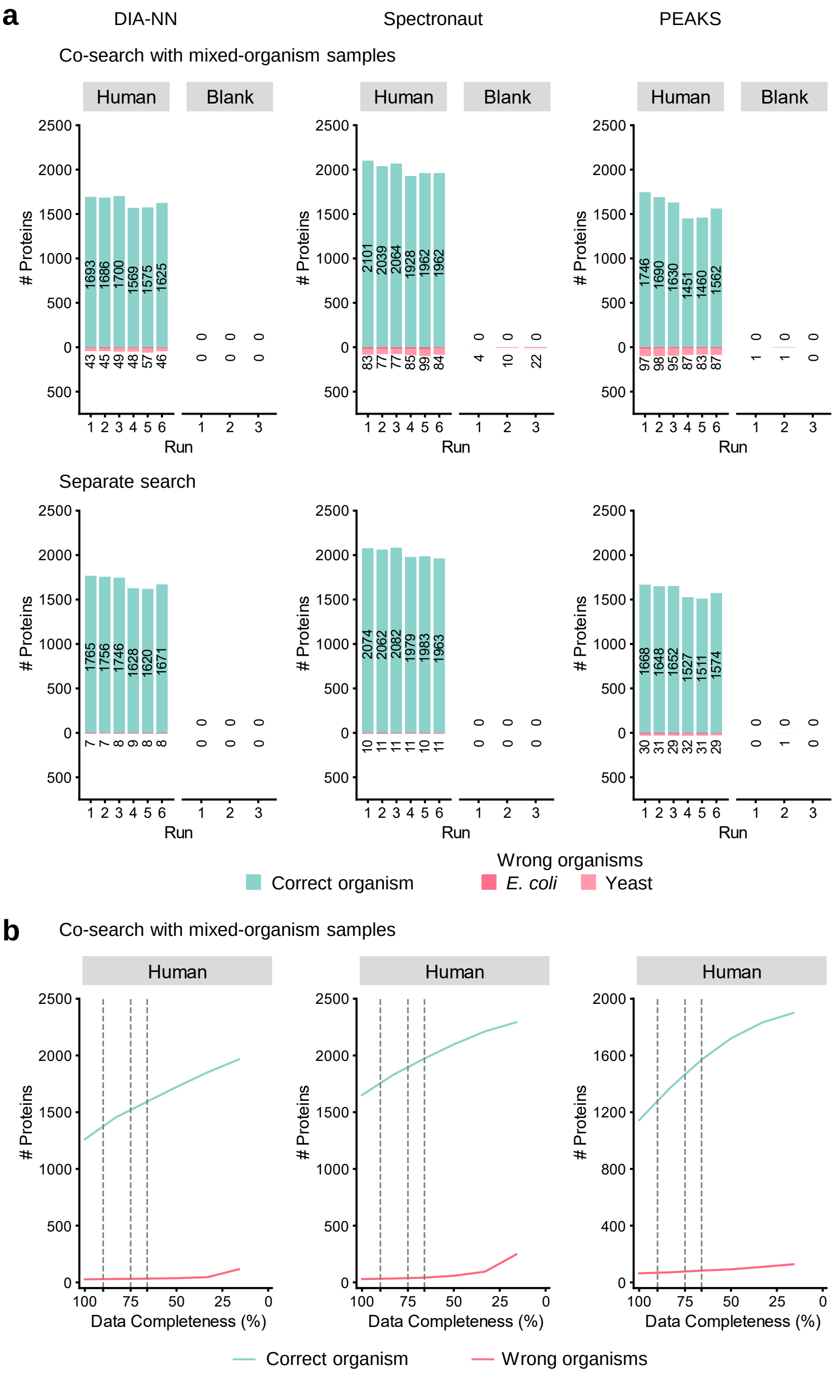

Supplementary Figure 8. Comparison of false positive detection by different software using the library free searching strategy at the protein level.

a Numbers of quantified proteins per run. For each sample, correctly detected proteins should be from the organism specific to the sample (in green), while those from other organisms (in red) are potential false positives. Results of blank injections are shown to assess potential contaminants. **b** Numbers of organism-matched (in green) and potential false positive (in red) proteins quantified in at least specified percentages (data completeness) of runs.

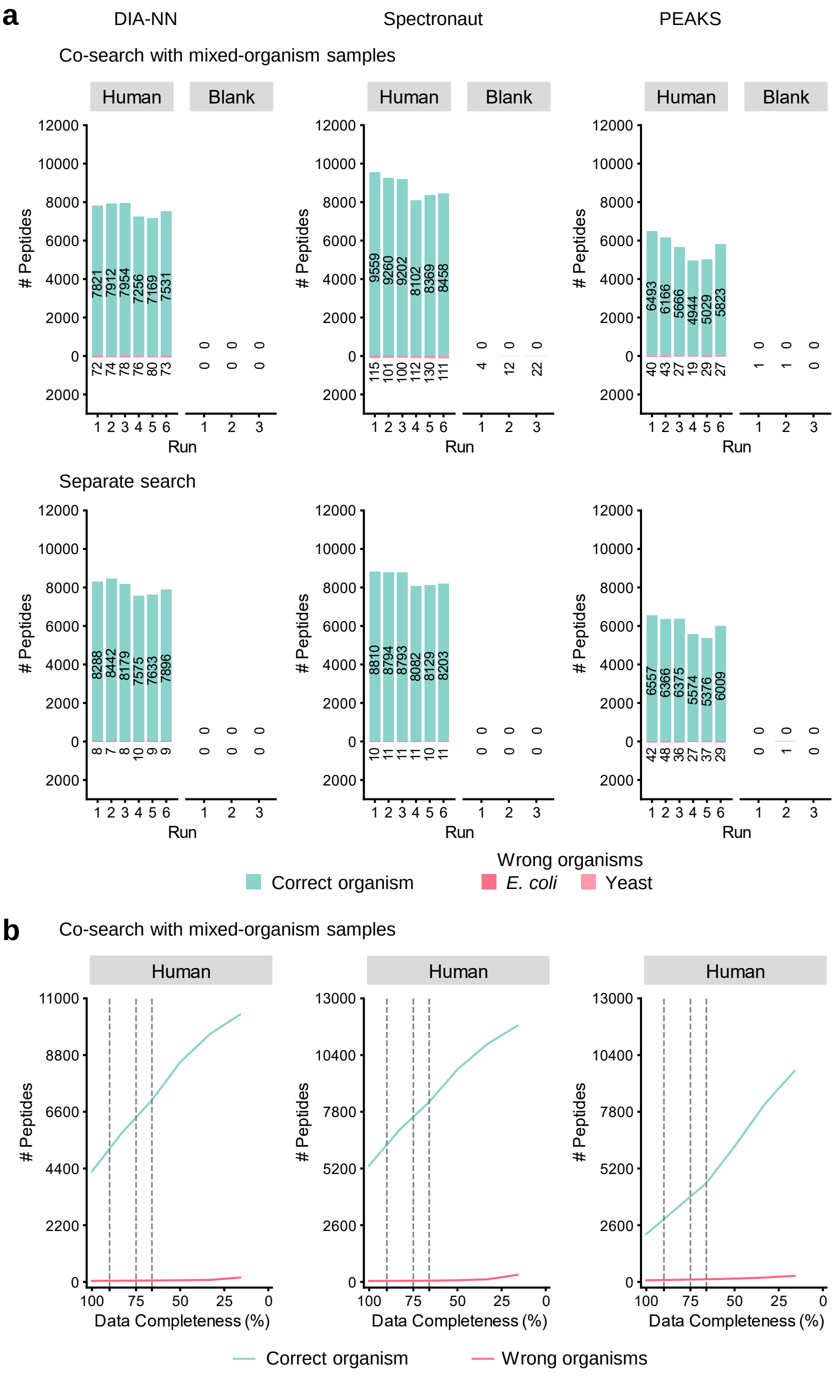

Supplementary Figure 9. Comparison of false positive detection by different software using the library free searching strategy at the peptide level.

a Numbers of quantified peptides per run. For each sample, correctly detected peptides should be from the organism specific to the sample (in green), while those from other organisms (in red) are potential false positives. Results of blank injections are shown to assess potential contaminants. **b** Numbers of organism-matched (in green) and potential false positive (in red) peptides quantified in at least specified percentages (data completeness) of runs.

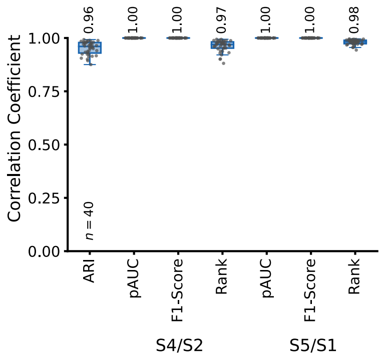

Supplementary Figure 10. Comparison of results with different clustering parameters
Correlations of the ranks and metrics of the method combinations between results obtained under varying resolution parameters (0.6, 0.8, 1.0, 1.2, 1.4), holding constant both the comparison group and the sparsity reduction conditions. The boxes mark the first and third quantile and the lines inside the boxes mark the median; the whiskers extend from the box to the farthest point lying within 1.5 times the inter-quartile range. Individual data points are overlaid as dots. The median values are indicated. Source data are provided as a Source Data file.

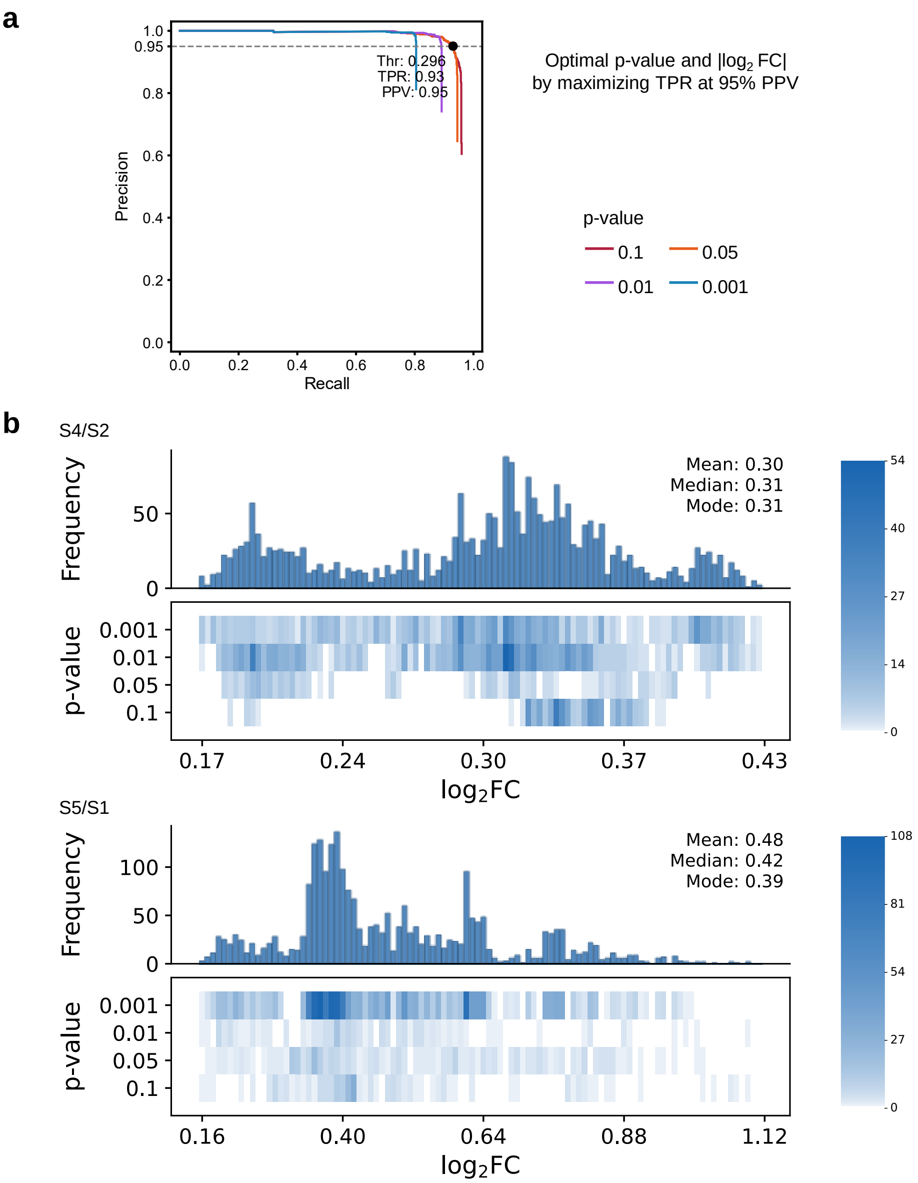

Supplementary Figure 11. Choices of p-value and fold change thresholds for differential analyses
a Example precision–recall curves using absolute values of $\log_2$ fold change (FC) as scores. Proteins with p-value ≥ a specific cut-off (indicated by line colors) are considered as negatives. The optimal cut-offs with precision (positive predictive value, PPV) > 0.95 are marked using black dots with score threshold (Thr), PPV, and TPR values indicated. **b** Distribution of the optimal |log2 FC| thresholds for the method combinations ranked top 50%.

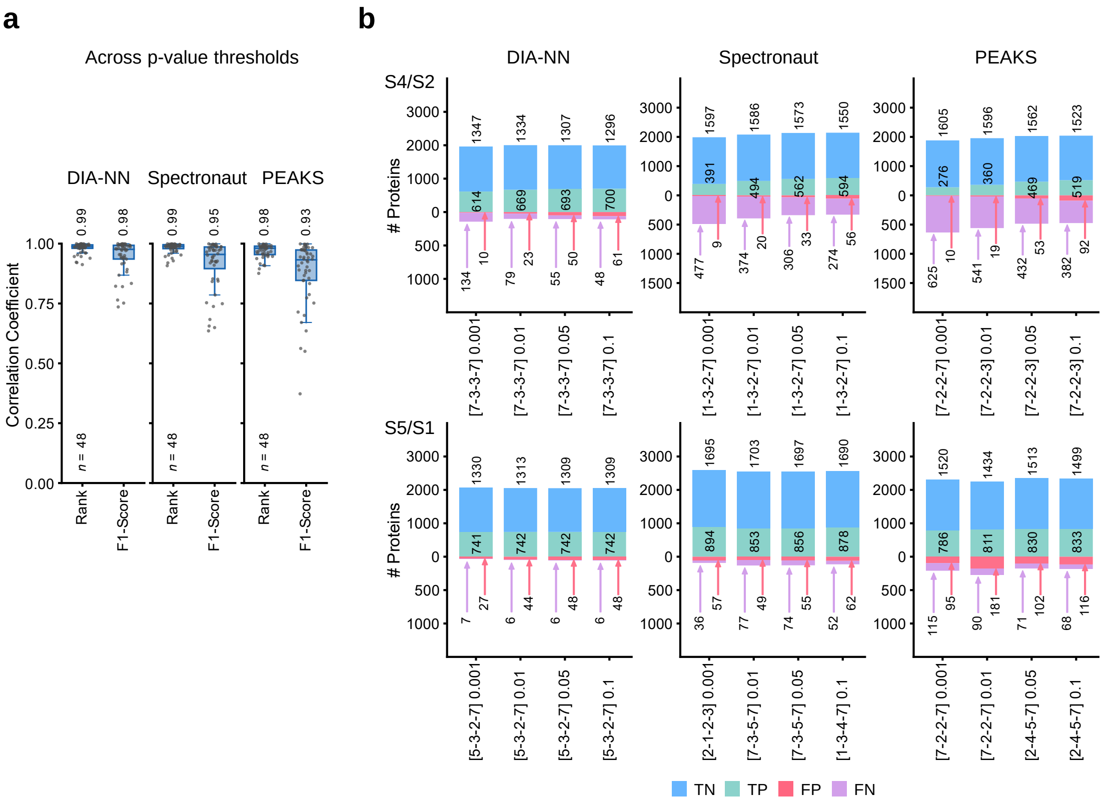

Supplementary Figure 12. Comparison of results with different p-value thresholds

a Correlations of the ranks and metrics of the method combinations between results obtained under varying p-value thresholds (0.001, 0.01, 0.05, 0.1), holding constant both the comparison group and the sparsity reduction conditions. The boxes mark the first and third quantile and the lines inside the boxes mark the median; the whiskers extend from the box to the farthest point lying within 1.5 times the inter-quartile range. Individual data points are overlaid as dots. The median values are indicated. **b** Numbers of detected proteins using different p-value thresholds, with the sparsity reduction condition fixed to SR75. The top ranked method combination starting with each sparsity reduction condition is shown, whose serial number is in brackets. Mappings of the serial numbers to detailed method combinations are present in Fig. 2a. The blue bars indicate the true negative (TN) proteins, the green bars indicate the true positive (TP) cases, the red bars indicate the false positive (FP) cases, and the purple bars indicate the false negative (FN) cases. Source data are provided as a Source Data file.

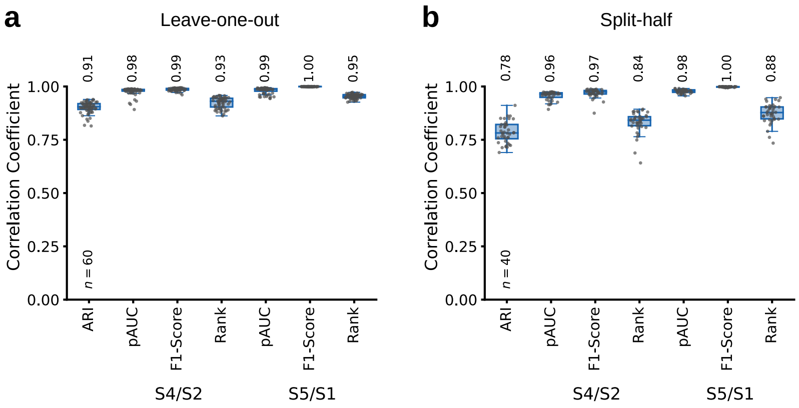

Supplementary Figure 13. Evaluation of the robustness of the metrics and ranking scheme by cross validation **a** Correlations of the ranks and metrics of the method combinations between results obtained by leave-one-out cross validation. **b** Correlations between results obtained by split-half cross validation. The boxes mark the first and third quantile and the lines inside the boxes mark the median; the whiskers extend from the box to the farthest point lying within 1.5 times the inter-quartile range. Individual data points are overlaid as dots. The median values are indicated. Source data are provided as a Source Data file.

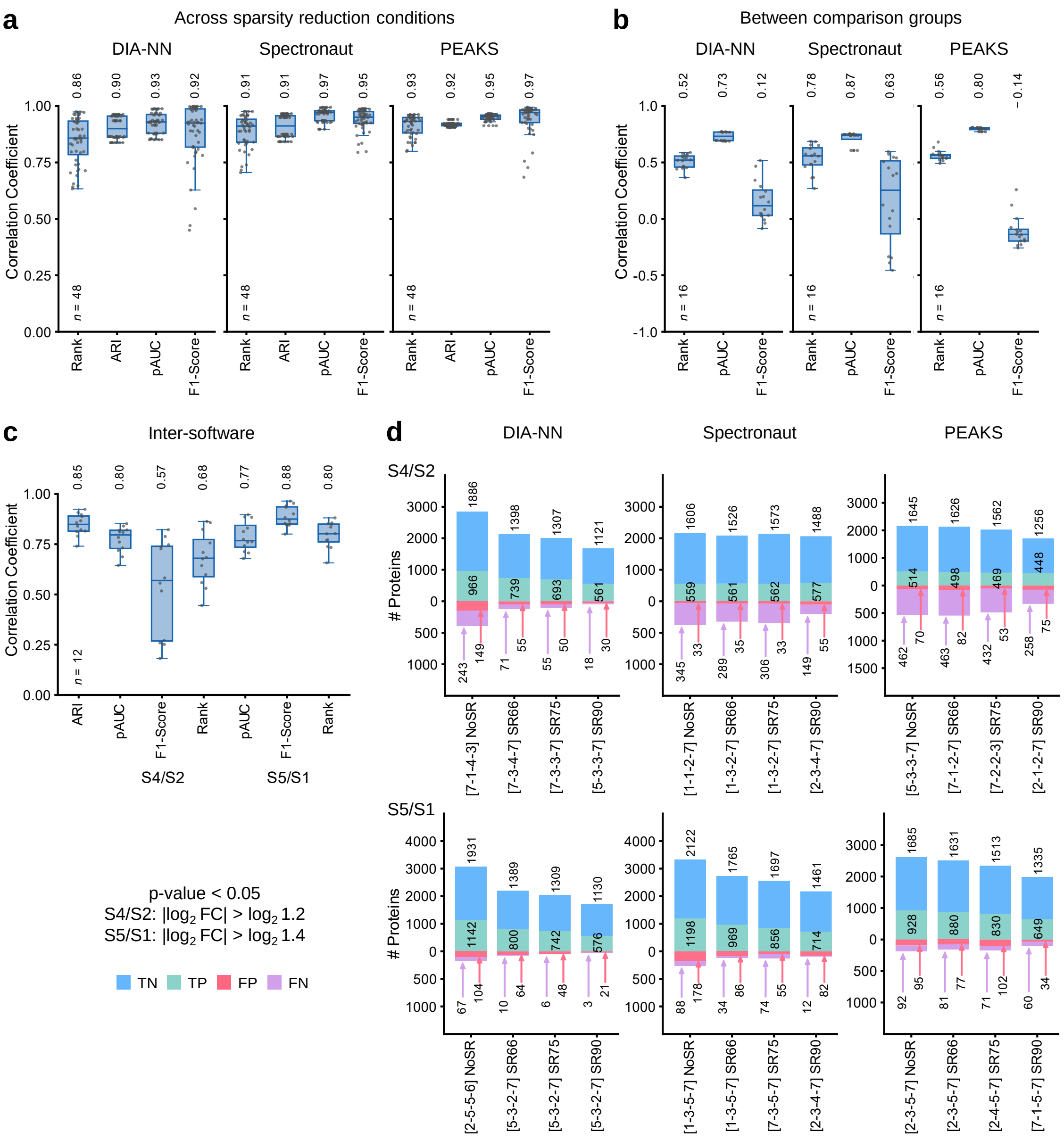

Supplementary Figure 14. Comparison of results with different software and sparsity reduction conditions
a Correlations of the ranks and metrics of the method combinations between results obtained under varying sparsity reduction conditions (0%, 66%, 75%, 90%), holding constant both the comparison group and the p-value threshold. **b** Correlations between the comparison groups (S4/S2 and S5/S1), under identical sparsity reduction conditions and p-value thresholds. **c** Correlations between results by different software. In **a**–**c**, the boxes mark the first and third quantile and the lines inside the boxes mark the median; the whiskers extend from the box to the farthest point lying within 1.5 times the inter-quartile range. Individual data points are overlaid as dots. The median values are indicated. **d** Numbers of detected proteins using different sparsity reduction conditions. The top ranked method combination starting with each sparsity reduction condition is shown, whose serial number is in brackets. Mappings of the serial numbers to detailed method combinations are present in Fig. 2a. The blue bars indicate the true negative (TN) proteins, the green bars indicate the true positive (TP) cases, the red bars indicate the false positive (FP) cases, and the purple bars indicate the false negative (FN) cases. In **c** and **d**, differential proteins are determined with p-value < 0.05. Source data are provided as a Source Data file.

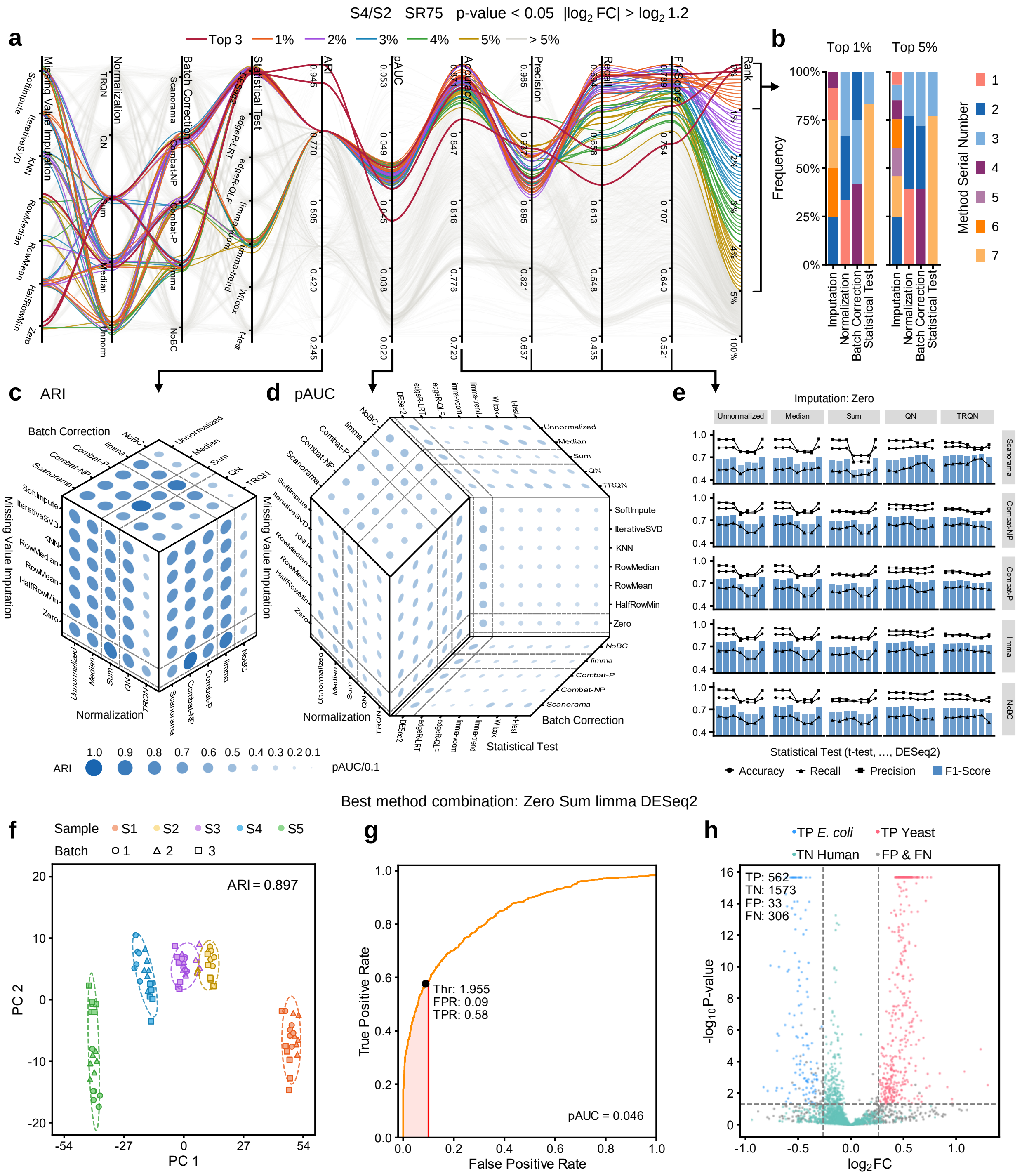

(Legend on next page)

Supplementary Figure 15. Performance comparison of method combinations for differential analysis (Spectronaut S4/S2 SR75)

a Parallel coordinate representation showing metrics using different method combinations. Line colors indicate the percentile rank of the method combinations. **b** Compositions of the top 1% and 5% method combinations in **a**. Mappings of the serial numbers to detailed methods for each step are present in Fig. 2a. **c** Adjusted Rand index (ARI) metrics. **d** Partial area under receiver operator characteristic curve (pAUC) metrics. In **c** and **d**, the metrics are visualized in a hyperbox, where each face displays the metrics with two steps variable and the other steps fixed to those of the best method combination. For the best method combination, the method choice in each step is marked with dashed lines. Dot sizes and colors indicate the metric values. **e** Accuracy (dots), recall (triangles), precision (squares), and F1-score (bars) metrics. Rows represent batch effect correction methods and columns represent normalization methods. The other steps are those of the best method combination. **f** Clustering result of the 5 groups of samples visualized using principal component analysis for dimension reduction. The fill colors indicate the sample groups and the shape indicate the batches. The border colors indicate the clusters. **g** Receiver operator characteristic (ROC) curves using −log10 p-value as scores. The optimal cut-offs with false positive rate (FPR) ≤ 0.1 are marked using black dots with score threshold (Thr), FPR, and true positive rate (TPR) values indicated. **h** Volcano plots. Blue dots represent TP *E. coli* proteins, red dots represent TP yeast proteins, green dots represent TN human proteins, and gray dots represent FP or FN proteins. For **f**–**h**, the data were processed through the best method combinations. Benchmarks are performed on protein quantification results by Spectronaut. The data are processed starting with SR75. Differential analysis was performed between the S4 and S2 sample groups. Differential proteins are determined with p-value < 0.05 and $|\log_2 FC| > \log_2 1.2$.

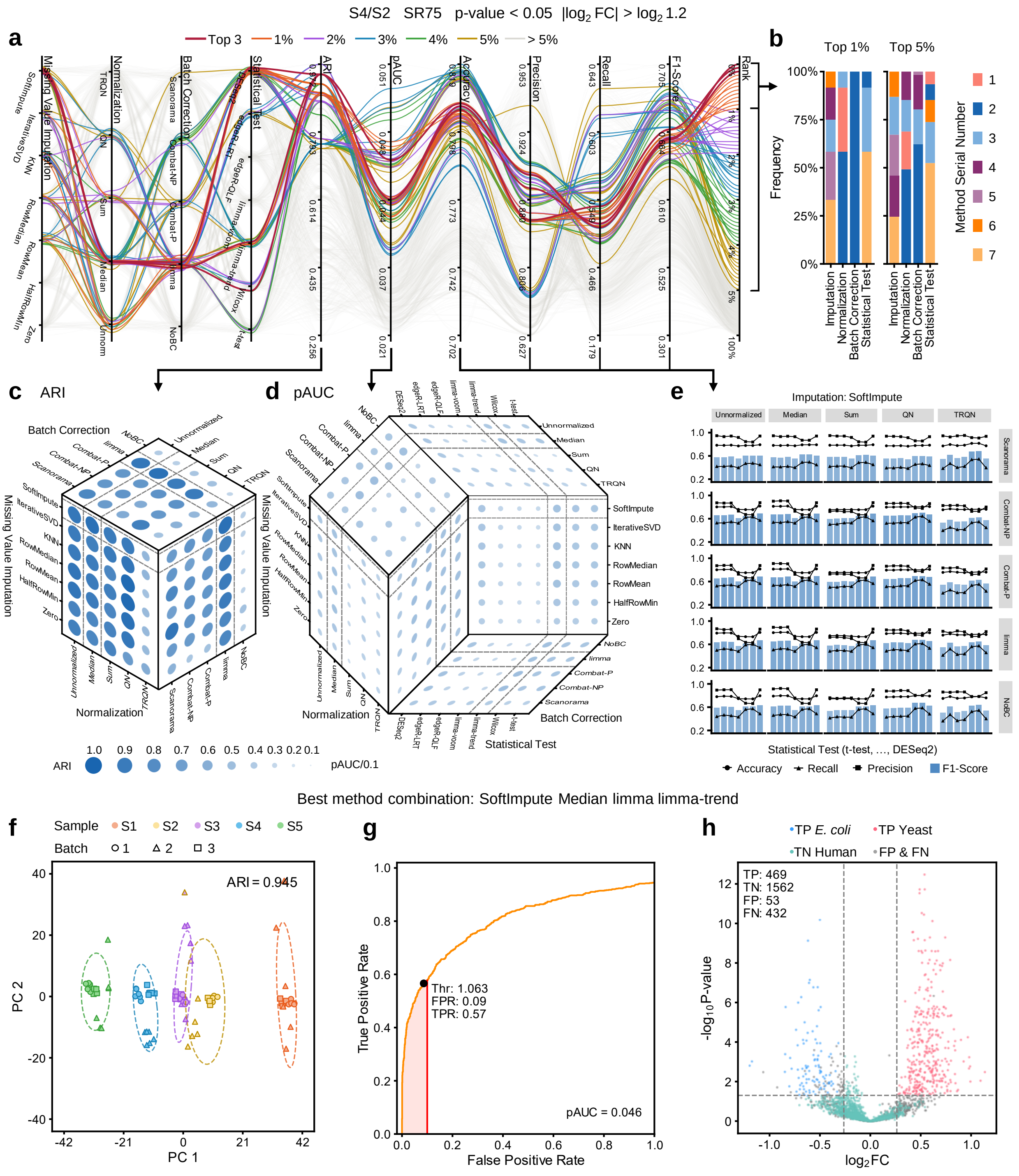

(Legend on next page)

Supplementary Figure 16. Performance comparison of method combinations for differential analysis (PEAKS S4/S2 SR75)

a Parallel coordinate representation showing metrics using different method combinations. Line colors indicate the percentile rank of the method combinations. **b** Compositions of the top 1% and 5% method combinations in **a**. Mappings of the serial numbers to detailed methods for each step are present in Fig. 2a. **c** Adjusted Rand index (ARI) metrics. **d** Partial area under receiver operator characteristic curve (pAUC) metrics. In **c** and **d**, the metrics are visualized in a hyperbox, where each face displays the metrics with two steps variable and the other steps fixed to those of the best method combination. For the best method combination, the method choice in each step is marked with dashed lines. Dot sizes and colors indicate the metric values. **e** Accuracy (dots), recall (triangles), precision (squares), and F1-score (bars) metrics. Rows represent batch effect correction methods and columns represent normalization methods. The other steps are those of the best method combination. **f** Clustering result of the 5 groups of samples visualized using principal component analysis for dimension reduction. The fill colors indicate the sample groups and the shape indicate the batches. The border colors indicate the clusters. **g** Receiver operator characteristic (ROC) curves using −log10 p-value as scores. The optimal cut-offs with false positive rate (FPR) ≤ 0.1 are marked using black dots with score threshold (Thr), FPR, and true positive rate (TPR) values indicated. **h** Volcano plots. Blue dots represent TP *E. coli* proteins, red dots represent TP yeast proteins, green dots represent TN human proteins, and gray dots represent FP or FN proteins. For **f**–**h**, the data were processed through the best method combinations. Benchmarks are performed on protein quantification results by PEAKS. The data are processed starting with SR75. Differential analysis was performed between the S4 and S2 sample groups. Differential proteins are determined with p-value < 0.05 and $|\log_2 FC| > \log_2 1.2$.

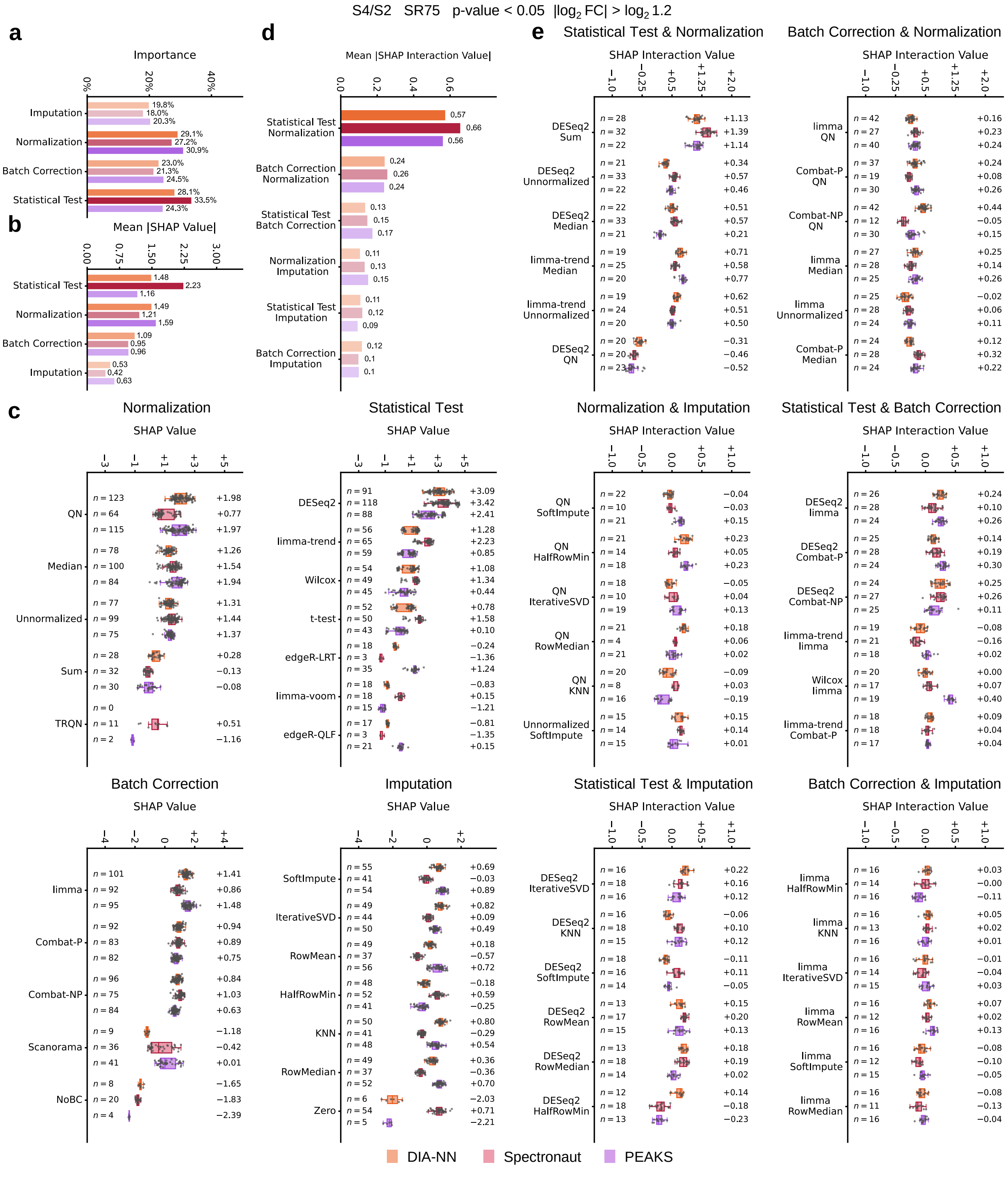

Supplementary Figure 17. Explanations of the patterns of high-performing method combinations (S4/S2 SR75) **a** Feature importance of the model. **b** Mean absolute SHAP values for each step. **c** SHAP values for the method choices in each step. **d** Mean absolute SHAP interaction values for each two steps. **e** SHAP interaction values for pairwise method choices in each two steps. In **c** and **e**, the boxes mark the first and third quantile and the lines inside the boxes mark the median; the whiskers extend from the box to the farthest point lying within 1.5 times the inter-quartile range. Individual data points are overlaid as dots. The median values and frequency (*n*) are indicated for each method choice. The data are processed starting with SR75. Differential analysis was performed between the S4 and S2 sample groups. Differential proteins are determined with p-value < 0.05 and $|\log_2 FC| > \log_2 1.2$. Only the top 25% method combinations are subjected to SHAP explanation and the method choices with $n < 3$ are not shown. Source data are provided as a Source Data file.

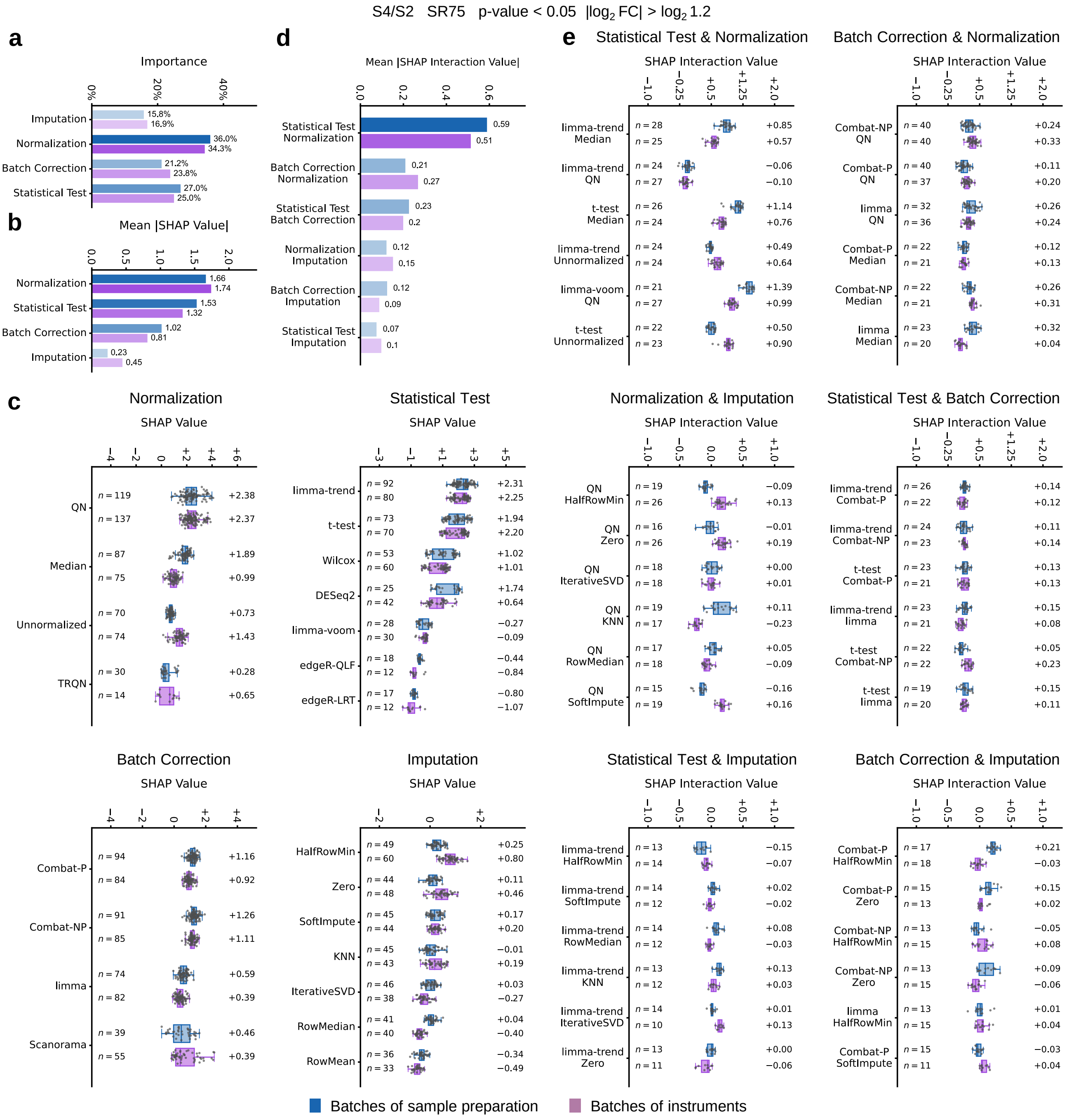

Supplementary Figure 18. Explanations of the patterns of high-performing method combinations (Spectronaut S4/S2 SR75)

a Feature importance of the model. **b** Mean absolute SHAP values for each step. **c** SHAP values for the method choices in each step. **d** Mean absolute SHAP interaction values for each two steps. **e** SHAP interaction values for pairwise method choices in each two steps. In **c** and **e**, the boxes mark the first and third quantile and the lines inside the boxes mark the median; the whiskers extend from the box to the farthest point lying within 1.5 times the inter-quartile range. Individual data points are overlaid as dots. The median values and frequency (*n*) are indicated for each method choice. Benchmarks are performed on protein quantification results by Spectronaut. The data are processed starting with SR75. Differential analysis was performed between the S4 and S2 sample groups. Differential proteins are determined with p-value < 0.05 and $|\log_2 \mathrm{FC}| > \log_2 1.2$. Only the top 25% method combinations are subjected to SHAP explanation and the method choices with $n < 3$ are not shown. Source data are provided as a Source Data file.

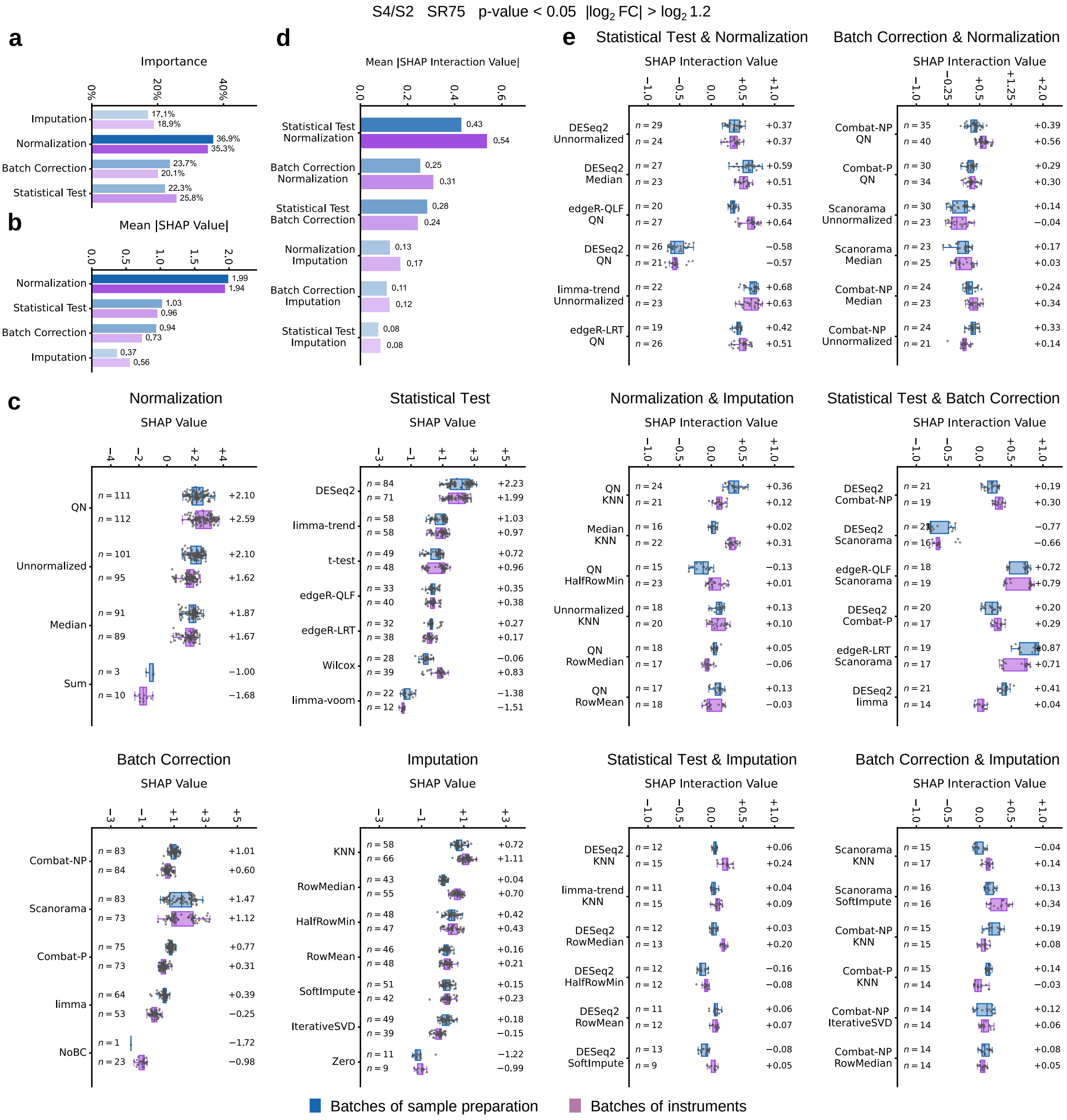

Supplementary Figure 19. Explanations of the patterns of high-performing method combinations (PEAKS S4/S2 SR75)

a Feature importance of the model. **b** Mean absolute SHAP values for each step. **c** SHAP values for the method choices in each step. **d** Mean absolute SHAP interaction values for each two steps. **e** SHAP interaction values for pairwise method choices in each two steps. In **c** and **e**, the boxes mark the first and third quantile and the lines inside the boxes mark the median; the whiskers extend from the box to the farthest point lying within 1.5 times the inter-quartile range. Individual data points are overlaid as dots. The median values and frequency (*n*) are indicated for each method choice. Benchmarks are performed on protein quantification results by PEAKS. The data are processed starting with SR75. Differential analysis was performed between the S4 and S2 sample groups. Differential proteins are determined with p-value < 0.05 and $|\log_2 \mathrm{FC}| > \log_2 1.2$. Only the top 25% method combinations are subjected to SHAP explanation and the method choices with $n < 3$ are not shown. Source data are provided as a Source Data file.

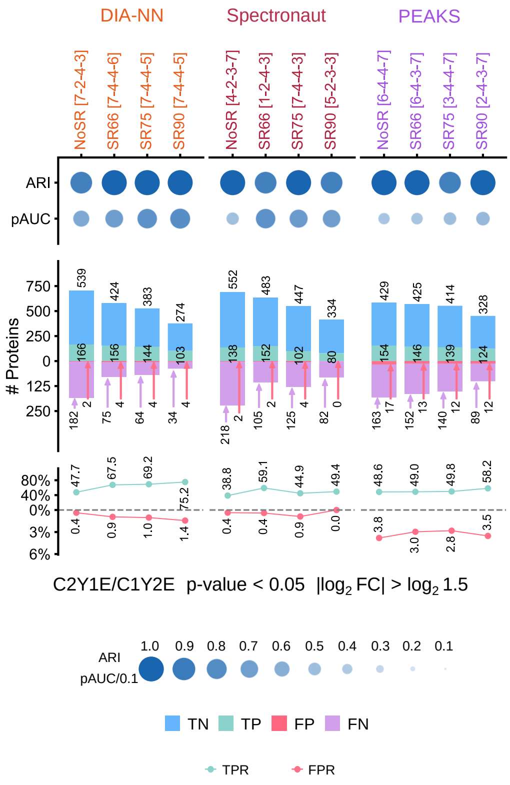

Supplementary Figure 20. Comparison of results with different software and sparsity reduction conditions on the spike-in single-cell samples

Performance of the selected high-performing method combinations on the spike-in single-cell samples. Metrics include: the ARI and pAUC values (indicated by dot sizes and colors), numbers of detected TN (blue bars), TP (green bars), FP (red bars), and FN (purple bars) proteins, as well as TPR (green lines) and FPR (red lines) values. The top ranked method combination starting with each sparsity reduction condition is shown, whose serial number is in brackets. Mappings of the serial numbers to detailed methods for each step are present in Fig 2a. The data were processed starting from SR75. Differential analysis was performed between the C2Y1E and C1Y2E sample groups. Differential proteins are determined with p-value < 0.05 and $|\log_2 FC| > \log_2 1.5$.

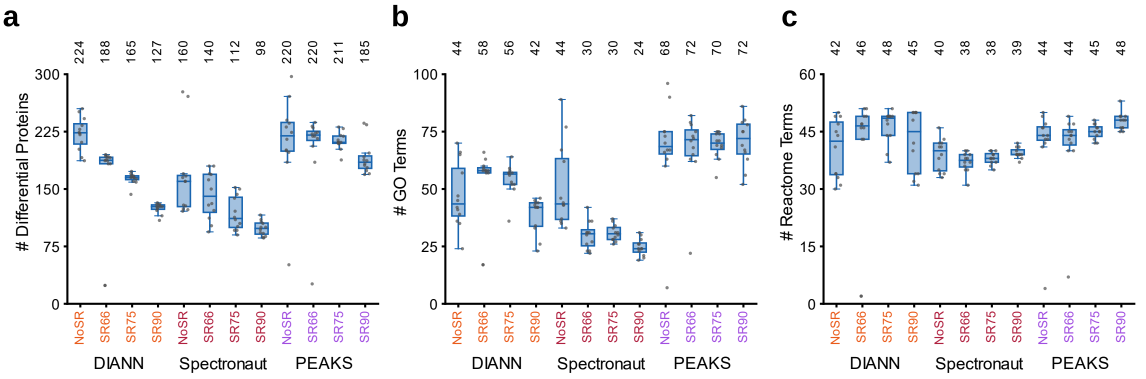

Supplementary Figure 21. Comparison of differential analysis enrichment results with different software and sparsity reduction conditions on the single-cell samples

a Number of differential proteins screened by the selected high-performing method combinations. **b** Number of enriched GO terms. **c** Number of enriched Reactome terms. The boxes mark the first and third quantile and the lines inside the boxes mark the median; the whiskers extend from the box to the farthest point lying within 1.5 times the inter-quartile range. Individual data points are overlaid as dots. The median values are indicated. Differential analysis was performed between the T2Y1E and C2Y1E sample groups. Differential proteins are determined with p-value < 0.05 and $|\log_2 FC| > \log_2 1.5$. Enriched terms are determined with p-value < 0.05. Source data are provided in Supplementary Data 6.

Supplementary Table 1. Compositions of the mixed-organism samples.

Sample \ Organism	Human	Yeast	*E. coli*
S1	50%	10%	40%
S2	50%	20%	30%
S3	50%	25%	25%
S4	50%	30%	20%
S5	50%	40%	10%

For the peptide standard samples, the total peptide concentration of the three organisms was 40 pg·µL^{-1}. The injection volume was 5 µL, corresponding to 200 pg per sample. For the samples subjected to independent digestion, 250 pg proteins in total per sample were used as starting material.

Supplementary Table 2. Compositions of the spike-in single-cell samples.

Sample \ Organism	Human	Yeast	*E. coli*
Treatment 1Y2E	1 cell (doxorubicin)	20 pg	40 pg
Treatment 2Y1E	1 cell (doxorubicin)	40 pg	20 pg
Control 1Y2E	1 cell (DMSO)	20 pg	40 pg
Control 2Y1E	1 cell (DMSO)	40 pg	20 pg

Supplementary Table 3. LC gradient.

**Vanquish NEO**		
Time/min	B phase (%)	Flow rate/(nL·min^{-1})
0	2	150
1.125	2	150
1.75	2	150
13.75	20	150
16	37	150
18.5	80	150
21	80	150
**EASY-nLC 1200**		
Time/min	B phase (%)	Flow rate/(nL·min^{-1})
0	0	150
13	40	150
14	100	150
21	100	150

Supplementary Table 4. DIA isolation windows.

Group	Center *m/z*	Width	Collision energy
1	1011.5	25	41.37
1	861.5	25	37.86
1	711.5	25	34.36
1	561.5	25	30.69
1	411.5	25	27.24
2	1036.5	25	41.76
2	886.5	25	38.25
2	736.5	25	34.76
2	586.5	25	31.27
2	436.5	25	27.82
3	1061.5	25	42.55
3	911.5	25	39.04
3	761.5	25	35.53
3	611.5	25	32.05
3	461.5	25	28.39
4	1086.5	25	43.15
4	936.5	25	39.43
4	786.5	25	35.92
4	636.5	25	32.44
4	486.5	25	28.97
5	1111.5	25	43.73
5	961.5	25	40.20
5	811.5	25	36.69
5	661.5	25	33.21
5	511.5	25	29.54
6	1136.5	25	44.12
6	986.5	25	40.60
6	836.5	25	37.09
6	686.5	25	33.59
6	536.5	25	30.12

Supplementary Table 5. DIA-NN searching parameters.

Common parameters:<br>--qvalue 0.01 --matrices<br>--met-excision --cut K*,R* --missed-cleavages 1<br>--min-pep-len 7 --max-pep-len 52 --min-pr-mz 300 --max-pr-mz 1800<br>--min-pr-charge 1 --max-pr-charge 4 --unimod4 --var-mods 1<br>--var-mod UniMod:35,15.994915,M --var-mod UniMod:1,42.010565,*n<br>--peptidoforms<br>--reanalyse --relaxed-prot-inf --rt-profiling --high-acc
Additional parameters for library-free analysis:<br>--predictor --fasta-search --min-fr-mz 200 --max-fr-mz 1800

Supplementary Table 6. Spectronaut searching parameters.

Common parameters:

**Calibration**		**Identification**	
Calibration Mode:	Automatic	Precursor Qvalue Cutoff:	0.01
MZ Extraction Strategy:	Maximum Intensity	Precursor PEP Cutoff:	0.2
Used Biognosys' iRT Kit:	False	Protein Qvalue Cutoff (Experiment):	0.01
Allow source specific iRT Calibration:	True	Protein Qvalue Cutoff (Run):	0.05
Calibration Carry-Over:	False	Protein PEP Cutoff:	0.75
Precision iRT:	True	Single Hit Definition:	By Stripped Sequence
Exclude De-amidated Peptides:	True	Exclude Single Hit Proteins:	False
iRT <-> RT Regression Type:	Local (Non-Linear) Regression	Exclude Duplicate Assays:	True
MS1 Mass Tolerance Strategy:	System Default	Exclude Predicted Fragment Scores:	False
MS2 Mass Tolerance Strategy:	System Default	Generate Decoys:	True
**Protein Inference**		Decoy Generation Method:	Mutated
Protein Inference Workflow:	Automatic	Preferred Fragment Source:	NN Predicted Fragments
Inference Algorithm:	IDPicker	Decoy Limit Strategy:	Dynamic
**Workflow**		Library Size Fraction:	0.1
Hybrid (DDA + DIA) Library:	False	Pvalue Estimator:	Kernel Density Estimator

**Quantification**			
Precursor Filtering:	Identified (Qvalue)	Quantity MS Level:	MS2
Imputation Strategy:	None	Quantity Type:	Area
Proteotypicity Filter:	None	Quantification window:	Synchronized
Protein LFQ Method:	Automatic	Major (Protein) Grouping:	by Protein Group Id
Cross-Run Normalization:	True	Minor (Peptide) Grouping:	by Stripped Sequence
Normalization Filter Type:	None	Major Group Quantity:	Mean peptide quantity
Normalization Strategy:	Automatic	Major Group Top N:	True
Row Selection:	Automatic	Max:	3
Interference Correction:	True	Min:	1
Only Identified Peptides:	True	Minor Group Quantity:	Mean precursor quantity
Exclude All Multi-Channel Interferences:	True	Minor Group Top N:	True
MS1 Min:	2	Max:	3
MS2 Min:	3	Min:	1

Additional parameters for library-free analysis:

**Identification**		**Result Filters**	
PSM FDR:	0.01	Fragment Ions:	
Peptide FDR:	0.01	Ion AA Length:	True
Protein Group FDR:	0.01	N:	3
directDIA Workflow:	directDIA+ (Deep)	Ion Charge:	False
PTM Localization Filter:	False	Ion Loss Type:	False
**Modifications**		Ion Type:	False
Max Variable Modifications:	5	m/z:	True
Fixed Modifications:	Carbamidomethyl (C)	Max:	1800
Variable Modifications:	Acetyl (Protein N-term)	Min:	200
	Oxidation (M)	Relative Intensity:	True
**Peptides**		Min:	1
Enzymes / Cleavage Rules:	Trypsin/P	Precursors:	
Digest Type:	52	Amino Acids:	False
Max Peptide Length:	7	Best N Fragments per Peptide:	True
Min Peptide Length:	1	Max:	6
Toggle N-terminal M:	True	Min:	3
**Tolerances**		Best N Peptides per Protein Group:	False
Tolerance Parameters:		Channel Count:	False
Calibration Search:	Dynamic	FASTA Matched:	False
MS1 Correction Factor:	1	Missed Cleavage:	False
MS2 Correction Factor:	1	Modifications:	None
**Workflow**		Peptide Charge:	True
Fragment Ion Selection Strategy:	Intensity Based	Max Charge:	4
In-Silico Generate Missing Channels:	False	Min Charge:	2
Use DNN Predicted Ion Mobility:	Auto	Proteotypicity:	False

Supplementary Table 7. PEAKS searching parameters.

**Spectral library search**		**Label free quantification**	
Precursor Mass Error Tolerance:	20.00 ppm	LFQ method:	Identification directed quantification
Fragment Mass Error Tolerance:	0.05 Da	DIA quantification mode:	High Accuracy
CCS Tolerance:	0.05	Retention time range:	[0.0 - Max]
Optimize Tolerance:	Yes	Base sample:	Average
Match Between Run:	Yes	Peptide Feature Filter:	
**Database search**		Avg area:	⩾ 0.0
Enzyme:	Trypsin	Quality:	⩾ 0.0
Max Missed Cleavage:	1	Charge between:	[1 - 5]
Digest Mode:	Specific	Detected in at least:	0 samples per group
Peptide Length Range:	7-30	Protein Filter:	
Precursor Charge Range:	1-4	Significance method:	ANOVA
Precursor M/Z Range:	300-1800	Modified Form:	Exclusion
Fragment M/Z Range:	200-1800	Significance:	⩾ 0.0
Fixed Modifications:	Carbamidomethylation (+57.02)	Fold change between:	[0.0 - 64.0]
Variable Modifications:	Acetylation (N-term) (+42.01)	Has at least:	0 used peptide
	Oxidation (M) (+15.99)	Normalization method:	Use TIC
Max Variable PTM Per Peptide:	1		
Contaminant Database:	N/A		
Report Filter			
Precursor FDR:	1.0%		
Protein Group FDR:	1.0%		
Proteins Unique Peptides:	≥ 0		

Specially, match between run was disabled for the analysis of the blank samples (separate search) with the AlphaPeptDeep library, because an error would occur if it was switched on.

Supplementary Note 1. Survey of DIA data analysis solutions for single-cell proteomics

We surveyed the current mainstream solutions for DIA data analysis. Strategies for DIA data analysis include library-based and library-free methods. Library-based search uses peptide spectral libraries containing prior information, e.g., retention time, ion mobility, and fragmentation patterns, and the search space is restricted to the defined peptide precursor list in the libraries. The spectral libraries are typically built from search results of DDA or DIA experiments in advance, either performed specifically for the samples or released to public repositories by the community. In silico prediction of spectral libraries by deep learning has also become popular in recent years. Library-free methods search DIA data against protein or peptide sequences without the need of external spectral libraries. Some DIA data analysis software provides built-in spectral library prediction functions and the apparent workflows do not require spectral libraries, which is classified as library-free in this study.

Three software tools were selected for performance evaluation in this study:

(1) DIA-NN[1] is one of the most popular tools of DIA data analysis in conventional and single-cell proteomics. It supports both the library-based and library-free strategies, where the library-free workflow is achieved by built-in spectral library prediction.

(2) The commercial software Spectronaut (Biognosys)[2] offers streamlined analysis workflows for library-based and library-free (directDIA) analysis. It has also gained popularity in the single-cell proteomics field.

(3) Another commercial software PEAKS (Bioinformatics Solutions)[3] has incorporated modules for DIA de novo sequencing (not used in this study), sequence database searching, and spectral library searching, forming streamlined DIA workflows since version 11.

Other software tools were not used in this study for compatibility or effectiveness reasons:

(1) MaxQuant[4] has support DIA data analysis (MaxDIA) since 2021. We tested MaxQuant using both the library-based and library-free (discovery mode) strategies. The quantified proteins were not sufficient (<200 per run in average) for statistics to

compute the benchmarking metrics.

(2) MSFragger-DIA[5] and DIA-Umpire[6] (in FragPipe), as well as EncyclopeDIA[7], are not compatible with diaPASEF data acquired on timsTOF.

(3) OpenSWATH[8] and Skyline[9] do not support library-free DIA data analysis.

(4) AlphaDIA[10] supports, in principle, fully predicted library search for diaPASEF data but it allegedly takes a long time. We tested it with a single run and estimated that it would be impractical to complete data analysis within the period of this study.

(5) diaTracer[11], integrated in the FragPipe platform, supports spectral library generation directly from diaPASEF data through pseudo-MS/MS spectra. We test it with our data but an error occurred.

To build the spectral libraries for DIA data analysis, three strategies were used int this study:

(1) Sample-specific spectral libraries (DDALib) by multiple DDA injections of individual organisms (2 ng) performed on the sample LC-MS/MS system as our DIA experiments.

(2) Spectral libraries composed from community resources (PublicLib) using timsTOF data of HeLa, yeast, and *E. coli* digests (200 ng) with high-pH reversed-phase fractionation released by Sinitcyn et al.[4] Notably, the LC and MS conditions were different from our DIA experiments.

(3) Spectral libraries at the whole-proteome scale of the organisms predicted by AlphaPeptDeep[12].

Other spectral library prediction tools, e.g., Prosit[13], were not used in this study as they cannot predict ion mobility values.

Supplementary Note 2. Intra-software performance comparison of different searching strategies

Within each software, we compared the performance for protein/peptide identification and quantification using the feasible searching strategies (**Supplementary Data 1**).

Data analysis by DIA-NN was performed by four strategies, i.e., the library-free workflow, using a sample-specific spectral library (DDALib) and using a community library (PublicLib) built by FragPipe, as well as using a whole-proteome spectral library predicted by AlphaPeptDeep.

In terms of the proteome coverage, the PublicLib strategy quantified the highest numbers of proteins (2944 ± 97, mean ± standard deviation, sic passim) and peptides (13 308 ± 901) per run among the four strategies (**Supplementary Figs. 1a** and **2a**). The DDALib (2751 ± 85 proteins and 12 738 ± 757 peptides) came in second. The results by the library-free and AlphaPeptDeep strategies were quite close (~2500 proteins and ~11 000 peptides). Regarding the data completeness, 46%–49% proteins and 19%–24% peptides were shared in all the 30 runs among those detected in at least one run. With more stringent criteria on data completeness, the difference of protein numbers reduced between PublicLib and DDALib (**Supplementary Figs. 1b**). A similar decrease of the difference was observed at the peptide level among the four strategies (**Supplementary Figs. 2b**). Considering proteins and peptides shared in at least 50% runs in each sample, the four strategies shared 63% (2168/3423) proteins and 59% (9457/16 035) peptides (**Supplementary Figs. 1c** and **2c**).

In terms of quantification, the four strategies resulted in close precision, with the median coefficient of variation (CV) values of 15.9%–18.4% for proteins and 22.8%–25.0% for peptides among replicate runs within each sample group (**Supplementary Figs. 1d** and **2d**). Fold change (FC) values protein and peptide quantities of samples S1, S2, S4, and S5 to the reference S3 were calculated based on average of the replicate runs of each sample (**Supplementary Figs. 1e** and **2e**). Proteins and peptides shared among the four strategies were used to compare the quantitative accuracy. Pairwise comparison was performed among the four strategies for each organism and each sample (resulting in 72 comparisons in total of 3 organisms with 4 samples against the

reference), where the outperforming strategy was determined with experimental median $\log_2$ FC values closer to the theoretical values and significant differences (t-test p-value < 0.05 and Cohen's $|d| > 0.2$) of the $\log_2$ FC distribution. At the protein level, the library-free workflow outperformed the others with 5 prevailing comparisons. At the peptide level, the four strategies resulted in close quantitative accuracy.

Data analysis by Spectronaut was performed by directDIA, as well as DDALib and PublicLib built by Spectronaut itself. Analysis using a whole-proteome spectral library predicted by AlphaPeptDeep failed and no results were generated.

In terms of the proteome coverage, the DDALib strategy outperformed the others with 3446 ± 187 proteins and 14 168 ± 1362 peptides quantified per run (**Supplementary Figs. 3a** and **4a**). With 75% data completeness, the numbers of proteins quantified by the DDALib (2854) and directDIA (2831) strategies were close (**Supplementary Figs. 3b** and **4b**). The PublicLib strategy resulted in the lowest number of quantified proteins (1724 ± 251 per run) and data completeness (23% shared in all the 30 runs).

In terms of quantification, the DDALib and directDIA workflows resulted in similar CV distributions (with median values of 22.2%–25.2% for proteins and 33.0%–34.5% for peptides, **Supplementary Figs. 3d** and **4d**). The PublicLib strategy led to lower CV values but it was accompanied by its lower detectable protein/peptide numbers. Regarding the quantitative accuracy, the directDIA workflow outperformed the others with 5 prevailing comparisons among the 36 pairwise comparison among the 3 strategies in total (**Supplementary Figs. 3e** and **4e**).

Data analysis by PEAKS was performed by the library-free workflow, using DDALib and PublicLib built by PEAKS itself, as well as using AlphaPeptDeep.

In terms of the proteome coverage, the DDALib strategy outperformed the others with 3286 ± 94 proteins and 13 122 ± 815 peptides quantified per run (**Supplementary Figs. 5a** and **6a**). With 75% data completeness, the numbers of proteins quantified by the DDALib (3062) and PublicLib (2965) strategies were still higher than those by the library-free (2728) and AlphaPeptDeep (2718) strategies (**Supplementary Figs. 5b**). Considering proteins and peptides shared in at least 50% runs in each sample, the four

strategies shared 62% (2420/3925) proteins and 48% (8741/18 027) peptides (**Supplementary Figs. 5c** and **6c**).

In terms of quantification, the four strategies resulted in similar precision (with median CV values of 25.8%–30.5% for proteins and 41.9%–45.0% for peptides, **Supplementary Figs. 5d** and **6d**) and accuracy (resulting in a tie in every comparison, **Supplementary Figs. 5e** and **6e**).

Supplementary Note 3. Estimation of error rates by the entrapment strategy

The samples with single organisms were searched against the spectral libraries of the containing all the three organisms. Proteins and peptides originated from organisms absent in these samples were used to evaluate the level of potential false positive detection in the results. The error rate was estimated for each sample group as[14]

$$Error\ rate = \frac{E_{\mathrm{id}}}{E_{\mathrm{id}} + S_{\mathrm{id}}} \cdot \frac{E_{\mathrm{lib}} + S_{\mathrm{lib}}}{E_{\mathrm{lib}}} \cdot \pi_0 \qquad \text{(S3-1)}$$

where S_{id} is the number of identified organism-specific proteins or peptides for each sample, and E_{id} is the number of identified entrapment proteins or peptides that are not expected to be present in the sample. S_{lib} and E_{lib} are the respective numbers of organism-specific and entrapment proteins peptides in the spectral library. The π_0 (prior probability of incorrect identification) correction factor was calculated as

$$\pi_0 = \frac{E_{\mathrm{lib}} + S_{\mathrm{lib}} - 0.99\, S_{\mathrm{id}}}{E_{\mathrm{lib}} + S_{\mathrm{lib}}} \qquad \text{(S3-2)}$$

where the factor is set as 0.99 since S_{id} is the number of identified organism-specific proteins or peptides at 1% q-value.

Considering the identified entrapment analytes that were originated from potential common contaminants in sample preparation or carry-over in LC analysis, blank samples were inserted among the single-organism samples. For the peptide standard samples, 3 blank LC-MS injections were performed; for the independent-digestion samples, 3 blank samples were subjected to digestion before LC-MS analysis (samples 1–3) and another 3 blank LC-MS injections were performed (samples 4–6). The entrapment analytes identified from the blank samples were fewer than those from the single-organism samples, indicating the latter (at least a large proportion of them) were not among the contaminants from digestion or LC carry-over (**Supplementary Figs. 8a** and **9a**). Notably, such blank samples did not necessarily represent the degree of contaminant presence in real single-cell proteome samples, and thus only non-human entrapment identifications in human samples were used to evaluate the error rates.

To calculate the error rates, the S_{id} and E_{id} were adjusted by considering the potential contaminants identified in the blank samples. Proteins detected in the blank samples using any software and searching strategies were combined to a contaminant set. For

each run, the sample-specific analytes were the organism-specific analytes added with the wrong-organism analytes present in the contaminant set. The entrapment analytes were the wrong-organism analytes not present in the contaminant set. The S_{id} and E_{id} were set as the average number of the sample-specific and entrapment analytes per run. Since the contaminants are negligible compared to the library size, S_{lib} and E_{lib} were not adjusted. For the library-free strategy, S_{lib} and E_{lib} were set using those counted from the AlphaPeptDeep library for approximation.

The calculation process and results of the error rates are present in **Supplementary Data 1** and **2**.

Supplementary Note 4. Survey of methods for batch effect correction and differential expression analyses

We surveyed the popular solutions for batch effect correction and differential expression analyses.

For sparsity reduction, we adopted the settings reported by a benchmarking study for bulk DIA proteomics[15], namely, no sparsity reduction (NoSR), requiring >66% values per protein (SR66), and requiring >90% values per protein (SR90). Based on the assessment of protein coverage and error rates in this study, we added another choice of requiring >75% values per protein (SR75).

For missing value imputation, we applied 7 commonly used methods for omics data analysis, which can be classified into three types: single value methods, including (1) zero[16], (2) half row minimum (HalfRowMin), (3) row mean (RowMean), (4) and row median (RowMedian); local similarity methods, including (5) K-nearest neighbors (KNN)[17]; global structure methods, including (6) iterative low-rank singular value decomposition (IterativeSVD)[17] and (7) soft-thresholded singular value decomposition (SoftImpute)[18].

For normalization, we adopted the settings reported by the benchmarking study for bulk DIA proteomics[15], namely, (1) unnormalized, (2) median normalization, (4) quantile normalization (QN), and (5) tail-robust quantile normalization (TRQN)[19]. We also added (3) the sum normalization that are commonly used in MS-based proteomics.

For batch effect correction, we adopt 4 methods that are commonly used in single-cell transcriptomics[20]. The R package (2) limma[21] fits the input data to a linear model with a blocking term to capture the batch effects. In the R package sva, ComBat[22] fits the standardized data to standard distributions using a Bayesian approach to estimate the batch effects present. ComBat can be run in two modes, where (3) the parametric mode (ComBat-P) includes scale adjustments whereas (4) the non-parametric mode (ComBat-NP) only corrects the mean of the batch effect. The Python package (5) Scanorama[23] searches for mutual nearest neighbors between batches and merges data batches into panoramas using a weighted average of vectors between local matching cells. Additionally, (1) no batch correction (NoBC) was used for comparison. Other

popular tools were not used in this study for compatibility or effectiveness reasons. Harmony[24] and fastMNN[25] output dimensionally reduced embedding; BBKNN[26] outputs a connectivity graph of cells. While results of these tools are usable for clustering and visualization, they are not suitable for differential expression analysis in proteomics as they do not output a corrected expression matrix. Seurat[27] was not used since our dataset did not reach the minimum sample size required.

For statistical tests, we applied 7 popular methods for differential protein discovery in proteomics, including (1) Welch's t-test, (2) Wilcoxon–Mann–Whitney test, (3–4) limma, (5–6) edgeR, and (7) DESeq2. The R package limma[21] was originally designed for analyzing RNA microarray data, while edgeR[28] and DESeq2[29] were originally developed for processing RNA-seq data. Several methods have been built into limma, such as (3) limma-trend and (4) limma-voom. In edgeR, statistical methods include (5) the likelihood ratio test (edgeR-LRT) and (6) the quasi-likelihood F-test (edgeR-QLF). Most methods accept log2 transformed data as an input. limma-voom, edgeR, and DESeq2 require data rescaled to normal RNA-seq range. Moreover, DESeq2 only accepts integer data. These pipelines have been transferred to quantitative proteomics analysis[30].

Supplementary Note 5. Choices of the clustering parameters

For performance evaluation of batch effect correction, the samples were clustered using the Louvain algorithm implemented in the R package Seurat. The clustering function FindClusters has a resolution parameter to control the number of communities (larger using a value above 1.0 or smaller using a value below 1.0). We surveyed the resolution from 0.6 to 1.4 stepped by 0.2. The Pearson correlation coefficient (PCC) of the ranks and metrics of method combinations was computed across the resolution parameter. A median PCC of 0.96 was achieved for the ARI values and 0.98 for the ranks (**Supplementary Fig. 10**), indicating that ranking was stable regardless of the resolution parameter.

Therefore, a resolution parameter of 1.0 was used in all the analyses.

Supplementary Note 6. Choices of p-value and fold change thresholds for differential analyses

We performed a preliminary benchmarking to survey the p-value and fold change thresholds. After the calculation of pAUC from the ROC curve, common choices for significance levels 0.1, 0.05, 0.01, and 0.001 were selected as p-value thresholds. For each p-value threshold, a precision–recall curve was obtained with the $|\log_2 FC|$ threshold varying. For each method combination, the optimal p-value and $|\log_2 FC|$ thresholds were selected to maximize the recall at 95% precision. Other performance metrics including accuracy values and F1-scores were computed based on the optimal $|\log_2 FC|$ and p-value thresholds.

For the choice of the $|\log_2 FC|$ threshold, the method combinations ranked top 50% were selected and the distribution of their optimal $|\log_2 FC|$ thresholds are visualized in **Supplementary Fig. 11**. The final $|\log_2 FC|$ threshold was 0.3 ($\approx \log_2 1.2$) for the differential analyses between sample groups S4 and S2, and 0.5 ($\approx \log_2 1.4$) for S5 and S1.

As a well-accepted threshold, we mainly reported the results with p-value < 0.05. In addition, benchmarking was also performed with other p-value thresholds. The Pearson correlation coefficient (PCC) of the ranks of method combinations was computed across the p-value thresholds. A median PCC of 0.99 was achieved (**Supplementary Fig. 12a**), indicating that ranking was stable regardless of p-value thresholds. Stricter p-value thresholds resulted in slightly fewer false positives but did not lead to significant changes (**Supplementary Fig. 12b**).

Supplementary Note 7. Cross validation of the benchmarking metrics and ranking scheme.

We evaluated the robustness of the ARI and other metrics by cross validation.

(1) Leave-one-out cross validation. For each time n from 1 to 6, the n-th run in each batch and each sample group was excluded, resulting in 6 datasets. The Pearson correlation coefficient (PCC) of the ranks and metrics of method combinations was computed in 10 pairwise comparisons of the results of 6 datasets for each of the 4 sparsity reduction criteria (resulting in 60 values in total). A median PCC of 0.91 was achieved for the ARI values and 0.94 for the ranks (**Supplementary Fig. 13a**).

(2) Split-half cross validation. The runs in each batch and each sample group were randomly split into two balanced groups, forming two subsets. The PCC values were computed between the results of the two subsets for each of the 4 sparsity reduction criteria. This procedure was repeated 10 times (resulting in 40 values in total). A median PCC of 0.78 was achieved for the ARI values and 0.86 for the ranks (**Supplementary Fig. 13b**).

The results indicated the robustness of the metrics and ranking scheme.

Supplementary Note 8. Benchmarking workflows to screen differential proteins between other sample groups

In addition to S4 and S2, we also benchmarked the statistical test methods for screening differential proteins between sample groups S5 and S1 (**Supplementary Data 3**). The expected alteration of yeast and *E. coli* proteins between S5 and S1 (4-fold variation) was larger than those between S4 and S2 (2-fold variation). As a matter of course, the larger fold change can facilitate the discrimination of differential proteins from the unchanged ones and thus the S5/S1 comparison should be an easier task than S4/S2. Still, we focused on interpreting the results of SR75.

For DIA-NN, the top 1% method combinations concentrated mostly in KNN or RowMean for missing value imputation, coupled with sum or median normalization, as well as limma, Combat-NP, or Combat-P for batch effect correction. DESeq2 was the optimal method for statistical test. The best method combination resulted in an ARI of 1.0, a pAUC of 0.085, and a recall of 99% at the precision of 94%.

For Spectronaut, the top 1% method combinations concentrated mostly in HalfRowMin for missing value imputation, coupled with sum or no normalization, limma or Combat-NP for batch effect correction, as well as DESeq2 and limma-trend for statistical test. The best method combination resulted in an ARI of 0.77, a pAUC of 0.080, and a recall of 92% at the precision of 94%.

For PEAKS, the top 1% method combinations concentrated mostly in IterativeSVD, RowMean, or SoftImpute for missing value imputation, coupled with sum, median, quantile, or no normalization, Scanorama or limma for batch effect correction, as well as DESeq2 for statistical test. The best method combination resulted in an ARI of 0.92, a pAUC of 0.066, and a recall of 92% at the precision of 89%.

The Pearson correlation coefficient (PCC) was used to evaluate consistency of the ranks of method combinations between the comparisons S5/S1 and S4/S2 (**Supplementary Fig. 14b**). The median of PCC was 0.52–0.78 for the three software. The results suggested that the ranking of method combinations was sensitive to the variation between sample groups, and thus the choice of analysis methods should consider the magnitude of effects in real biological studies. However, considering the

top 1% method combinations, the optimal methods for batch effect correction and statistical test were relatively stable.

We further explored the performance of differential expression analysis between sample groups with smaller alteration (using the workflow with adjustable fold change thresholds in **Supplementary Note 6**). The top ranked method combinations for the comparisons S2/S3 and S4/S3 (1.2- to 1.25-fold variation) resulted in <60% recall at the precision of 95%. The low recall was reasonable as the actual fold change reached the limit of quantification precision (~20% CV shown in **Supplementary Fig. 1d**). Moreover, experimental designs with smaller effect sizes require a much larger number of samples to achieve adequate statistical power. Indeed, fold change thresholds ≤1.25 are rare in bulk or single cell proteomics studies.

Supplementary Note 9. Benchmarking workflows without covariates.

For limma and Combat, users can specify the outcome of interest in the model matrix to provide biological covariates to the methods. However, Scanorama does not use biological covariates. Although these covariates are given in the experimental settings of this study, they are not always available in single-cell data. Therefore, we tested how limma and Combat perform if no biological covariates are provided to the model.

In the original method combinations, limma, Combat-P, and Combat-NP were replaced with their no-covariate versions (denoted as limma-NC, Combat-P-NC, and Combat-P-NC). To compare the performance with and without covariates, benchmarking results without covariates were ranked combined with the original ones. We focused on the results of S4/S2 with SR75. The method combinations were split into 3 groups, i.e., limma/Combat with covariates, limma/Combat without covariates, and Scanorama and NoBC, and the top 1% method combinations within each group are visualized in **Supplementary Data 4** (panels b and c in Figures SD4-1 for DIA-NN, SD4-2 for Spectronaut, SD4-3 for PEAKS). The no-covariate versions of limma/Combat underperformed those with covariates with a slight gap, while they were still better than Scanorama.

To simulate the scenario that covariates are not available, benchmarking results without covariates (using limma-NC, Combat-P-NC, Combat-P-NC, Scanorama, and NoBC) were ranked. The variation of the metrics with different method choices are visualized in Figures SD4-1, SD4-2, and SD4-3 (panels d, e, and f). The method combinations with limma-NC yielded high ARI and pAUC values. Results of other sparsity reduction criteria and comparison groups are presented in Figures SD4-7 to SD4-27.

Supplementary Note 10. Benchmarking workflows with no imputation

A strategy in some proteomics studies is to ignore missing values for differential analyses. Therefore, we tested the performance with no imputation whenever allowed by the batch correction and statistical test methods used. In these benchmarking workflows, missing values were kept as they were (KeepNA). Batch correction methods included NoBC and limma, and statistical test methods included t-test, Wilcox, and limma-trend. Other methods were excluded since they do not support missing values.

To compare the performance with and without imputation, benchmarking results with KeepNA were ranked combined with the original ones. We focused on the results of S4/S2 with SR75. The top 1% method combinations with and without imputation are visualized in **Supplementary Data 4** (Figures SD4-4 for DIA-NN, SD4-5 for Spectronaut, SD4-6 for PEAKS), showing the gap between them. The low performance of the method combinations without imputation may be due to the limited choices of batch correction and statistical test methods. Other the other hand, with the method choices in other steps fixed, the benchmarking metrics without imputation were still lower than those with imputation. Results of other sparsity reduction criteria and comparison groups are presented in Figures SD4-28 to SD4-48.

Supplementary References

1. Demichev, V., Messner, C. B., Vernardis, S. I., Lilley, K. S. & Ralser, M. DIA-NN: neural networks and interference correction enable deep proteome coverage in high throughput. *Nat. Methods* **17**, 41-44 (2020).
2. Bekker-Jensen, D. B. et al. Rapid and site-specific deep phosphoproteome profiling by data-independent acquisition without the need for spectral libraries. *Nat. Commun.* **11**, 787 (2020).
3. Tran, N. H. et al. Deep learning enables de novo peptide sequencing from data-independent-acquisition mass spectrometry. *Nat. Methods* **16**, 63-66 (2019).
4. Sinitcyn, P. et al. MaxDIA enables library-based and library-free data-independent acquisition proteomics. *Nat. Biotechnol.* **39**, 1563-1573 (2021).
5. Yu, F. et al. Analysis of DIA proteomics data using MSFragger-DIA and FragPipe computational platform. *Nat. Commun.* **14**, 4154 (2023).
6. Tsou, C.-C. et al. DIA-Umpire: comprehensive computational framework for data-independent acquisition proteomics. *Nat. Methods* **12**, 258-264 (2015).
7. Searle, B. C. et al. Generating high quality libraries for DIA MS with empirically corrected peptide predictions. *Nat. Commun.* **11**, 1548 (2020).
8. Röst, H. L. et al. OpenSWATH enables automated, targeted analysis of data-independent acquisition MS data. *Nat. Biotechnol.* **32**, 219-223 (2014).
9. Egertson, J. D., MacLean, B., Johnson, R., Xuan, Y. & MacCoss, M. J. Multiplexed peptide analysis using data-independent acquisition and Skyline. *Nat. Protoc.* **10**, 887-903 (2015).
10. Wallmann, G. et al. AlphaDIA enables end-to-end transfer learning for feature-free proteomics. Preprint at https://doi.org/10.1101/2024.05.28.596182 (2024).
11. Li, K., Teo, G. C., Yang, K. L., Yu, F. & Nesvizhskii, A. I. diaTracer enables spectrum-centric analysis of diaPASEF proteomics data. *Nat. Commun.* **16**, 95 (2025).
12. Zeng, W.-F. et al. AlphaPeptDeep: a modular deep learning framework to predict peptide properties for proteomics. *Nat. Commun.* **13**, 7238 (2022).
13. Gessulat, S. et al. Prosit: proteome-wide prediction of peptide tandem mass spectra by deep learning. *Nat. Methods* **16**, 509-518 (2019).
14. Demichev, V. et al. dia-PASEF data analysis using FragPipe and DIA-NN for deep proteomics of low sample amounts. *Nat. Commun.* **13**, 3944 (2022).
15. Fröhlich, K. et al. Benchmarking of analysis strategies for data-independent acquisition proteomics using a large-scale dataset comprising inter-patient heterogeneity. *Nat. Commun.* **13**, 2622 (2022).
16. Lazar, C., Gatto, L., Ferro, M., Bruley, C. & Burger, T. Accounting for the multiple natures of missing values in label-free quantitative proteomics data sets to compare imputation Strategies. *J. Proteome Res.* **15**, 1116-1125 (2016).
17. Troyanskaya, O. et al. Missing value estimation methods for DNA microarrays. *Bioinformatics* **17**, 520-525 (2001).
18. Mazumder, R., Hastie, T. & Tibshirani, R. Spectral regularization slgorithms for learning large incomplete matrices. *J. Mach. Learn. Res.* **11**, 2287–2322 (2010).
19. Brombacher, E., Schad, A. & Kreutz, C. Tail-robust quantile normalization.

Proteomics **20**, 2000068 (2020).
20. Tran, H. T. N. et al. A benchmark of batch-effect correction methods for single-cell RNA sequencing data. *Genome Biol.* **21**, 12 (2020).
21. Smyth, G. K. & Speed, T. Normalization of cDNA microarray data. *Methods* **31**, 265-273 (2003).
22. Johnson, W. E., Li, C. & Rabinovic, A. Adjusting batch effects in microarray expression data using empirical Bayes methods. *Biostatistics* **8**, 118-127 (2007).
23. Hie, B., Bryson, B. & Berger, B. Efficient integration of heterogeneous single-cell transcriptomes using Scanorama. *Nat. Biotechnol.* **37**, 685-691 (2019).
24. Korsunsky, I. et al. Fast, sensitive and accurate integration of single-cell data with Harmony. *Nat. Methods* **16**, 1289-1296 (2019).
25. Haghverdi, L., Lun, A. T. L., Morgan, M. D. & Marioni, J. C. Batch effects in single-cell RNA-sequencing data are corrected by matching mutual nearest neighbors. *Nat. Biotechnol.* **36**, 421-427 (2018).
26. Polański, K. et al. BBKNN: fast batch alignment of single cell transcriptomes. *Bioinformatics* **36**, 964-965 (2020).
27. Hao, Y. et al. Dictionary learning for integrative, multimodal and scalable single-cell analysis. *Nat. Biotechnol.* **42**, 293-304 (2024).
28. McCarthy, D. J., Chen, Y. & Smyth, G. K. Differential expression analysis of multifactor RNA-Seq experiments with respect to biological variation. *Nucleic Acids Res.* **40**, 4288-4297 (2012).
29. Love, M. I., Huber, W. & Anders, S. Moderated estimation of fold change and dispersion for RNA-seq data with DESeq2. *Genome Biol.* **15**, 550 (2014).
30. Lin, M.-H. et al. Benchmarking differential expression, imputation and quantification methods for proteomics data. *Brief. Bioinform.* **23**, bbac138 (2022).
