# Supplementary material for: Benchmarking informatics workflows for data-independent acquisition single-cell proteomics
Source: Nat Commun. 2025 Nov 21;16:10276. doi: 10.1038/s41467-025-65174-4 (PMC12639053; doi:10.1038/s41467-025-65174-4)
Supplement: Supplementary file 2 — Description of Additional Supplementary File [file 41467_2025_65174_MOESM2_ESM.pdf]

The Description of Additional Supplementary Files

Supplementary Data 1: Performance comparison of DIA data analysis software with different searching strategies on the mixedorganism samples of peptide standards.

Supplementary Data 2: Performance comparison of DIA data analysis software with different searching strategies on the mixedorganism samples with independent digestion.

Supplementary Data 3: Benchmarking results of different method combinations on the mixed-organism peptide standards.

Supplementary Data 4: Benchmarking results of different method combinations without covariates and without imputation on the mixed-organism peptide standards.

Supplementary Data 5: Benchmarking results of different method combinations performed on the simulated single-cell samples with independent digestion.

Supplementary Data 6: Testing high-performing method combinations on spike-in single-cell samples.

Supplementary Data 7: Enrichment results of differential proteins screened using the high-performing method combinations.
