# Supplementary material for: Benchmarking informatics workflows for data-independent acquisition single-cell proteomics
Source: Nat Commun. 2025 Nov 21;16:10276. doi: 10.1038/s41467-025-65174-4 (PMC12639053; doi:10.1038/s41467-025-65174-4)
Supplement: Supplementary file 3 — Supplementary Data 1 [file 41467_2025_65174_MOESM3_ESM.zip › FigSD1-[7-18] Identification & Quantification.pdf]

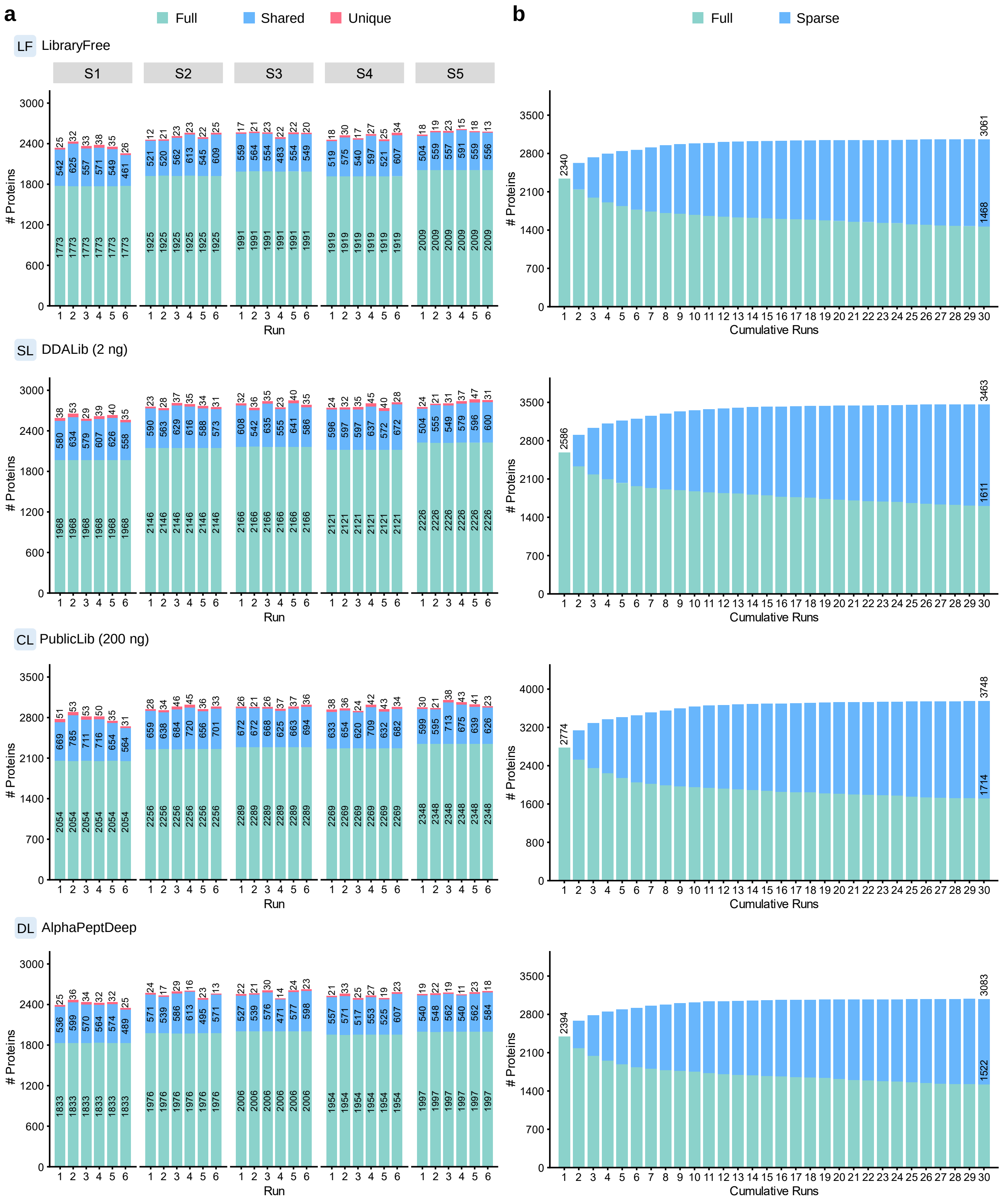

Figure SD1-7. Comparison of detection capabilities by different searching strategies using DIA-NN at the protein level.

a Numbers of quantified proteins per run. Full proteins (in green) represent those quantified in all the runs of a sample; shared proteins (in blue) represent those quantified in 2 bot not all runs of a sample; unique proteins (in red) represent those quantified in only 1 run. **b** Numbers of cumulative proteins from run 1 to 30 (in the order of samples S1–S5 and replicates 1–6 for each sample). Full proteins (in green) represent those shared in the cumulative runs; sparse proteins (in blue) represent those quantified in at least 1 run in the cumulative runs.

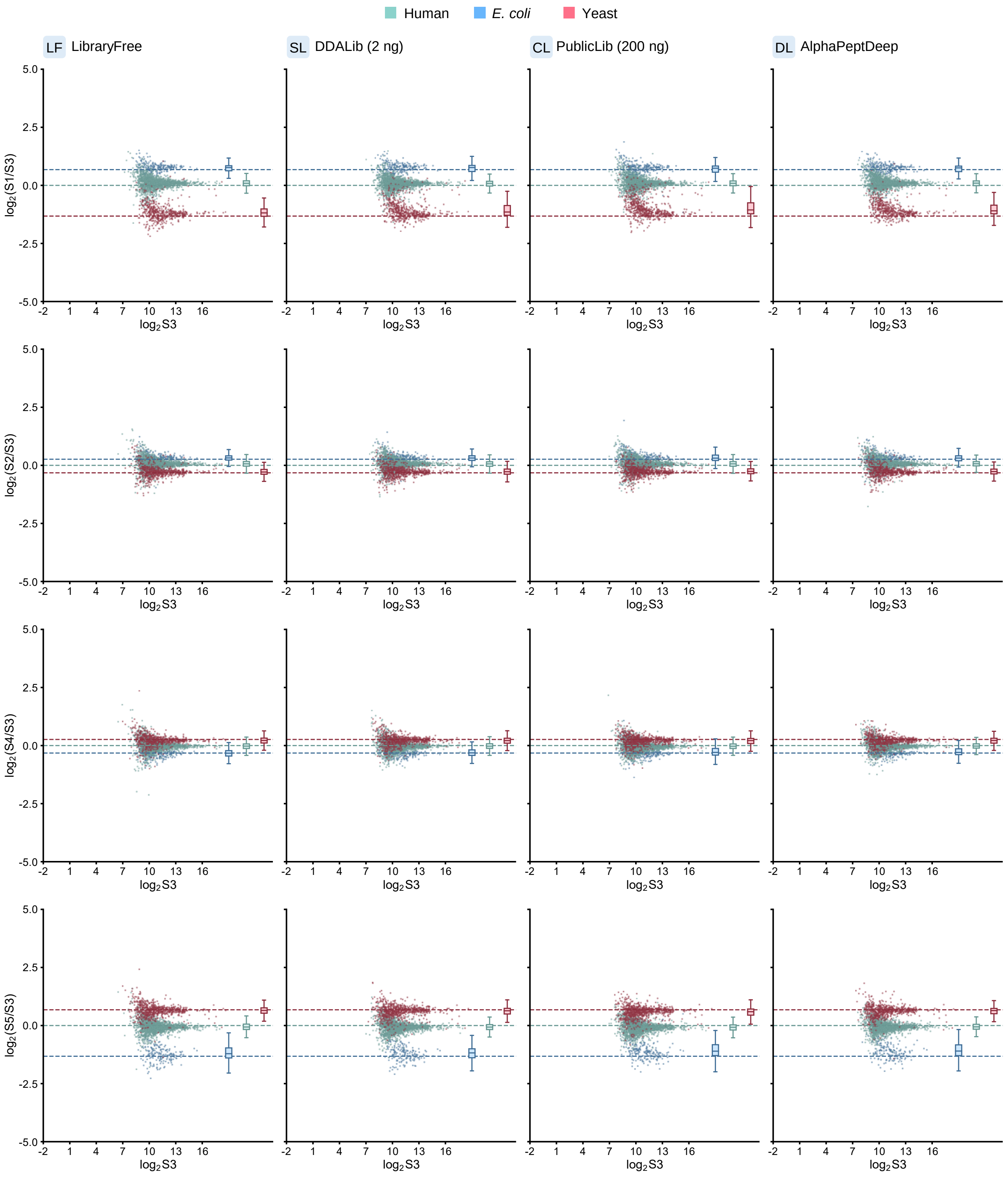

Figure SD1-8. Comparison of quantitative accuracies by different searching strategies using DIA-NN at the protein level.

Measured fold change (FC) values of protein quantities using sample S3 as reference. FC values were calculated only for proteins quantified in at least 3 runs for each sample of the comparison. The boxes mark the first and third quantile and the lines inside the boxes mark the median; the whiskers extend from the box to the farthest point lying within 1.5 times the inter-quartile range. The theoretical ratios are highlighted as dashed lines. Colors indicate proteins from human (in green), yeast (in red), and *E. coli* (in blue).

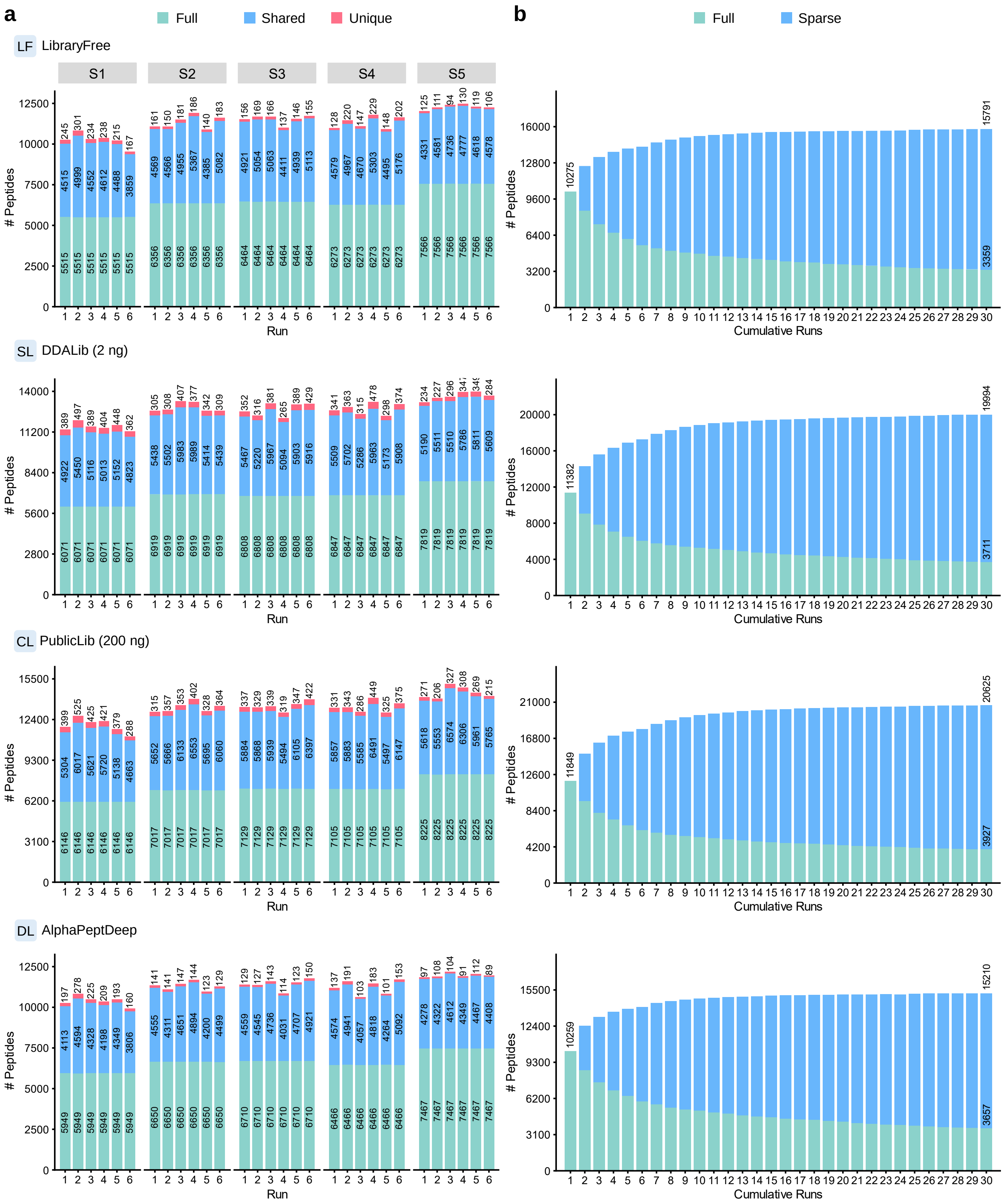

Figure SD1-9. Comparison of detection capabilities by different searching strategies using DIA-NN at the peptide level.

a Numbers of quantified peptides per run. Full peptides (in green) represent those quantified in all the runs of a sample; shared peptides (in blue) represent those quantified in 2 bot not all runs of a sample; unique peptides (in red) represent those quantified in only 1 run. **b** Numbers of cumulative peptides from run 1 to 30 (in the order of samples S1–S5 and replicates 1–6 for each sample). Full peptides (in green) represent those shared in the cumulative runs; sparse peptides (in blue) represent those quantified in at least 1 run in the cumulative runs.

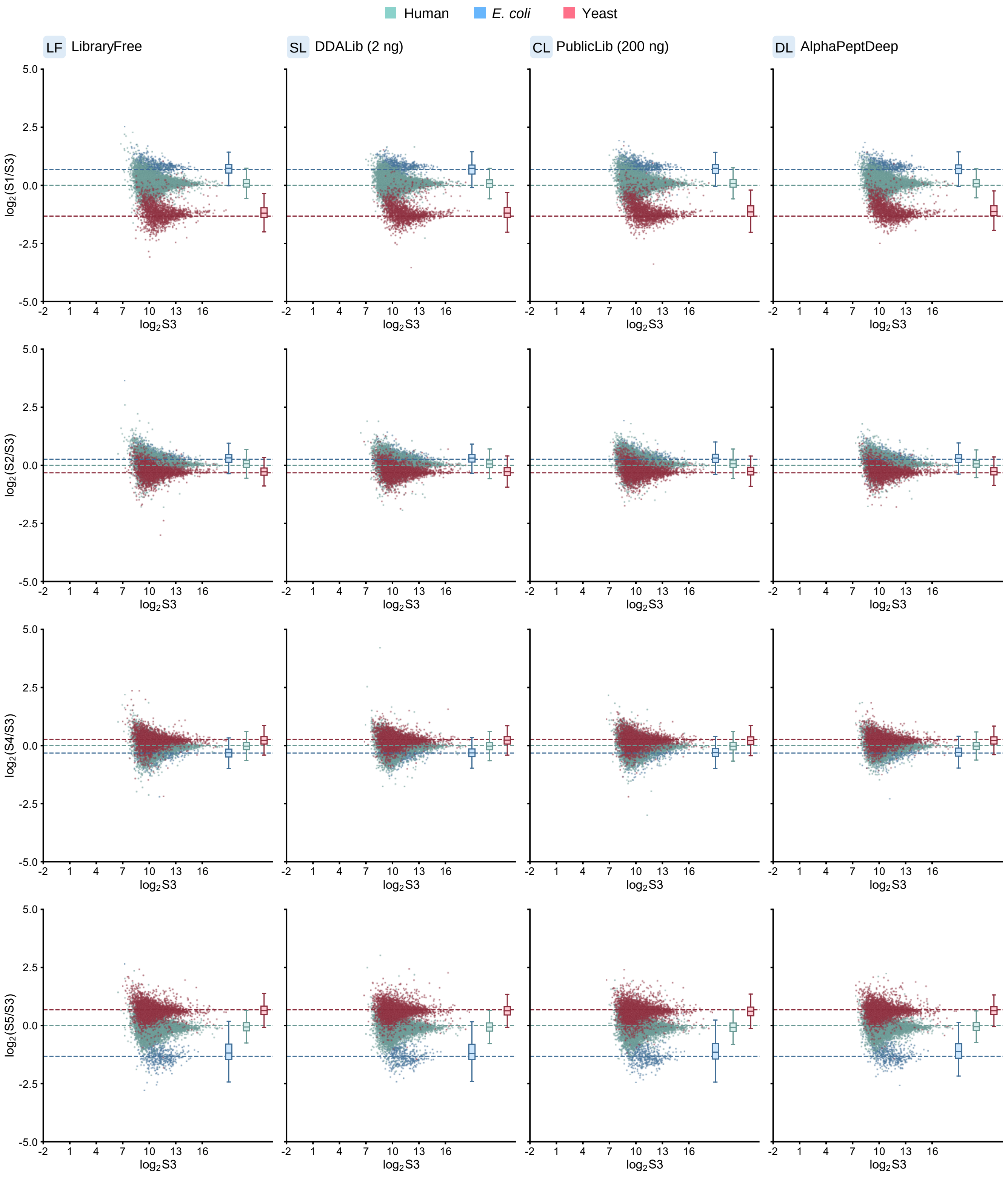

Figure SD1-10. Comparison of quantitative accuracies by different searching strategies using DIA-NN at the peptide level.

Measured fold change (FC) values of peptide quantities using sample S3 as reference. FC values were calculated only for peptides quantified in at least 3 runs for each sample of the comparison. The boxes mark the first and third quantile and the lines inside the boxes mark the median; the whiskers extend from the box to the farthest point lying within 1.5 times the inter-quartile range. The theoretical ratios are highlighted as dashed lines. Colors indicate peptides from human (in green), yeast (in red), and *E. coli* (in blue).

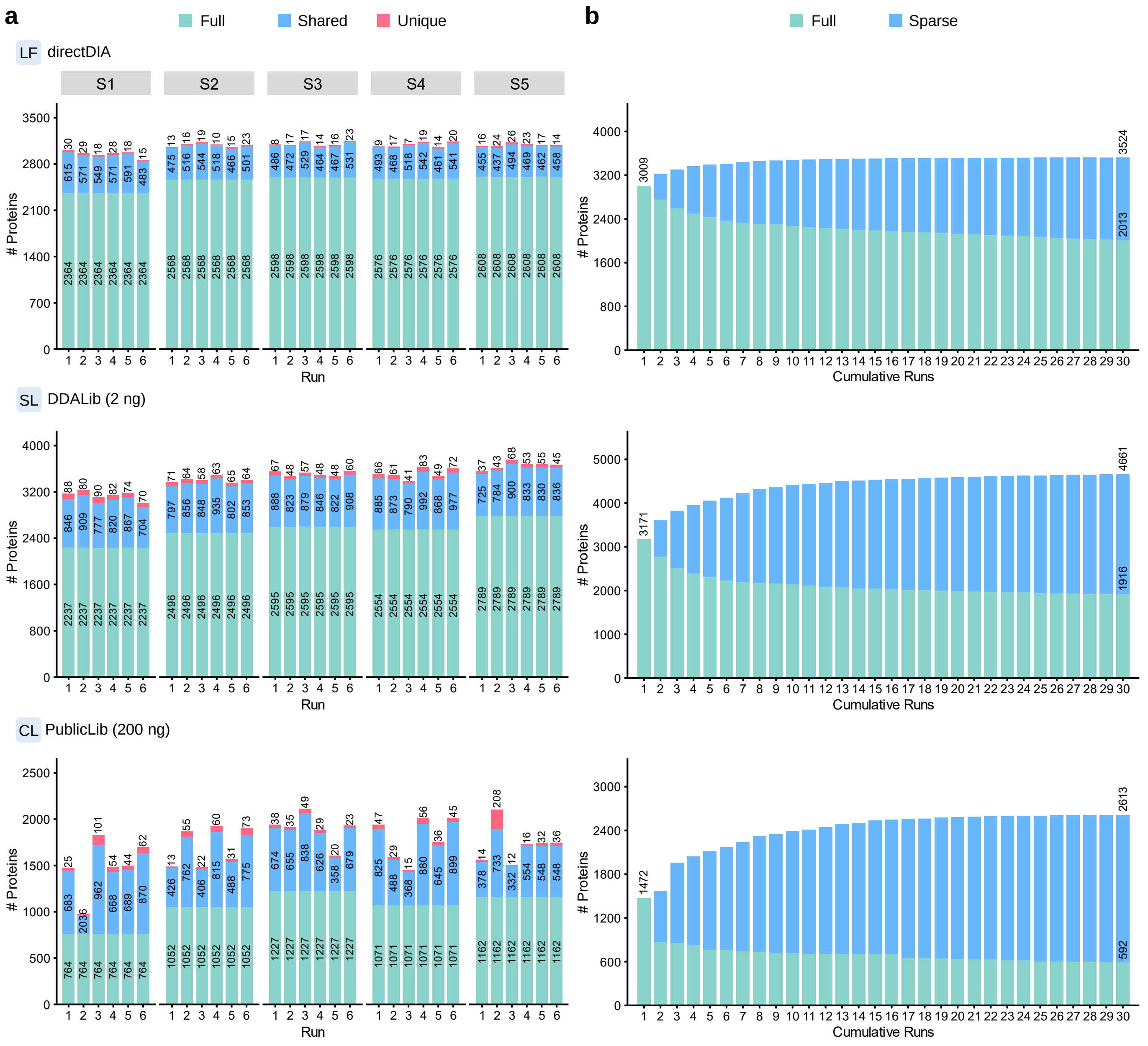

Figure SD1-11. Comparison of detection capabilities by different searching strategies using Spectronaut at the protein level.

a Numbers of quantified proteins per run. Full proteins (in green) represent those quantified in all the runs of a sample; shared proteins (in blue) represent those quantified in 2 bot not all runs of a sample; unique proteins (in red) represent those quantified in only 1 run. **b** Numbers of cumulative proteins from run 1 to 30 (in the order of samples S1–S5 and replicates 1–6 for each sample). Full proteins (in green) represent those shared in the cumulative runs; sparse proteins (in blue) represent those quantified in at least 1 run in the cumulative runs.

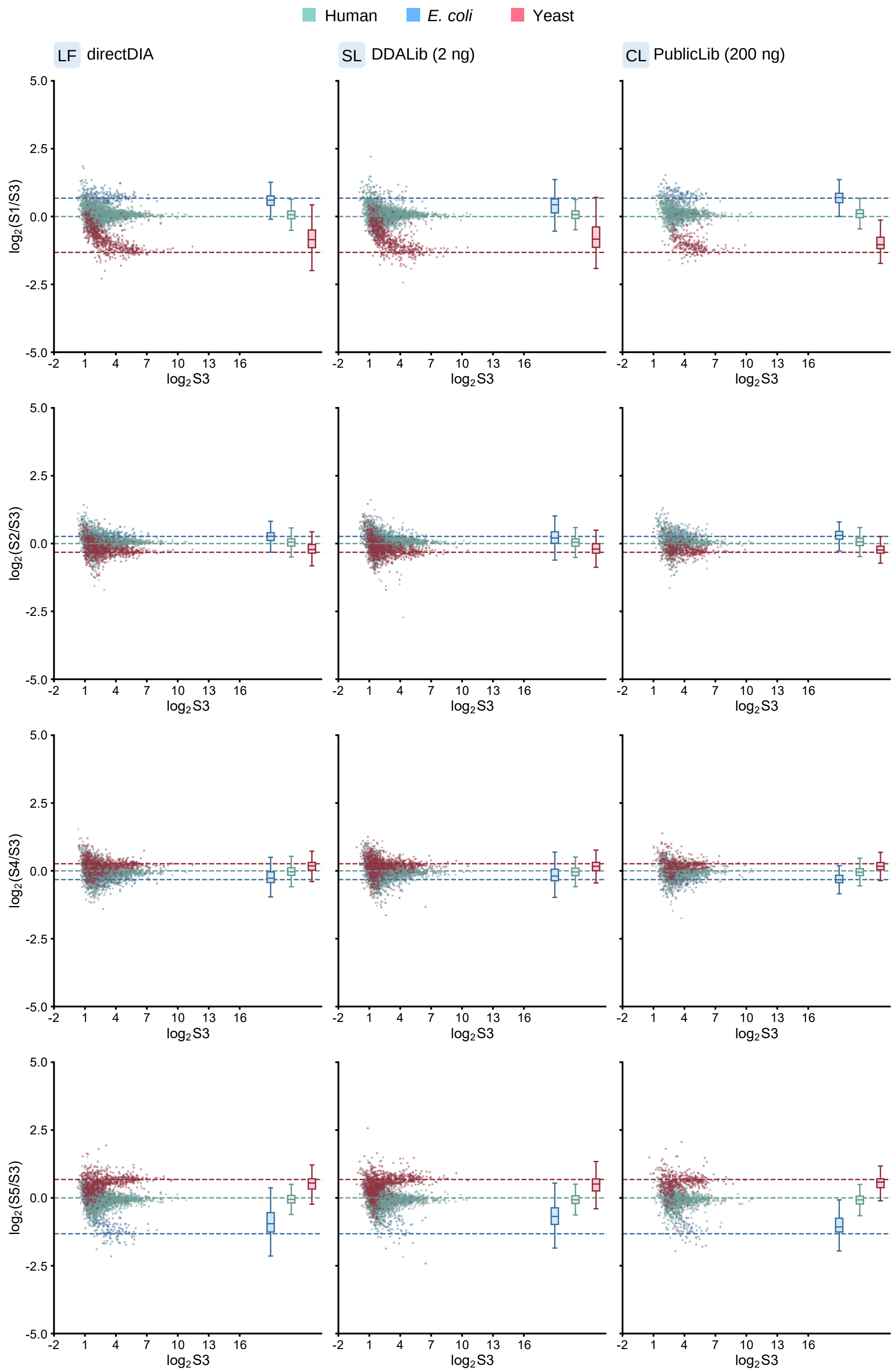

Figure SD1-12. Comparison of quantitative accuracies by different searching strategies using Spectronaut at the protein level.

Measured fold change (FC) values of protein quantities using sample S3 as reference. FC values were calculated only for proteins quantified in at least 3 runs for each sample of the comparison. The boxes mark the first and third quantile and the lines inside the boxes mark the median; the whiskers extend from the box to the farthest point lying within 1.5 times the inter-quartile range. The theoretical ratios are highlighted as dashed lines. Colors indicate proteins from human (in green), yeast (in red), and *E. coli* (in blue).

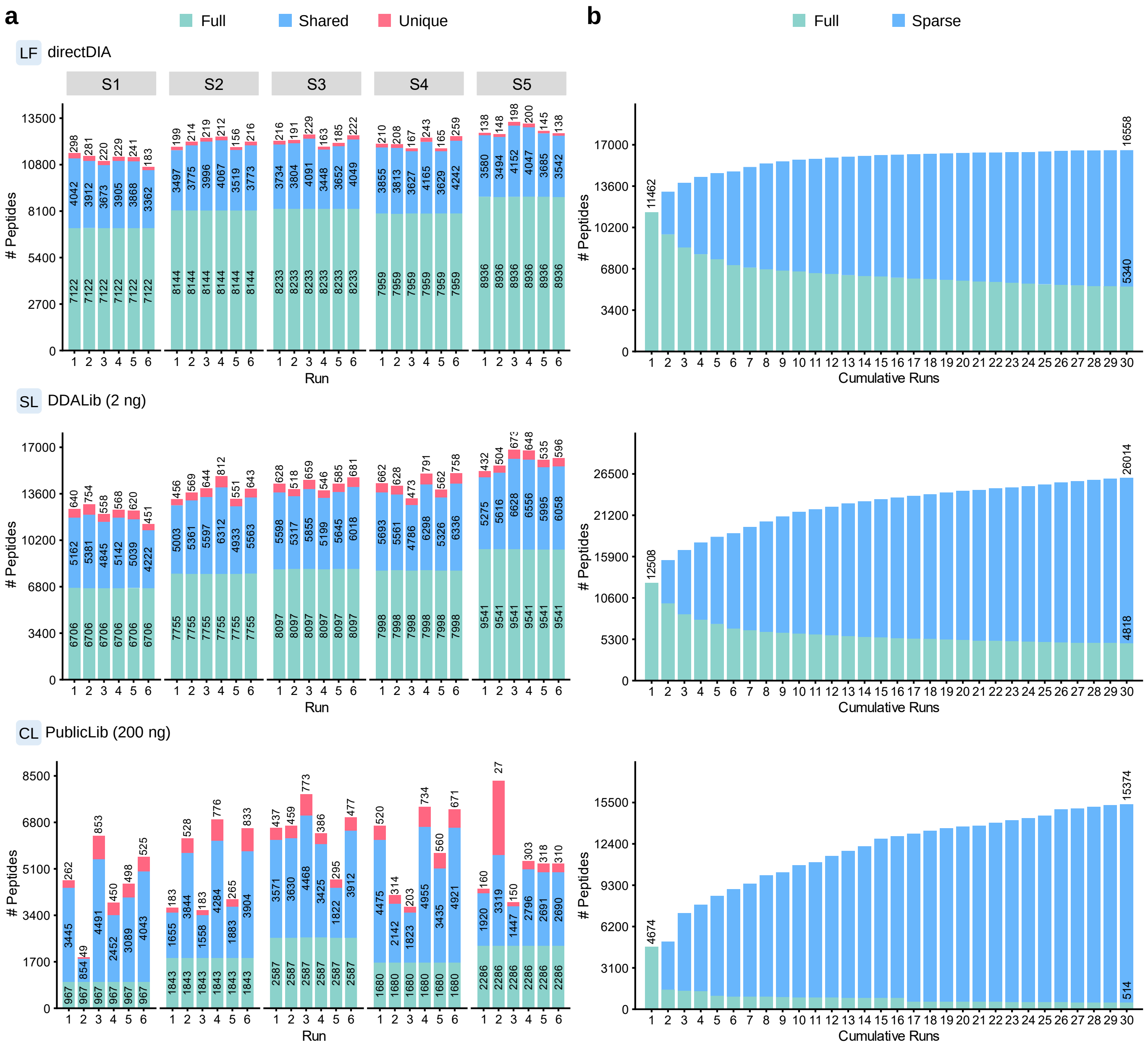

Figure SD1-13. Comparison of detection capabilities by different searching strategies using Spectronaut at the peptide level.
a Numbers of quantified peptides per run. Full peptides (in green) represent those quantified in all the runs of a sample; shared peptides (in blue) represent those quantified in 2 bot not all runs of a sample; unique peptides (in red) represent those quantified in only 1 run. **b** Numbers of cumulative peptides from run 1 to 30 (in the order of samples S1–S5 and replicates 1–6 for each sample). Full peptides (in green) represent those shared in the cumulative runs; sparse peptides (in blue) represent those quantified in at least 1 run in the cumulative runs.

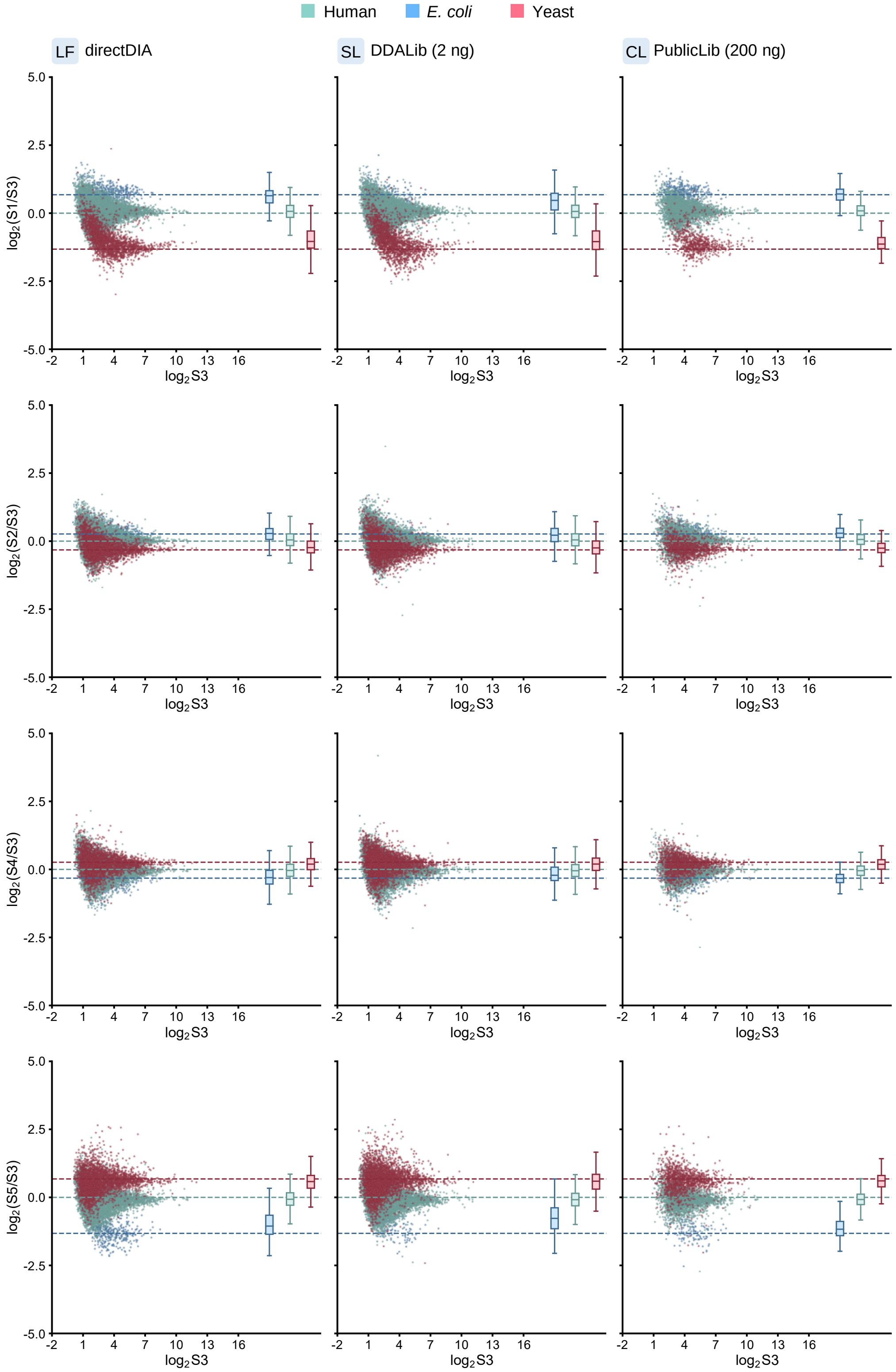

Figure SD1-14. Comparison of quantitative accuracies by different searching strategies using Spectronaut at the peptide level.

Measured fold change (FC) values of peptide quantities using sample S3 as reference. FC values were calculated only for peptides quantified in at least 3 runs for each sample of the comparison. The boxes mark the first and third quantile and the lines inside the boxes mark the median; the whiskers extend from the box to the farthest point lying within 1.5 times the inter-quartile range. The theoretical ratios are highlighted as dashed lines. Colors indicate peptides from human (in green), yeast (in red), and *E. coli* (in blue).

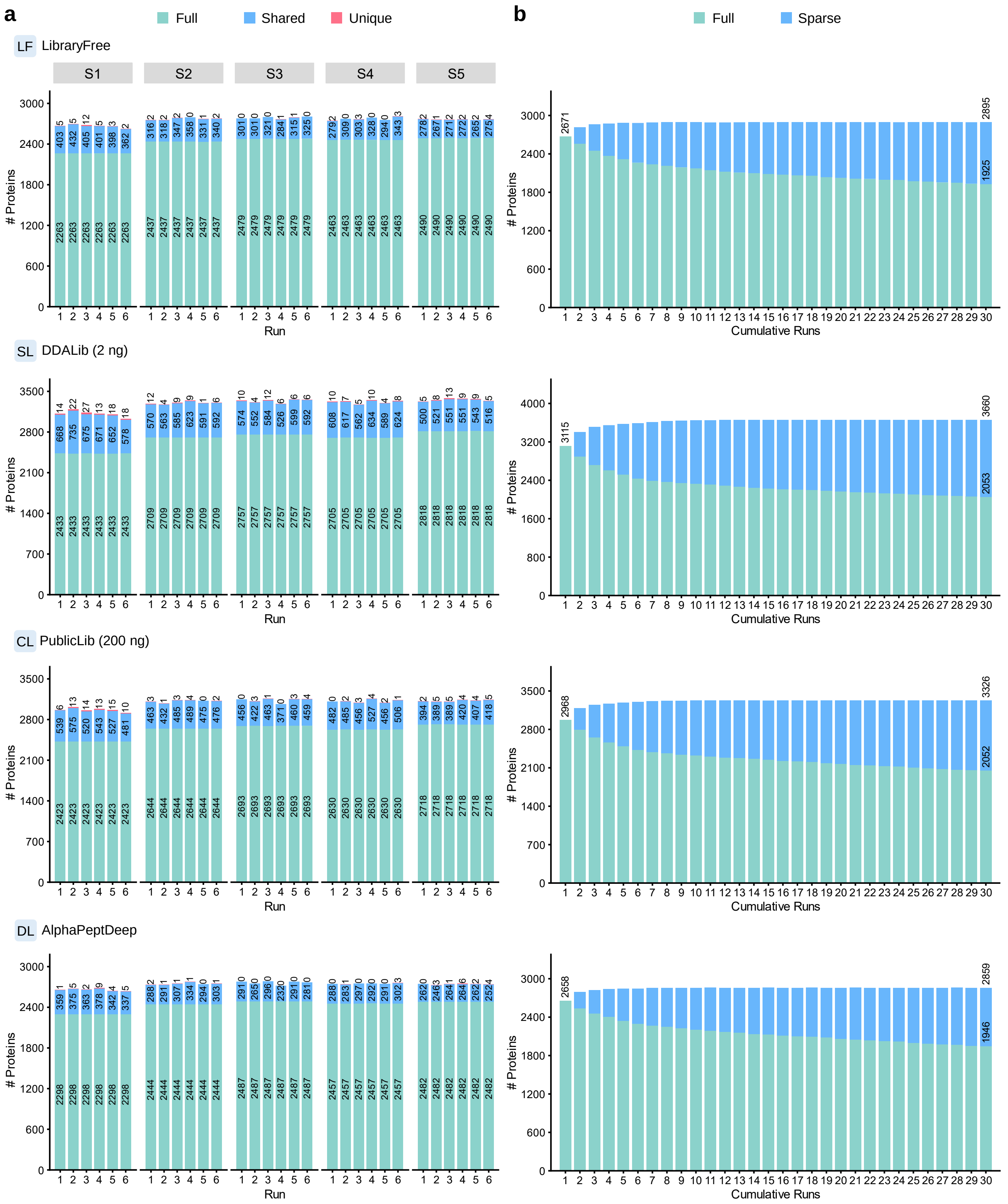

Figure SD1-15. Comparison of detection capabilities by different searching strategies using PEAKS at the protein level.
a Numbers of quantified proteins per run. Full proteins (in green) represent those quantified in all the runs of a sample; shared proteins (in blue) represent those quantified in 2 bot not all runs of a sample; unique proteins (in red) represent those quantified in only 1 run. **b** Numbers of cumulative proteins from run 1 to 30 (in the order of samples S1–S5 and replicates 1–6 for each sample). Full proteins (in green) represent those shared in the cumulative runs; sparse proteins (in blue) represent those quantified in at least 1 run in the cumulative runs.

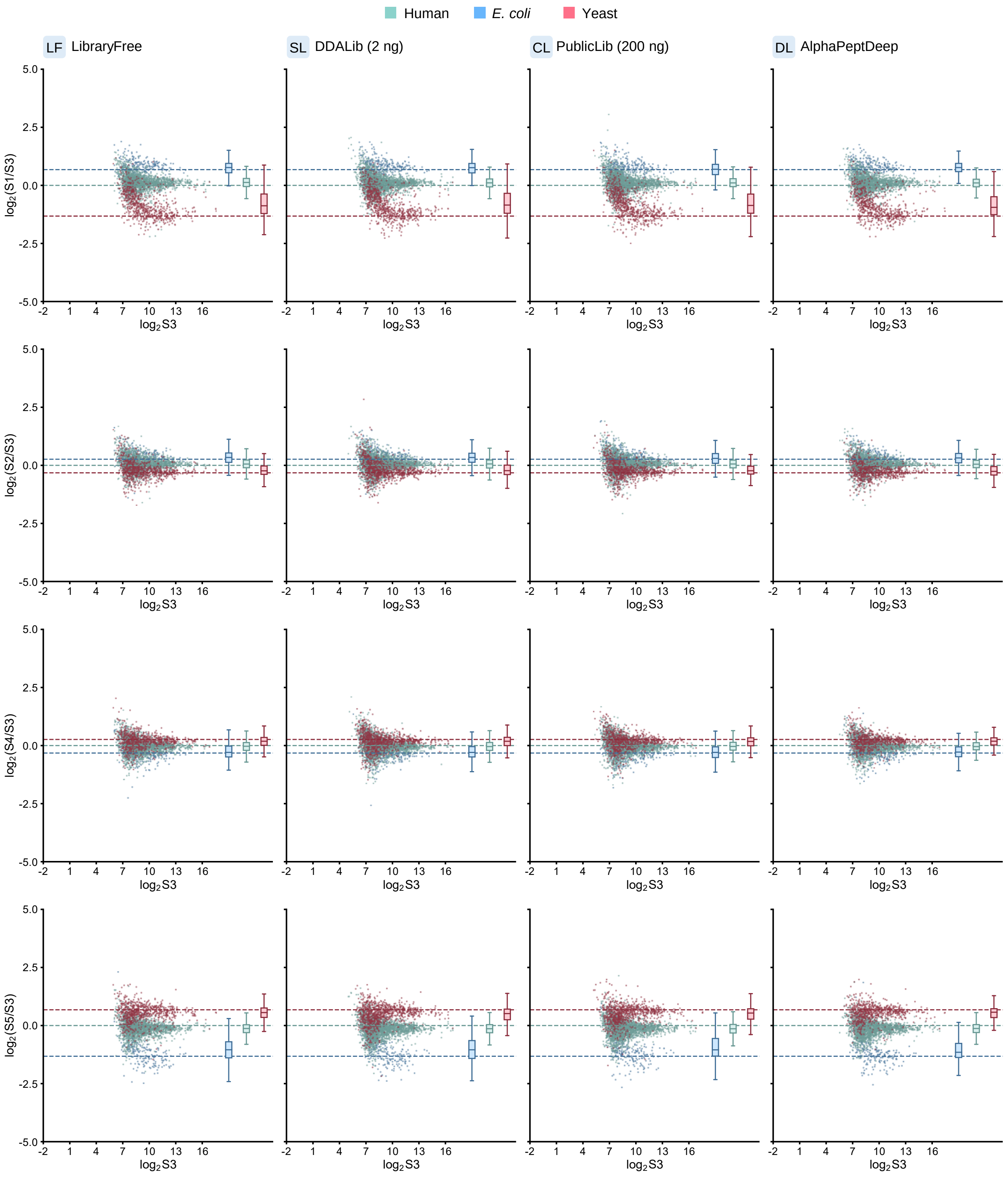

Figure SD1-16. Comparison of quantitative accuracies by different searching strategies using PEAKS at the protein level.

Measured fold change (FC) values of protein quantities using sample S3 as reference. FC values were calculated only for proteins quantified in at least 3 runs for each sample of the comparison. The boxes mark the first and third quantile and the lines inside the boxes mark the median; the whiskers extend from the box to the farthest point lying within 1.5 times the inter-quartile range. The theoretical ratios are highlighted as dashed lines. Colors indicate proteins from human (in green), yeast (in red), and *E. coli* (in blue).

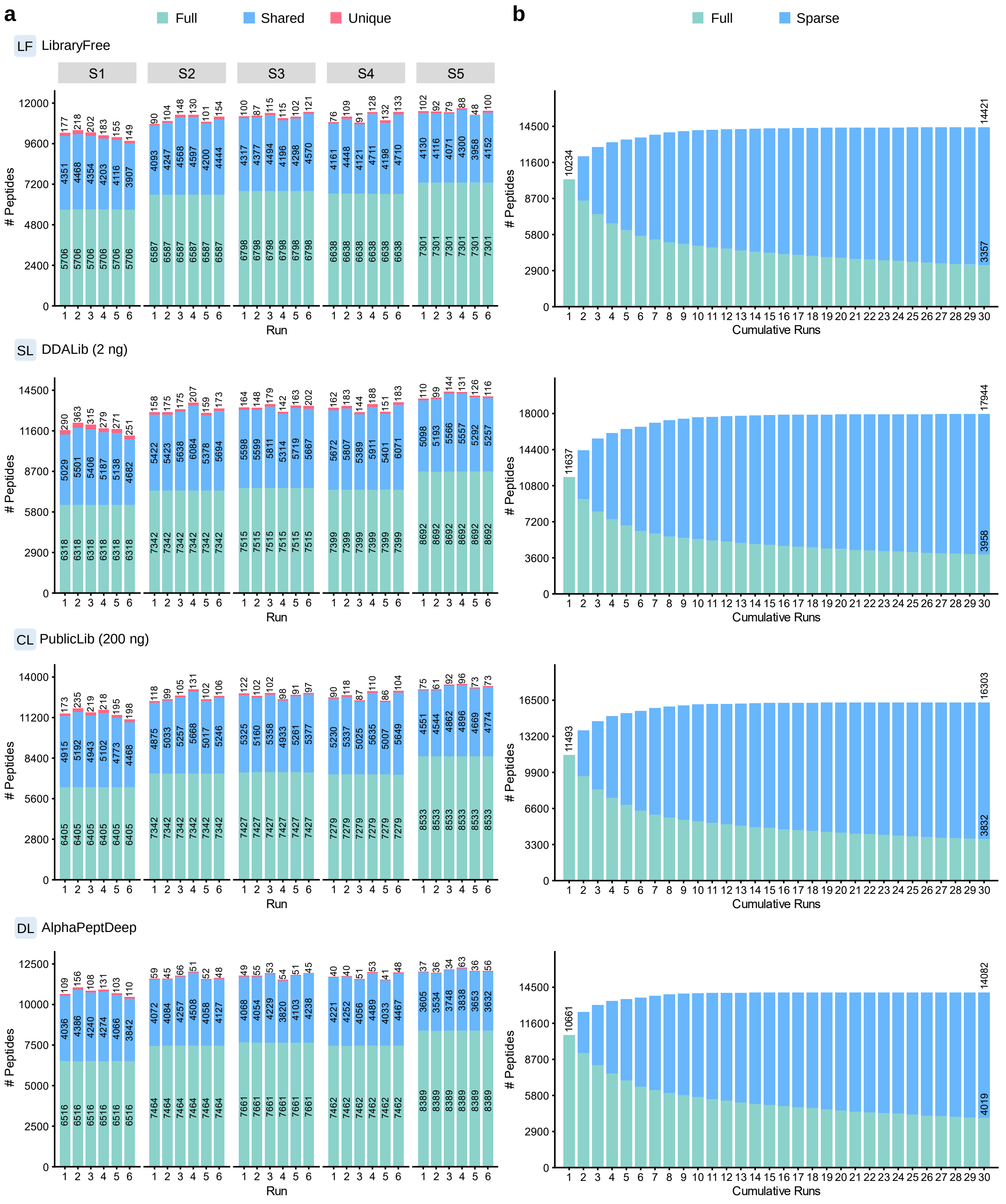

Figure SD1-17. Comparison of detection capabilities by different searching strategies using PEAKS at the peptide level.

a Numbers of quantified peptides per run. Full peptides (in green) represent those quantified in all the runs of a sample; shared peptides (in blue) represent those quantified in 2 bot not all runs of a sample; unique peptides (in red) represent those quantified in only 1 run. **b** Numbers of cumulative peptides from run 1 to 30 (in the order of samples S1–S5 and replicates 1–6 for each sample). Full peptides (in green) represent those shared in the cumulative runs; sparse peptides (in blue) represent those quantified in at least 1 run in the cumulative runs.

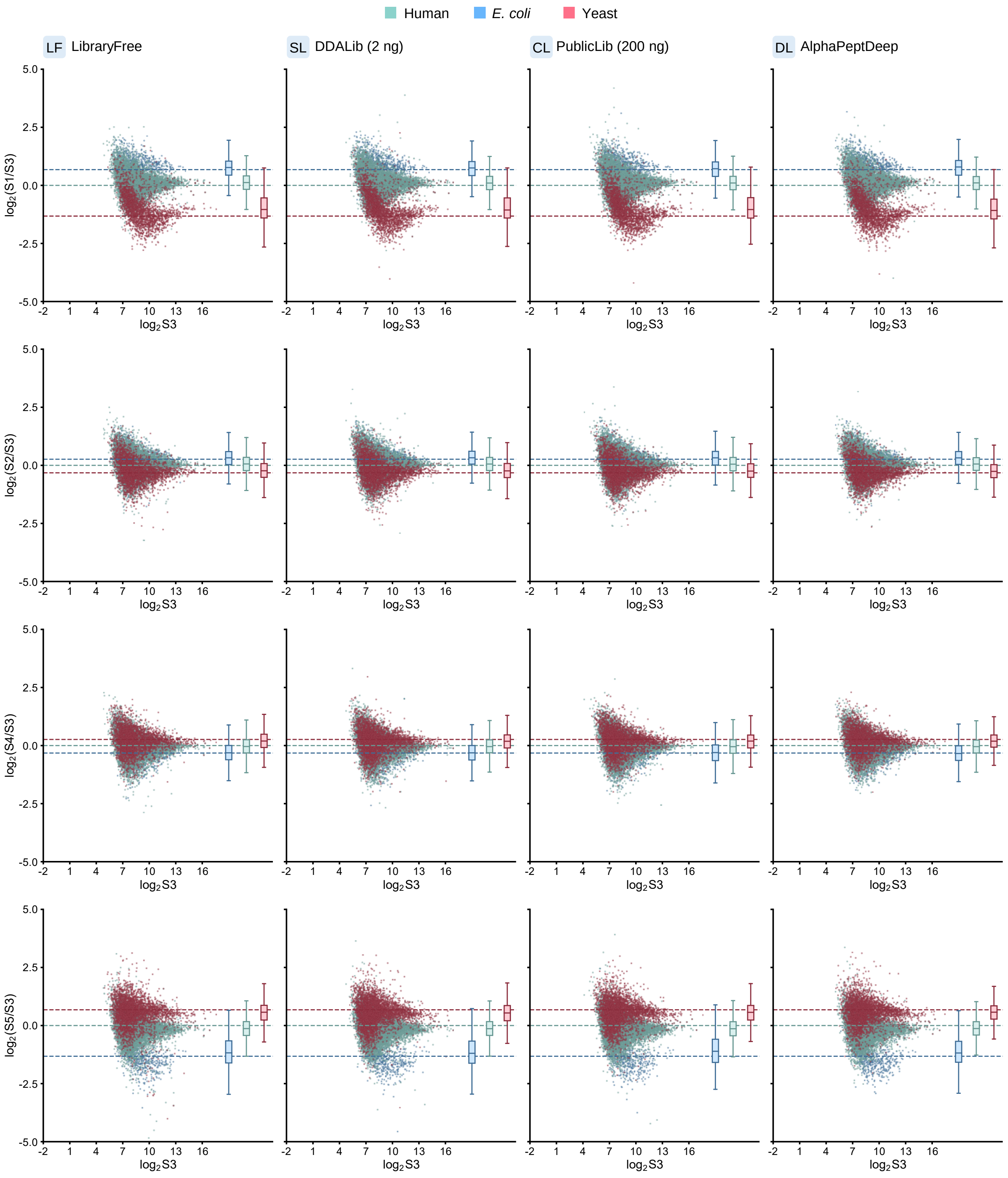

Figure SD1-18. Comparison of quantitative accuracies by different searching strategies using PEAKS at the peptide level.

Measured fold change (FC) values of peptide quantities using sample S3 as reference. FC values were calculated only for peptides quantified in at least 3 runs for each sample of the comparison. The boxes mark the first and third quantile and the lines inside the boxes mark the median; the whiskers extend from the box to the farthest point lying within 1.5 times the inter-quartile range. The theoretical ratios are highlighted as dashed lines. Colors indicate peptides from human (in green), yeast (in red), and *E. coli* (in blue).
