# Supplementary material for: Benchmarking informatics workflows for data-independent acquisition single-cell proteomics
Source: Nat Commun. 2025 Nov 21;16:10276. doi: 10.1038/s41467-025-65174-4 (PMC12639053; doi:10.1038/s41467-025-65174-4)
Supplement: Supplementary file 4 — Supplementary Data 2 [file 41467_2025_65174_MOESM4_ESM.zip › FigSD2-[1-8] Inter-software Comparison.pdf]

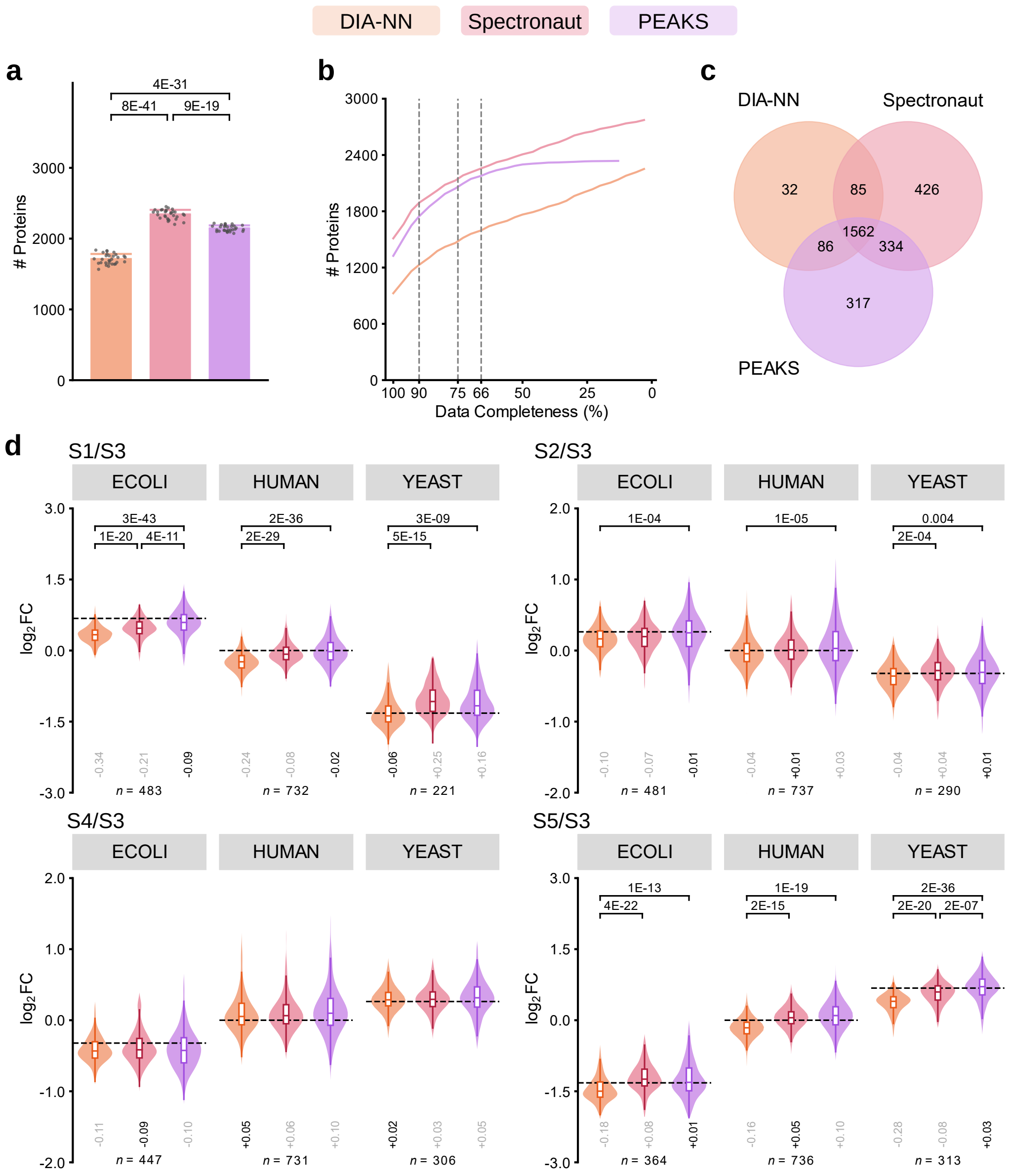

Figure SD2-1. Performance comparison of different DIA data analysis software tools using the library free strategy at the protein level.

a Numbers of quantified proteins per run. The bars indicate the mean values and the error bars indicate the standard deviations. Significant differences (t-test p-value < 0.05, two-sided, no adjustments) are indicated. **b** Numbers of proteins quantified in at least specified percentages (data completeness) of runs. **c** Overlap of the proteins quantified in at least 50% runs. **d** Measured fold change (FC) values of protein quantities using sample S3 as reference. FC values were calculated only for proteins quantified in at least 3 runs for each sample of the comparison. Numbers (*n*) of proteins are indicated for each species. The boxes mark the first and third quantile and the lines inside the boxes mark the median; the whiskers extend from the box to the farthest point lying within 1.5 times the inter-quartile range; outliers are not shown. The theoretical ratios are highlighted as dashed lines. Differences between the measured median FC values and theoretical values are indicated, among which the smallest ones are darkened. Significant differences (t-test p-value < 0.05 and Cohen's |*d*| > 0.2, two-sided, no adjustments) are indicated.

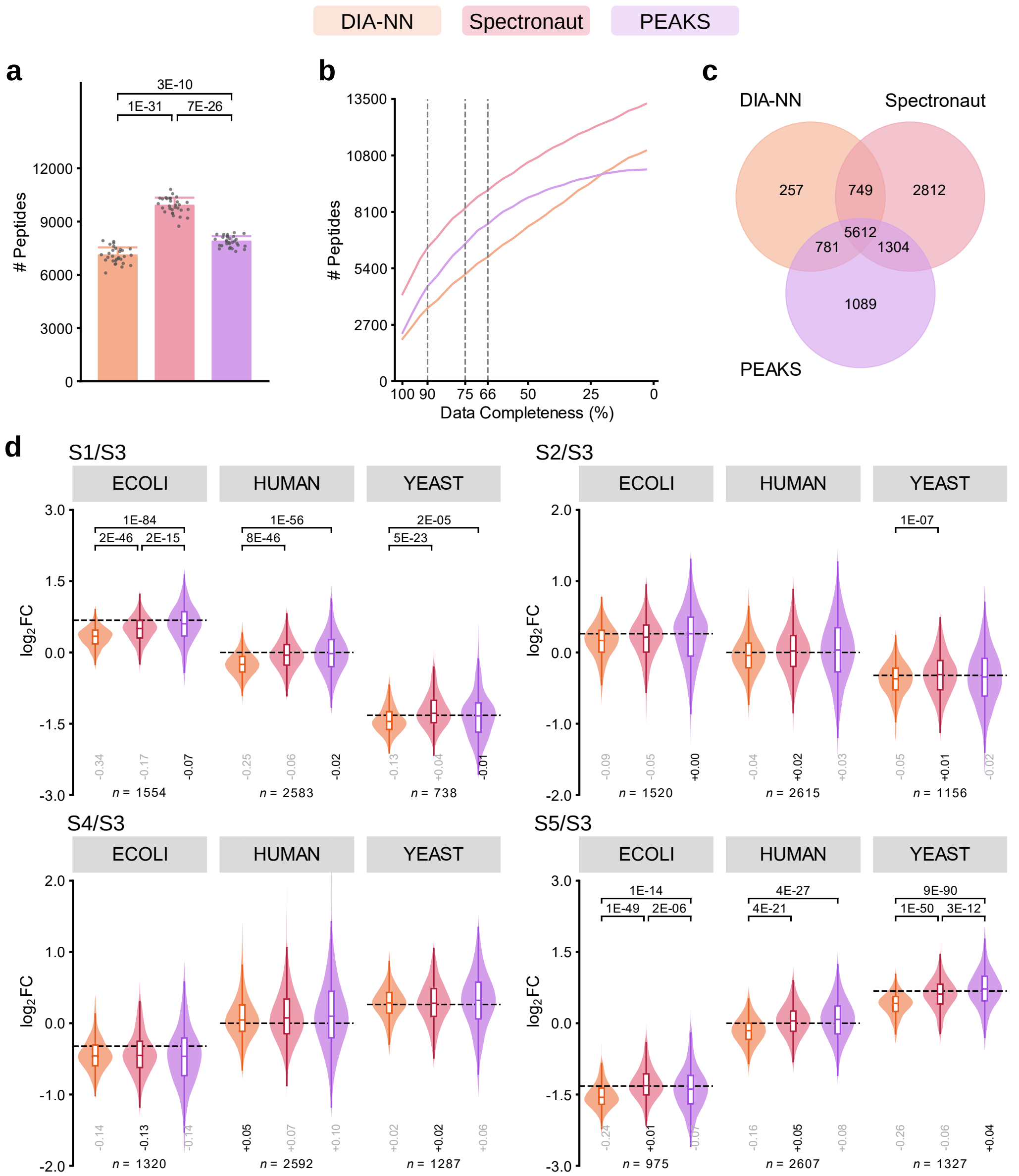

Figure SD2-2. Performance comparison of different DIA data analysis software tools using the library free strategy at the peptide level.

a Numbers of quantified peptides per run. The bars indicate the mean values and the error bars indicate the standard deviations. Significant differences (t-test p-value < 0.05, two-sided, no adjustments) are indicated. **b** Numbers of peptides quantified in at least specified percentages (data completeness) of runs. **c** Overlap of the peptides quantified in at least 50% runs. **d** Measured fold change (FC) values of peptide quantities using sample S3 as reference. FC values were calculated only for peptides quantified in at least 3 runs for each sample of the comparison. Numbers (*n*) of peptides are indicated for each species. The boxes mark the first and third quantile and the lines inside the boxes mark the median; the whiskers extend from the box to the farthest point lying within 1.5 times the inter-quartile range; outliers are not shown. The theoretical ratios are highlighted as dashed lines. Differences between the measured median FC values and theoretical values are indicated, among which the smallest ones are darkened. Significant differences (t-test p-value < 0.05 and Cohen's |*d*| > 0.2, two-sided, no adjustments) are indicated.

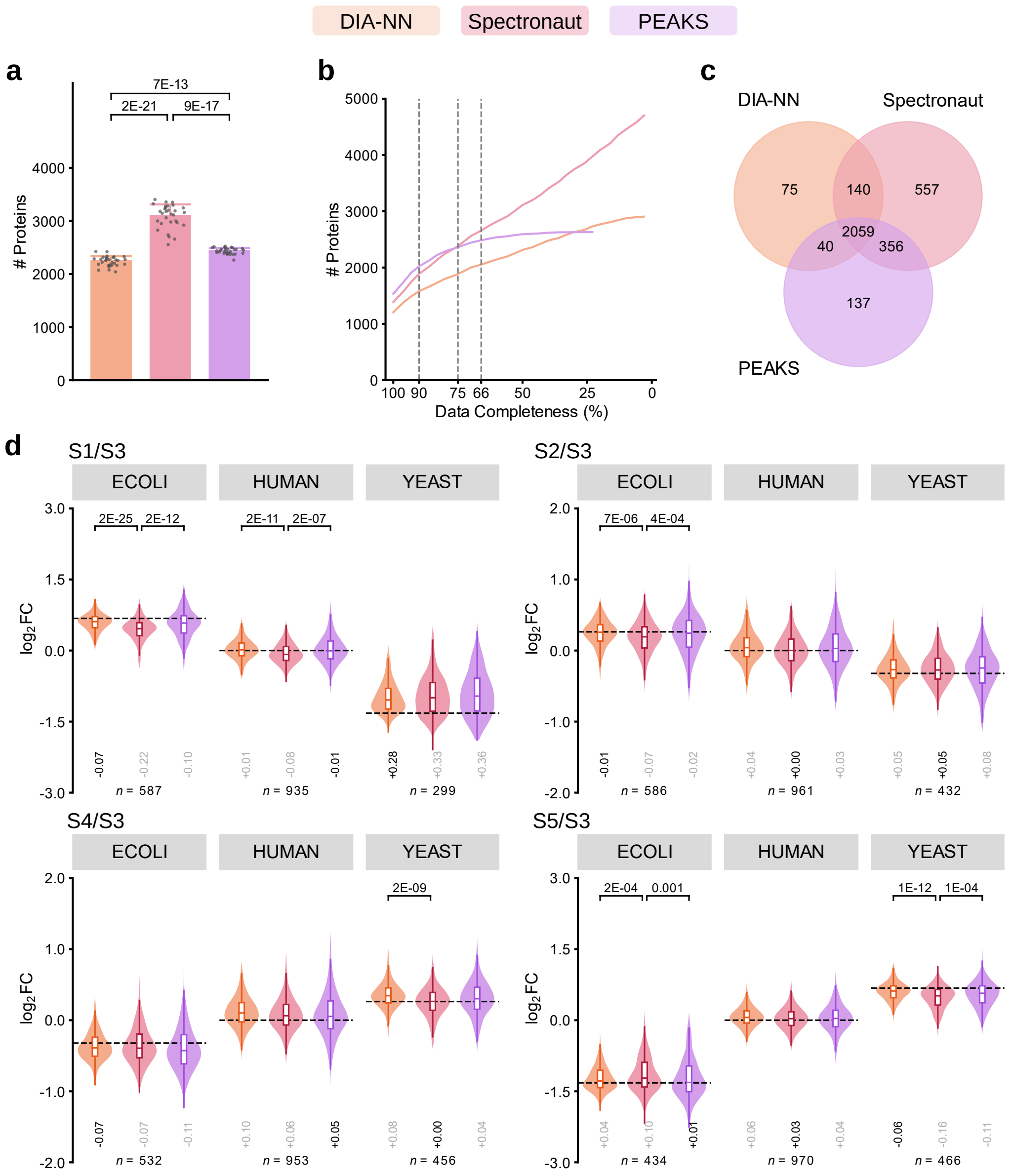

Figure SD2-3. Performance comparison of different DIA data analysis software tools using the DDALib strategy at the protein level.

a Numbers of quantified proteins per run. The bars indicate the mean values and the error bars indicate the standard deviations. Significant differences (t-test p-value < 0.05, two-sided, no adjustments) are indicated. **b** Numbers of proteins quantified in at least specified percentages (data completeness) of runs. **c** Overlap of the proteins quantified in at least 50% runs. **d** Measured fold change (FC) values of protein quantities using sample S3 as reference. FC values were calculated only for proteins quantified in at least 3 runs for each sample of the comparison. Numbers (*n*) of proteins are indicated for each species. The boxes mark the first and third quantile and the lines inside the boxes mark the median; the whiskers extend from the box to the farthest point lying within 1.5 times the inter-quartile range; outliers are not shown. The theoretical ratios are highlighted as dashed lines. Differences between the measured median FC values and theoretical values are indicated, among which the smallest ones are darkened. Significant differences (t-test p-value < 0.05 and Cohen's |*d*| > 0.2, two-sided, no adjustments) are indicated.

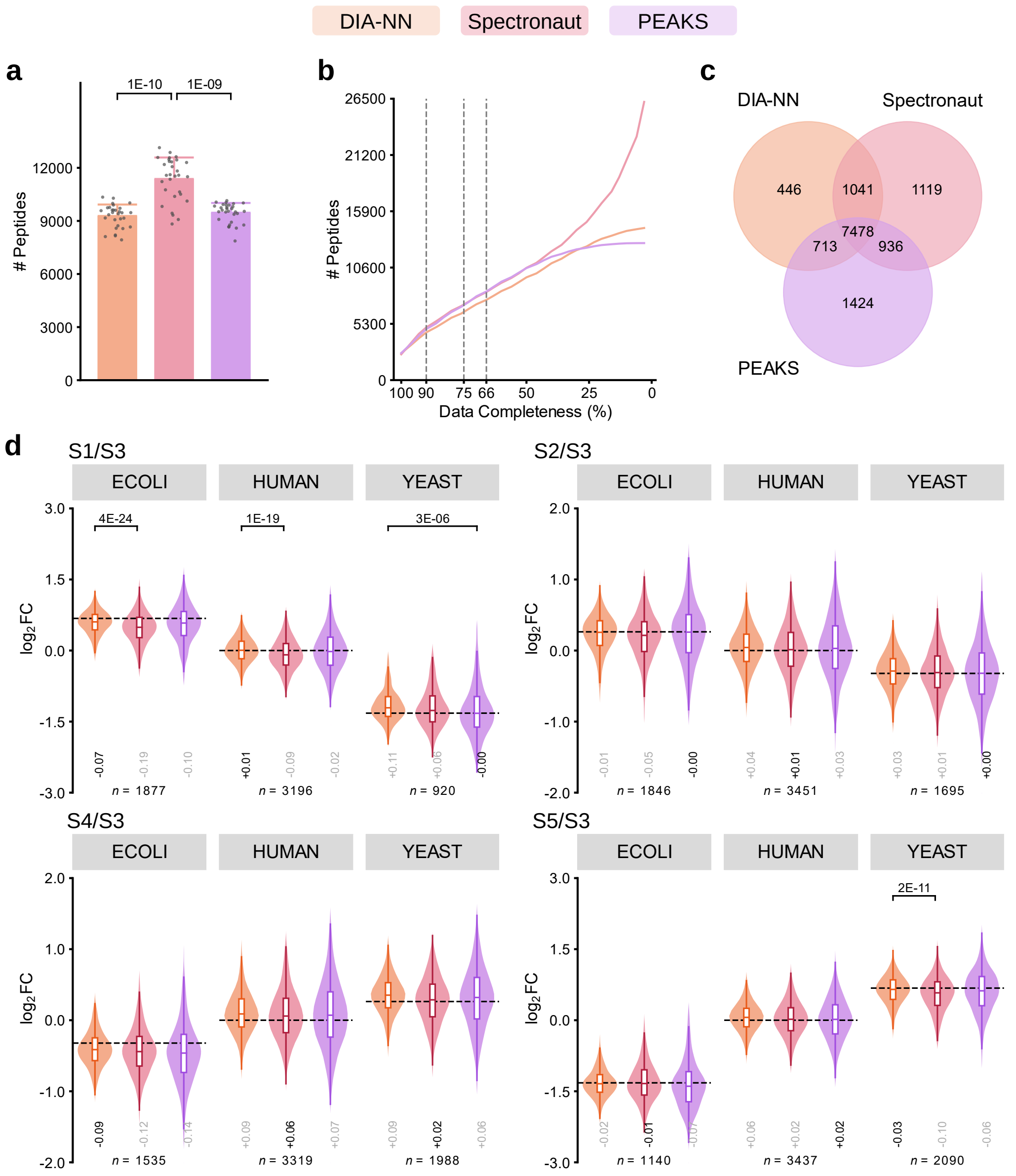

Figure SD2-4. Performance comparison of different DIA data analysis software tools using the DDALib strategy at the peptide level.

a Numbers of quantified peptides per run. The bars indicate the mean values and the error bars indicate the standard deviations. Significant differences (t-test p-value < 0.05, two-sided, no adjustments) are indicated. **b** Numbers of peptides quantified in at least specified percentages (data completeness) of runs. **c** Overlap of the peptides quantified in at least 50% runs. **d** Measured fold change (FC) values of peptide quantities using sample S3 as reference. FC values were calculated only for peptides quantified in at least 3 runs for each sample of the comparison. Numbers (*n*) of peptides are indicated for each species. The boxes mark the first and third quantile and the lines inside the boxes mark the median; the whiskers extend from the box to the farthest point lying within 1.5 times the inter-quartile range; outliers are not shown. The theoretical ratios are highlighted as dashed lines. Differences between the measured median FC values and theoretical values are indicated, among which the smallest ones are darkened. Significant differences (t-test p-value < 0.05 and Cohen's |*d*| > 0.2, two-sided, no adjustments) are indicated.

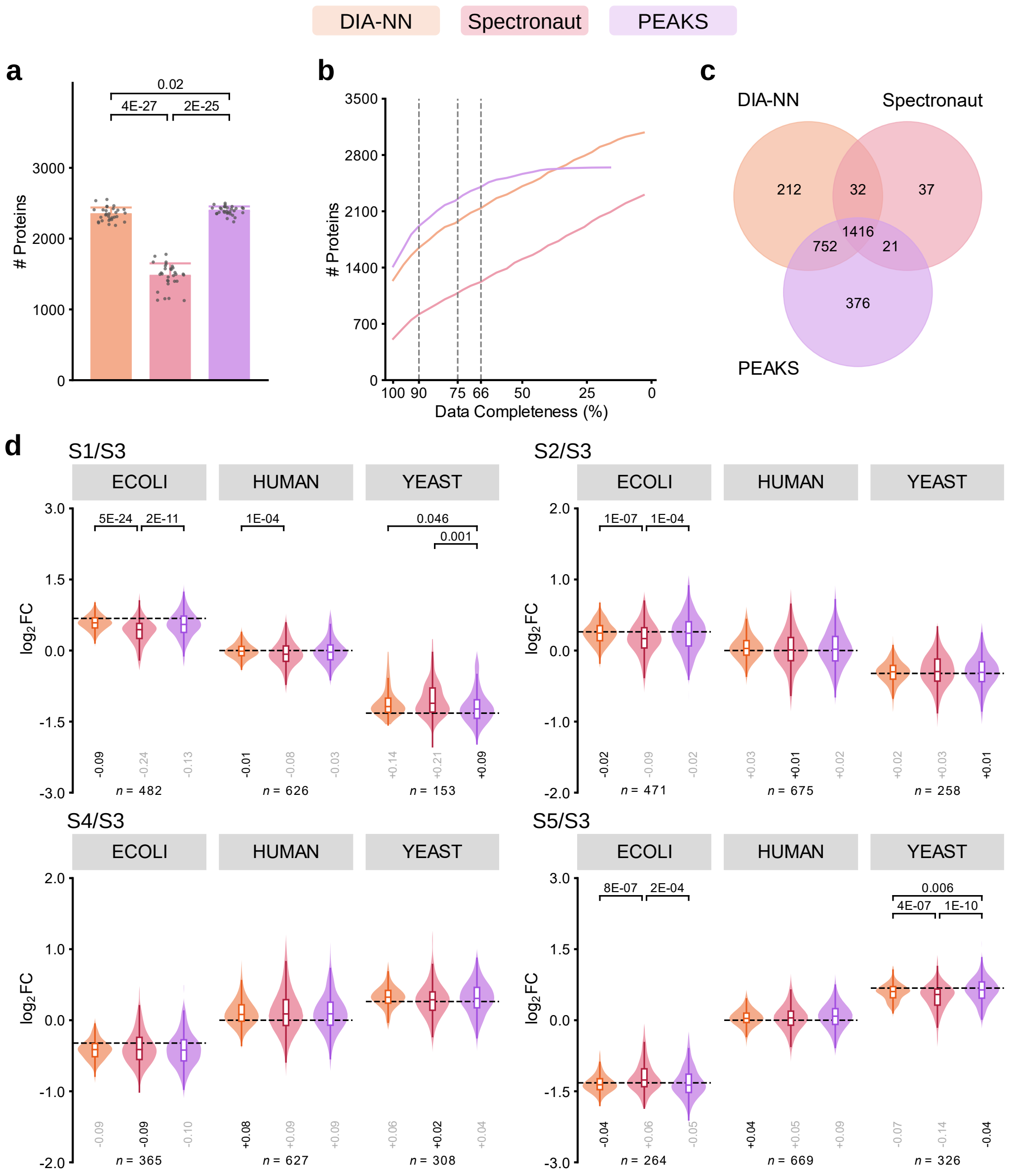

Figure SD2-5. Performance comparison of different DIA data analysis software tools using the PublicLib strategy at the protein level.

a Numbers of quantified proteins per run. The bars indicate the mean values and the error bars indicate the standard deviations. Significant differences (t-test p-value < 0.05, two-sided, no adjustments) are indicated. **b** Numbers of proteins quantified in at least specified percentages (data completeness) of runs. **c** Overlap of the proteins quantified in at least 50% runs. **d** Measured fold change (FC) values of protein quantities using sample S3 as reference. FC values were calculated only for proteins quantified in at least 3 runs for each sample of the comparison. Numbers (*n*) of proteins are indicated for each species. The boxes mark the first and third quantile and the lines inside the boxes mark the median; the whiskers extend from the box to the farthest point lying within 1.5 times the inter-quartile range; outliers are not shown. The theoretical ratios are highlighted as dashed lines. Differences between the measured median FC values and theoretical values are indicated, among which the smallest ones are darkened. Significant differences (t-test p-value < 0.05 and Cohen's |*d*| > 0.2, two-sided, no adjustments) are indicated.

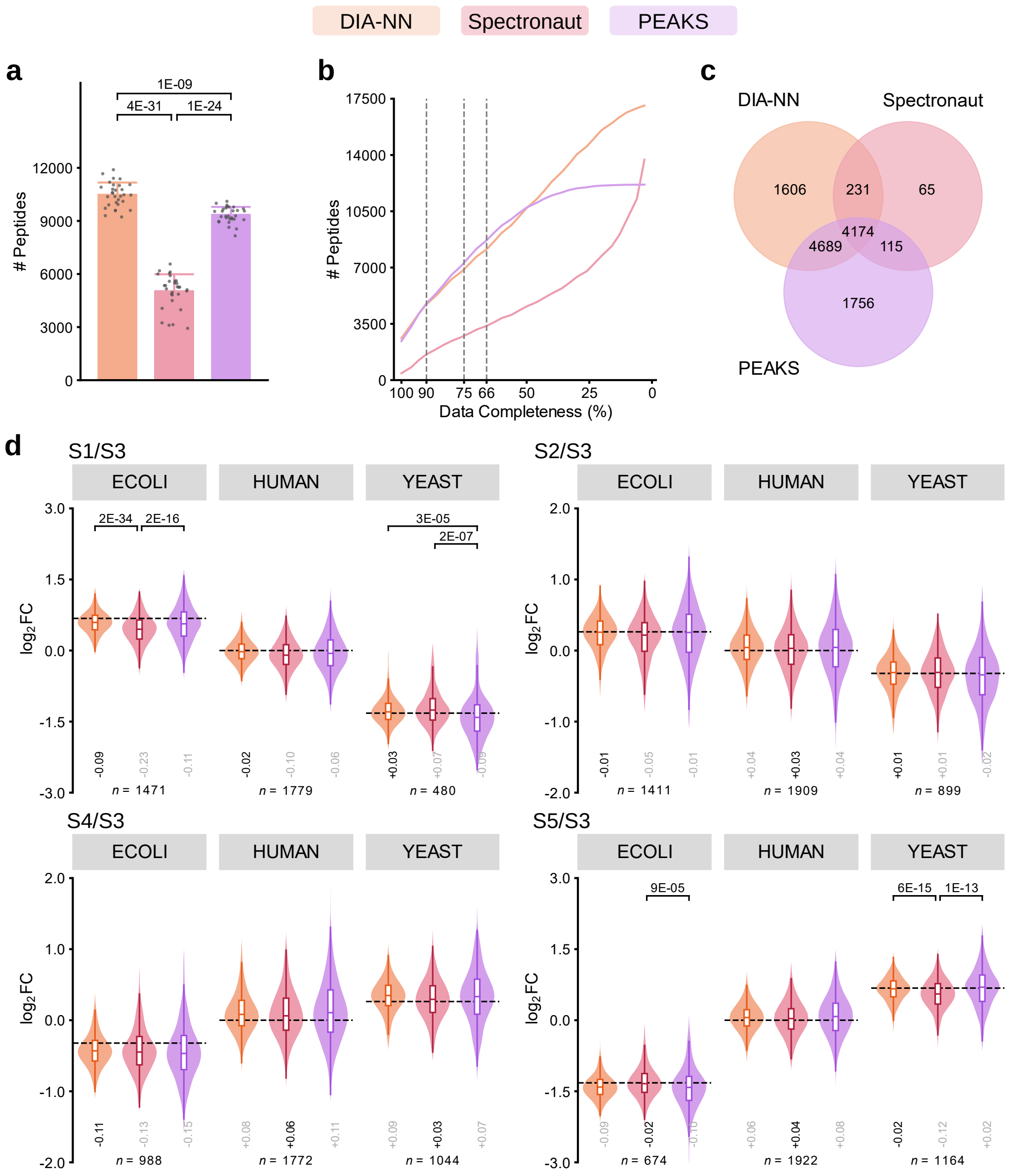

Figure SD2-6. Performance comparison of different DIA data analysis software tools using the PublicLib strategy at the peptide level.

a Numbers of quantified peptides per run. The bars indicate the mean values and the error bars indicate the standard deviations. Significant differences (t-test p-value < 0.05, two-sided, no adjustments) are indicated. **b** Numbers of peptides quantified in at least specified percentages (data completeness) of runs. **c** Overlap of the peptides quantified in at least 50% runs. **d** Measured fold change (FC) values of peptide quantities using sample S3 as reference. FC values were calculated only for peptides quantified in at least 3 runs for each sample of the comparison. Numbers (*n*) of peptides are indicated for each species. The boxes mark the first and third quantile and the lines inside the boxes mark the median; the whiskers extend from the box to the farthest point lying within 1.5 times the inter-quartile range; outliers are not shown. The theoretical ratios are highlighted as dashed lines. Differences between the measured median FC values and theoretical values are indicated, among which the smallest ones are darkened. Significant differences (t-test p-value < 0.05 and Cohen's |*d*| > 0.2, two-sided, no adjustments) are indicated.

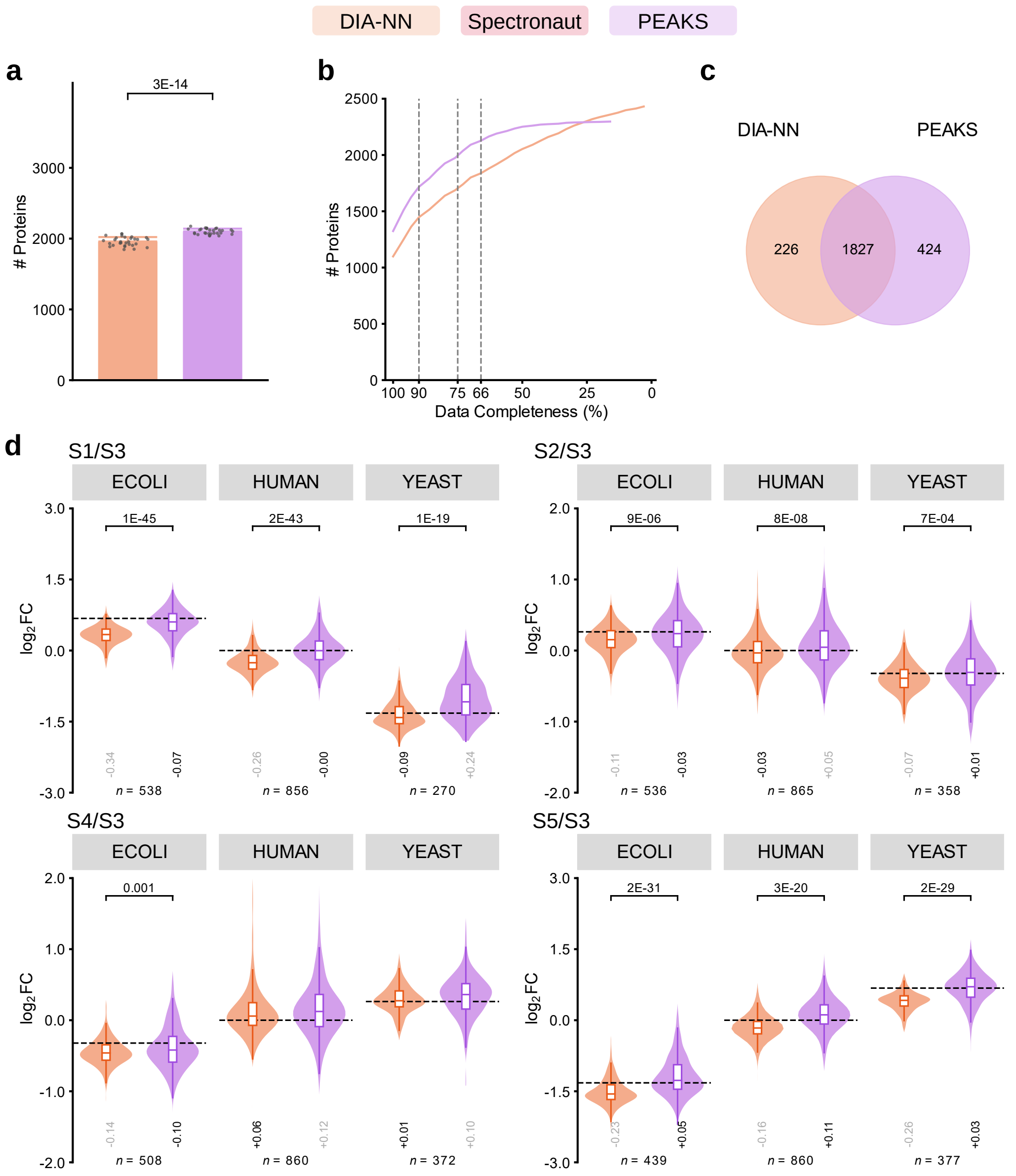

Figure SD2-7. Performance comparison of different DIA data analysis software tools using the AlphaPeptDeep strategy at the protein level.

a Numbers of quantified proteins per run. The bars indicate the mean values and the error bars indicate the standard deviations. Significant differences (t-test p-value < 0.05, two-sided, no adjustments) are indicated. **b** Numbers of proteins quantified in at least specified percentages (data completeness) of runs. **c** Overlap of the proteins quantified in at least 50% runs. **d** Measured fold change (FC) values of protein quantities using sample S3 as reference. FC values were calculated only for proteins quantified in at least 3 runs for each sample of the comparison. Numbers (*n*) of proteins are indicated for each species. The boxes mark the first and third quantile and the lines inside the boxes mark the median; the whiskers extend from the box to the farthest point lying within 1.5 times the inter-quartile range; outliers are not shown. The theoretical ratios are highlighted as dashed lines. Differences between the measured median FC values and theoretical values are indicated, among which the smallest ones are darkened. Significant differences (t-test p-value < 0.05 and Cohen's $|d| > 0.2$, two-sided, no adjustments) are indicated.

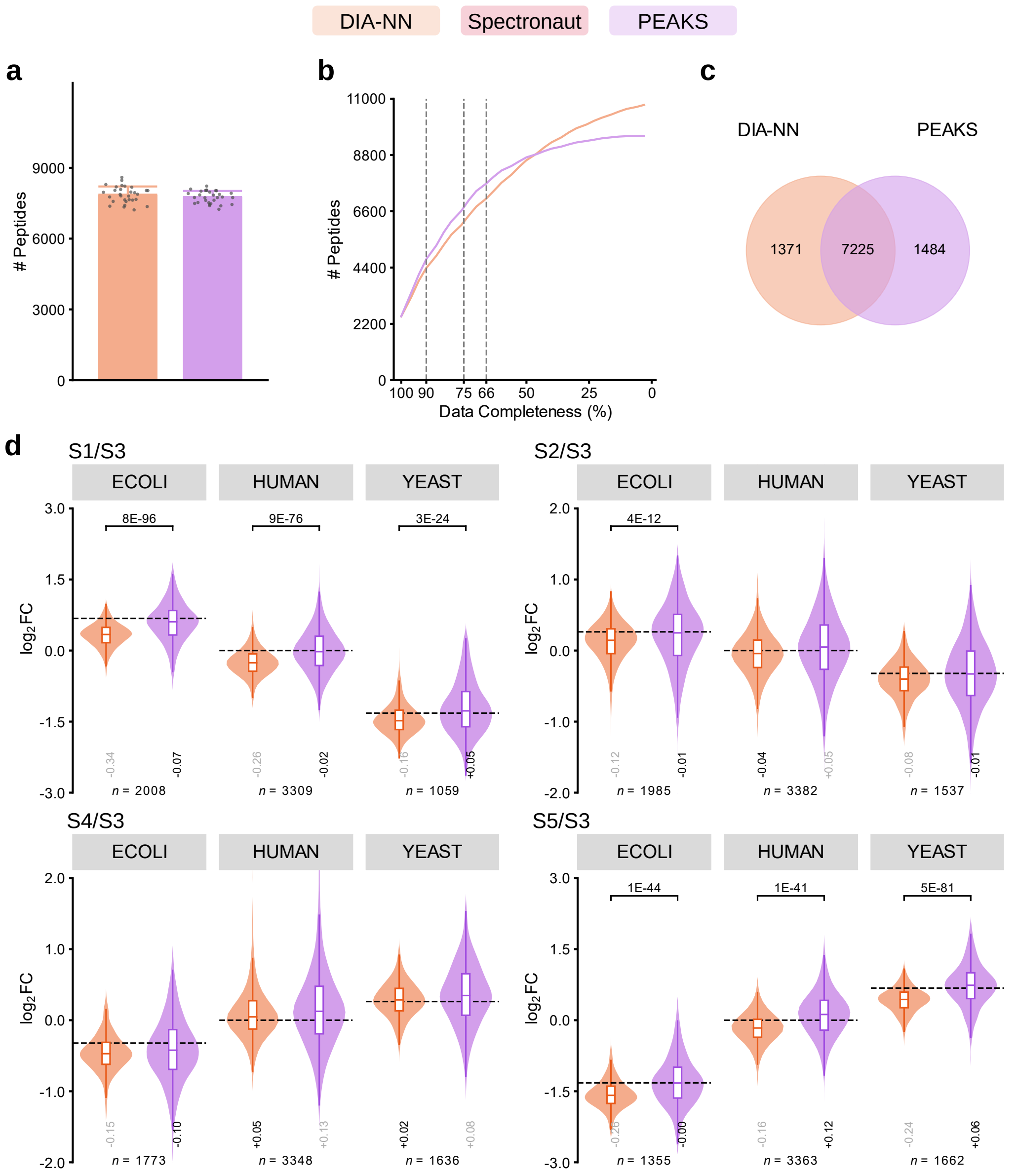

Figure SD2-8. Performance comparison of different DIA data analysis software tools using the AlphaPeptDeep strategy at the peptide level.

a Numbers of quantified peptides per run. The bars indicate the mean values and the error bars indicate the standard deviations. Significant differences (t-test p-value < 0.05, two-sided, no adjustments) are indicated. **b** Numbers of peptides quantified in at least specified percentages (data completeness) of runs. **c** Overlap of the peptides quantified in at least 50% runs. **d** Measured fold change (FC) values of peptide quantities using sample S3 as reference. FC values were calculated only for peptides quantified in at least 3 runs for each sample of the comparison. Numbers (*n*) of peptides are indicated for each species. The boxes mark the first and third quantile and the lines inside the boxes mark the median; the whiskers extend from the box to the farthest point lying within 1.5 times the inter-quartile range; outliers are not shown. The theoretical ratios are highlighted as dashed lines. Differences between the measured median FC values and theoretical values are indicated, among which the smallest ones are darkened. Significant differences (t-test p-value < 0.05 and Cohen's |*d*| > 0.2, two-sided, no adjustments) are indicated.
