# Supplementary material for: Benchmarking informatics workflows for data-independent acquisition single-cell proteomics
Source: Nat Commun. 2025 Nov 21;16:10276. doi: 10.1038/s41467-025-65174-4 (PMC12639053; doi:10.1038/s41467-025-65174-4)
Supplement: Supplementary file 4 — Supplementary Data 2 [file 41467_2025_65174_MOESM4_ESM.zip › FigSD2-[27-32] Entrapment.pdf]

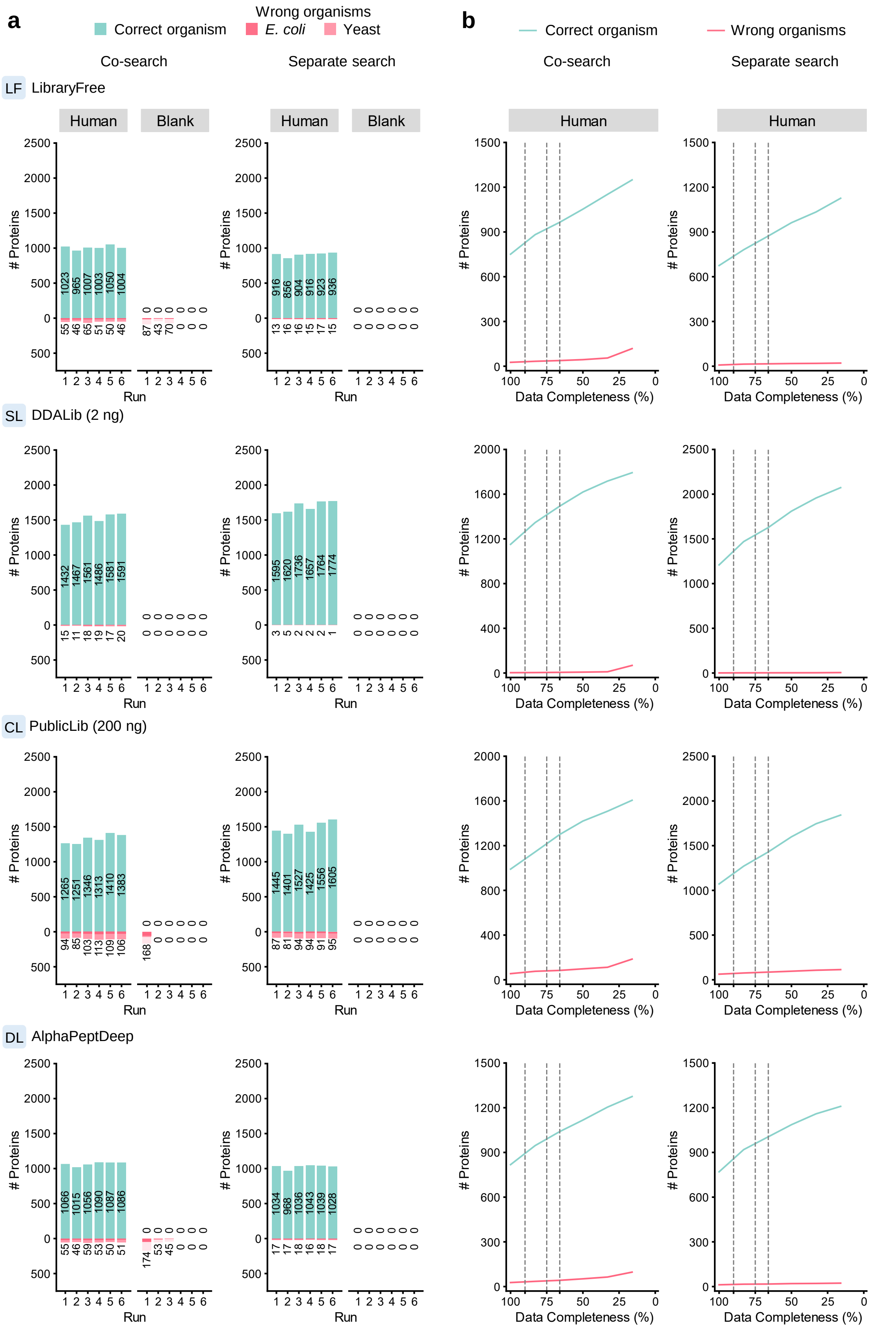

Figure SD2-27. Comparison of false positive detection by different searching strategies using DIA-NN at the protein level.

a Numbers of quantified proteins per run. For each sample, correctly detected proteins should be from the organism specific to the sample (in green), while those from other organisms (in red) are potential false positives. Results of blank injections are shown to assess potential contaminants. **b** Numbers of organism-matched and potential false positive proteins quantified in at least specified percentages (data completeness) of runs. (Continued on next page)

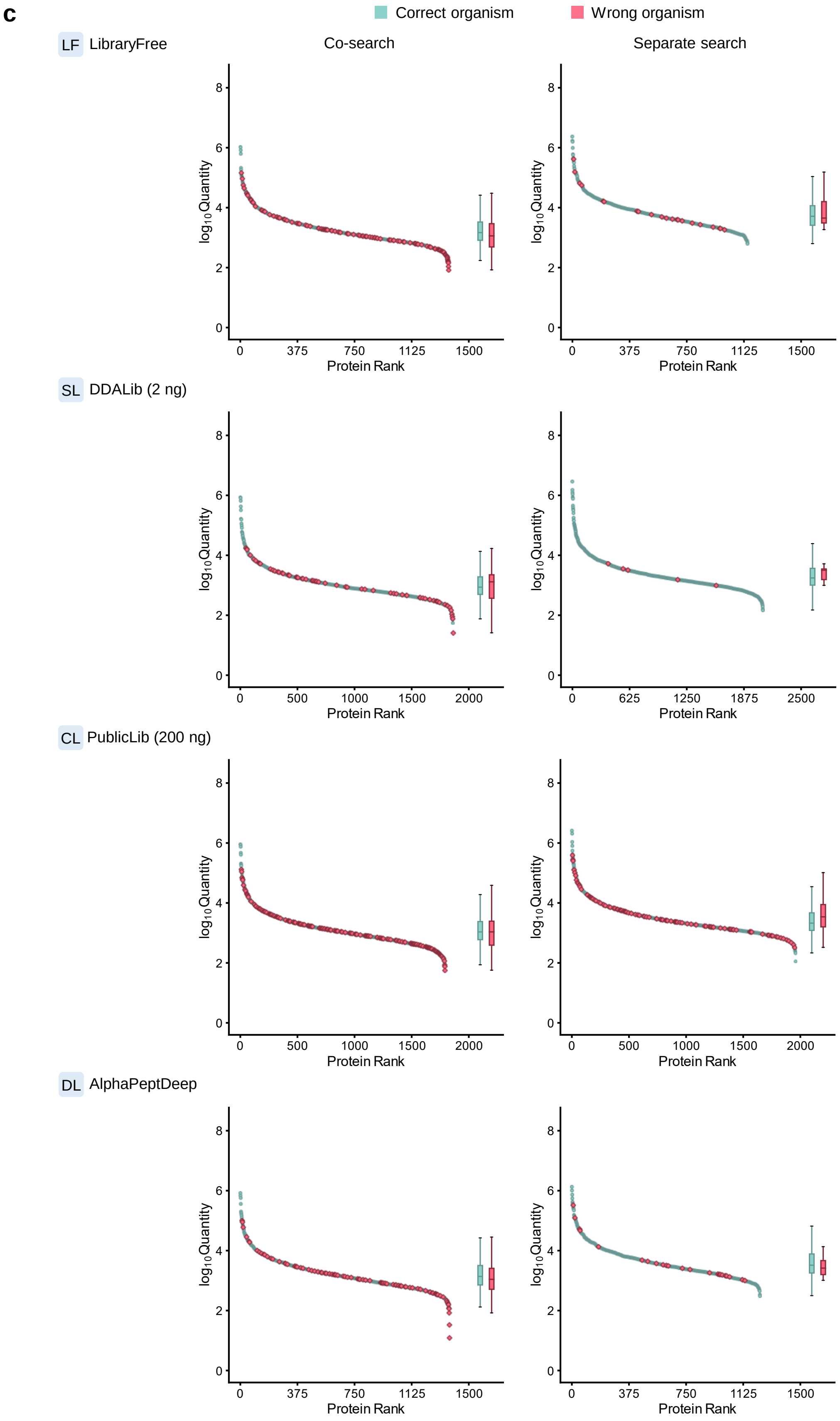

Figure SD2-27. (Continued from previous page)
c Organism-matched (in green) and potential false positive (in red) proteins ranked by their quantities (mean value across the runs for each sample). The boxes mark the first and third quantile and the lines inside the boxes mark the median; the whiskers extend from the box to the farthest point lying within 1.5 times the inter-quartile range.

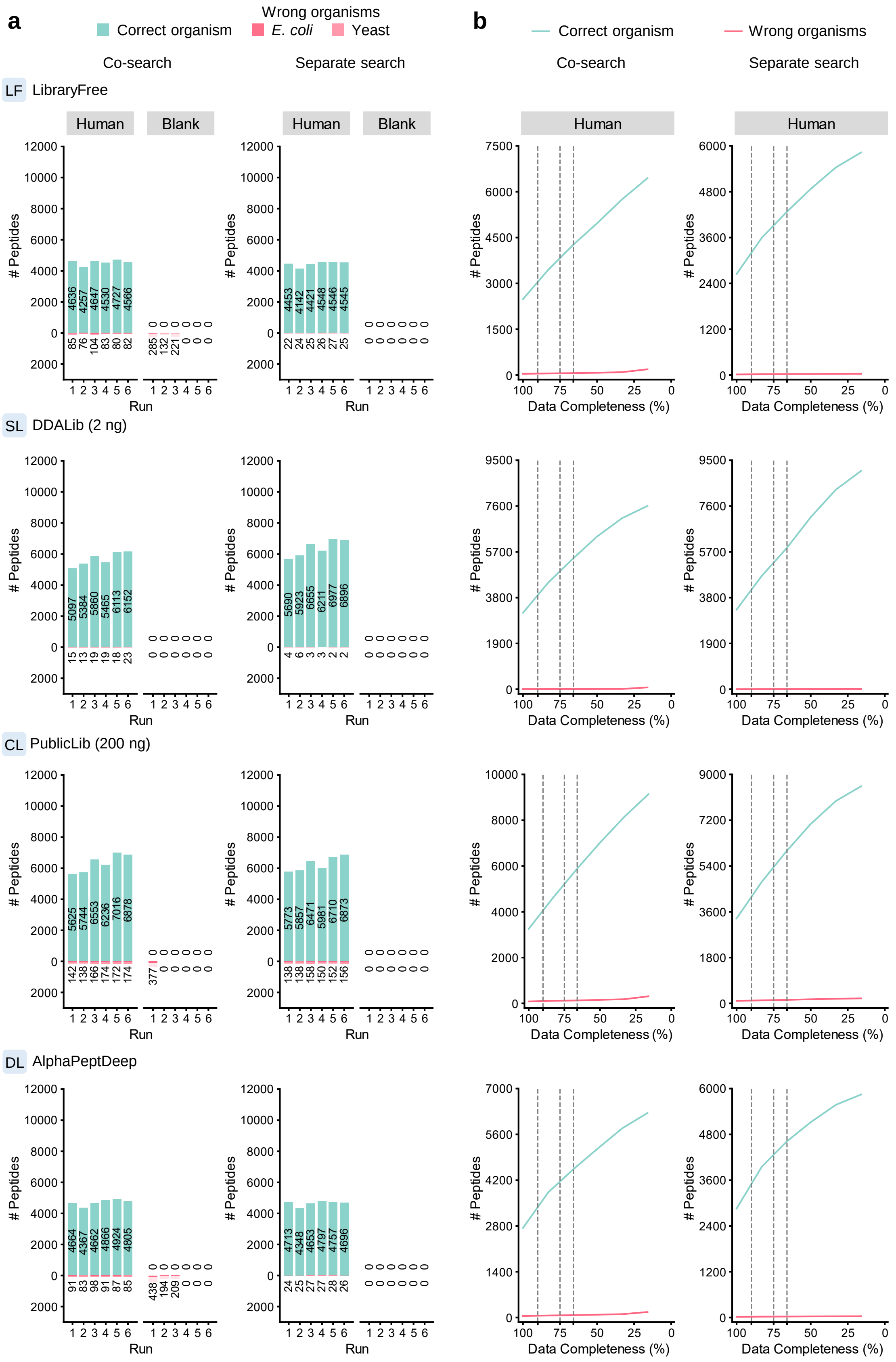

Figure SD2-28. Comparison of false positive detection by different searching strategies using DIA-NN at the peptide level.

a Numbers of quantified peptides per run. For each sample, correctly detected peptides should be from the organism specific to the sample (in green), while those from other organisms (in red) are potential false positives. Results of blank injections are shown to assess potential contaminants. **b** Numbers of organism-matched and potential false positive peptides quantified in at least specified percentages (data completeness) of runs. (Continued on next page)

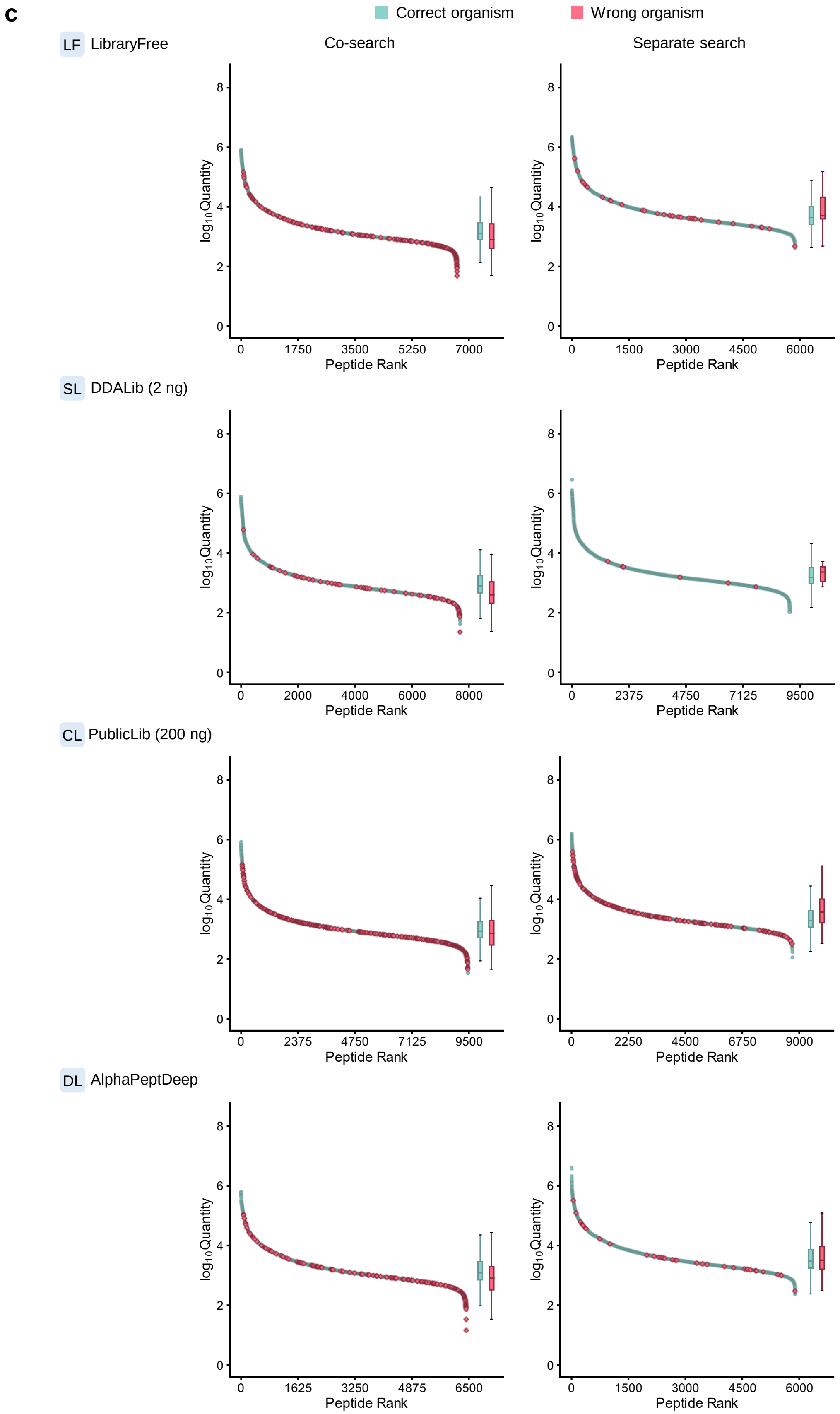

Figure SD2-28. (Continued from previous page)
c Organism-matched (in green) and potential false positive (in red) peptides ranked by their quantities (mean value across the runs for each sample). The boxes mark the first and third quantile and the lines inside the boxes mark the median; the whiskers extend from the box to the farthest point lying within 1.5 times the inter-quartile range.

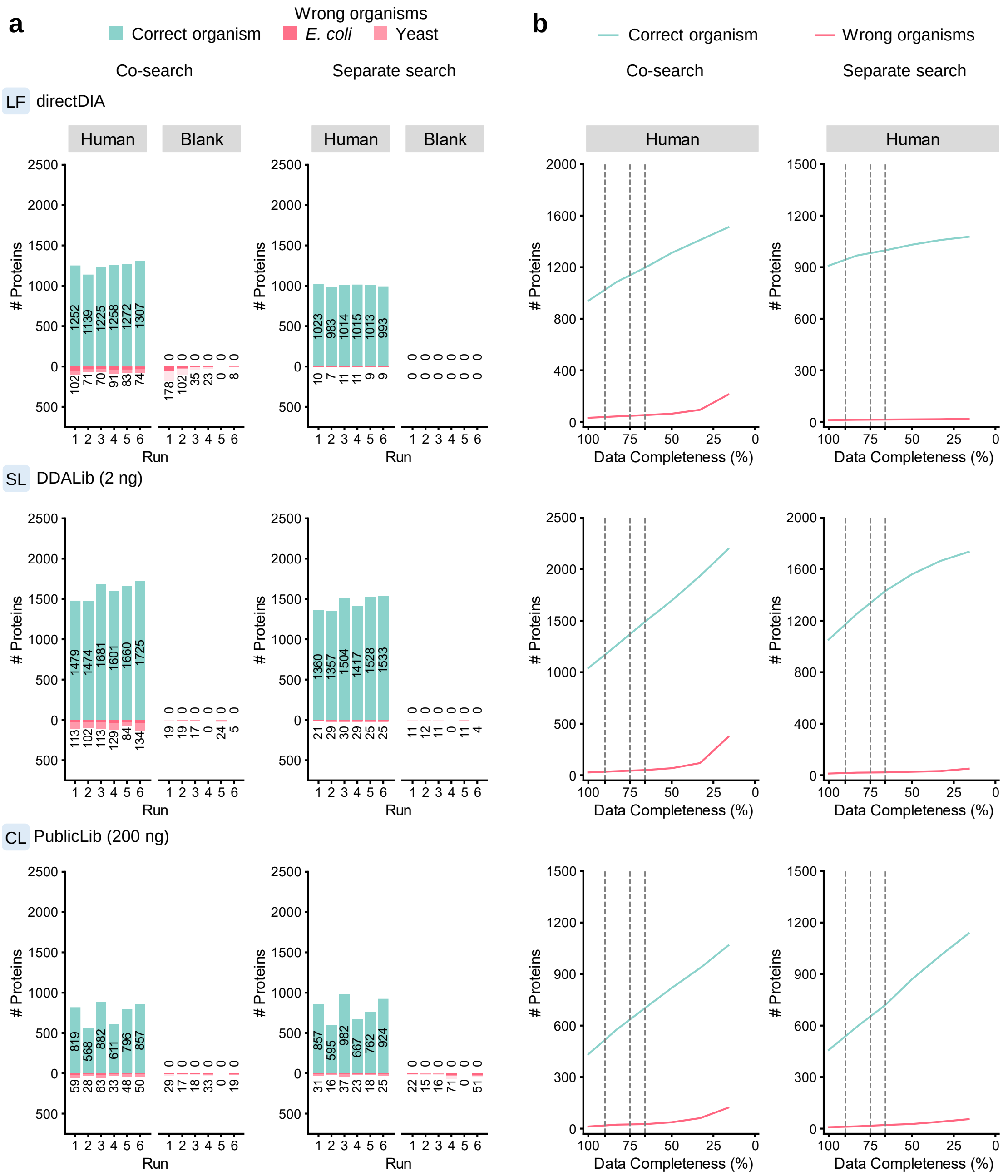

Figure SD2-29. Comparison of false positive detection by different searching strategies using Spectronaut at the protein level.

a Numbers of quantified proteins per run. For each sample, correctly detected proteins should be from the organism specific to the sample (in green), while those from other organisms (in red) are potential false positives. Results of blank injections are shown to assess potential contaminants. **b** Numbers of organism-matched and potential false positive proteins quantified in at least specified percentages (data completeness) of runs. (Continued on next page)

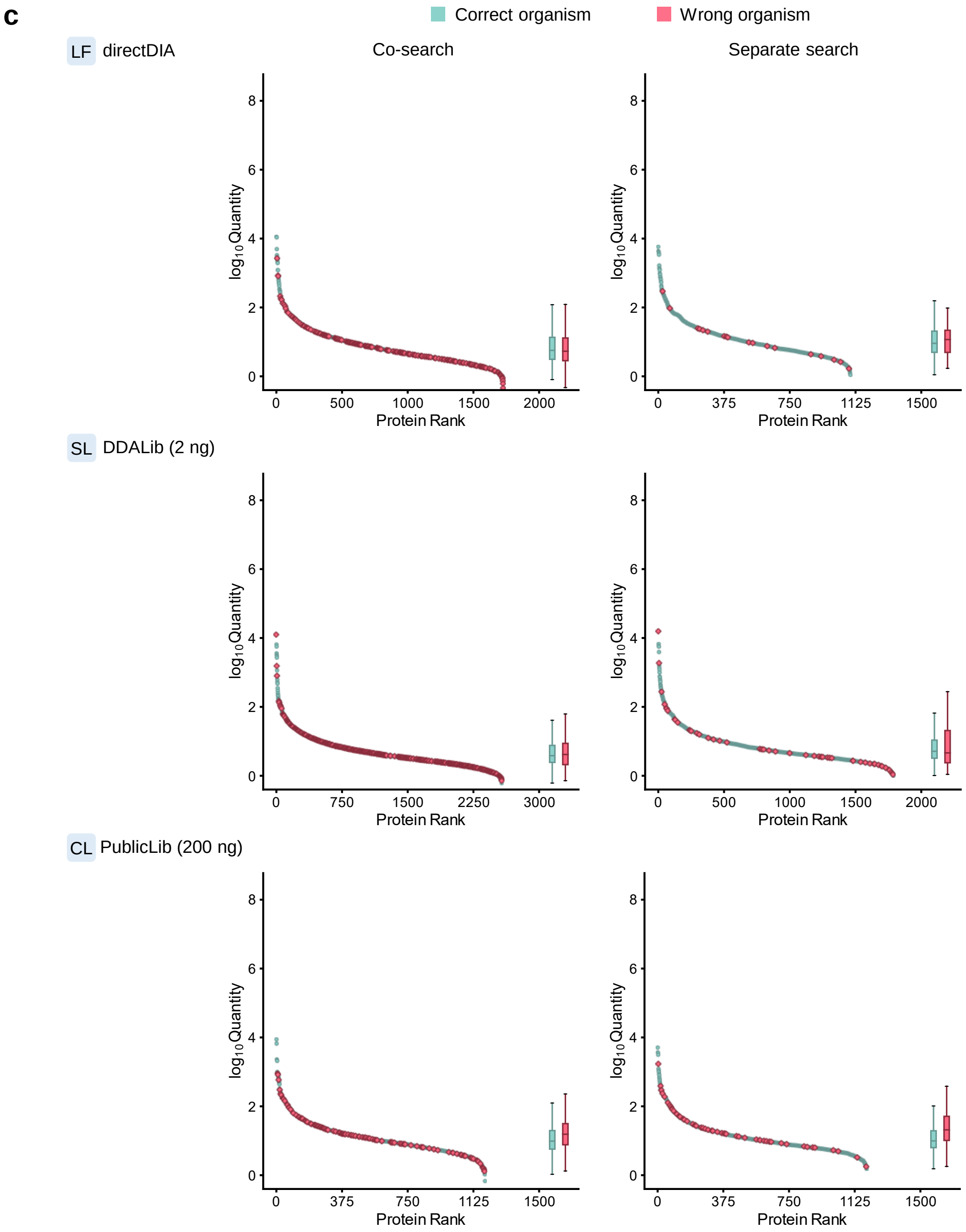

Figure SD2-29. (Continued from previous page)
c Organism-matched (in green) and potential false positive (in red) proteins ranked by their quantities (mean value across the runs for each sample). The boxes mark the first and third quantile and the lines inside the boxes mark the median; the whiskers extend from the box to the farthest point lying within 1.5 times the inter-quartile range.

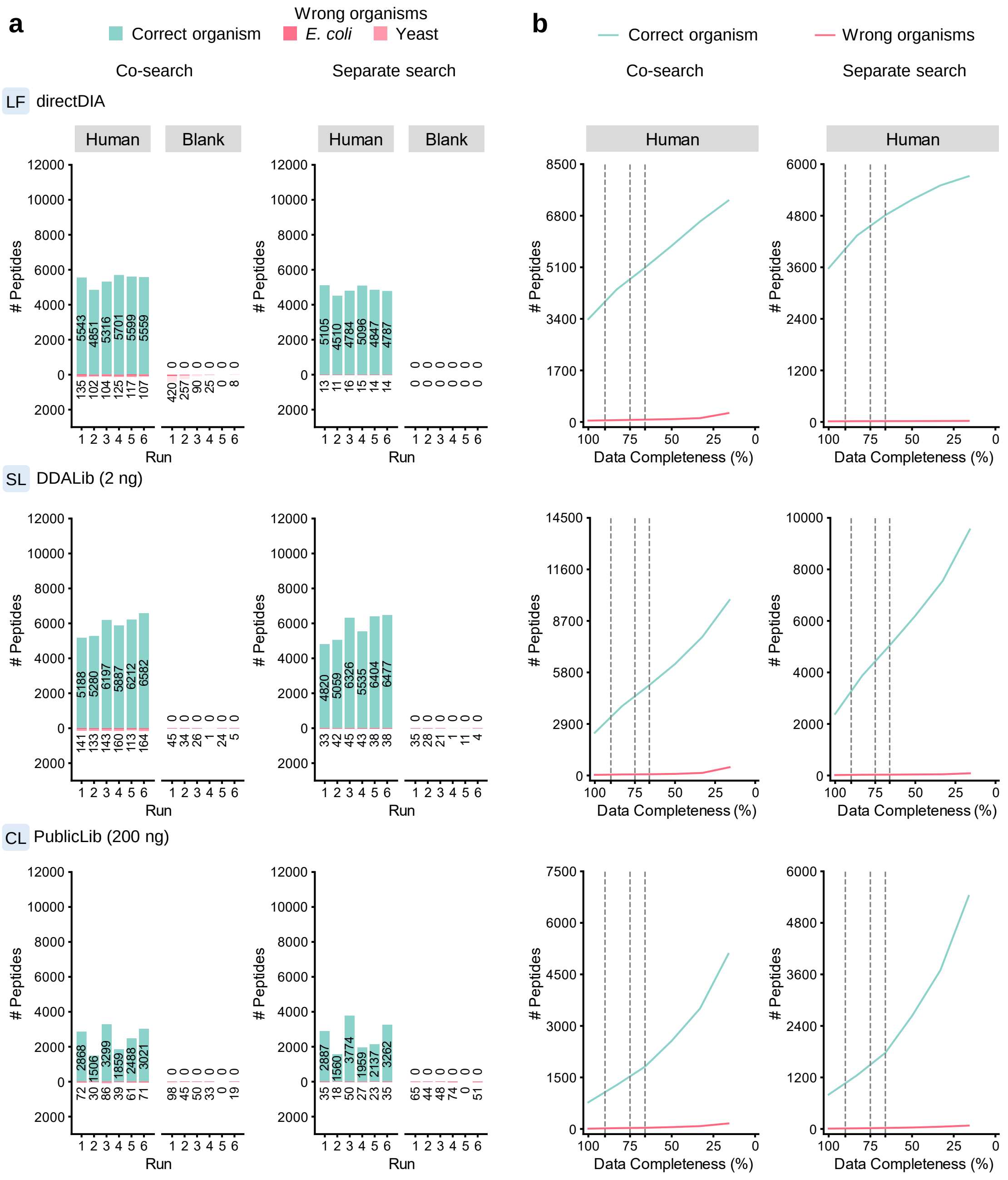

Figure SD2-30. Comparison of false positive detection by different searching strategies using Spectronaut at the peptide level.

a Numbers of quantified peptides per run. For each sample, correctly detected peptides should be from the organism specific to the sample (in green), while those from other organisms (in red) are potential false positives. Results of blank injections are shown to assess potential contaminants. **b** Numbers of organism-matched and potential false positive peptides quantified in at least specified percentages (data completeness) of runs. (Continued on next page)

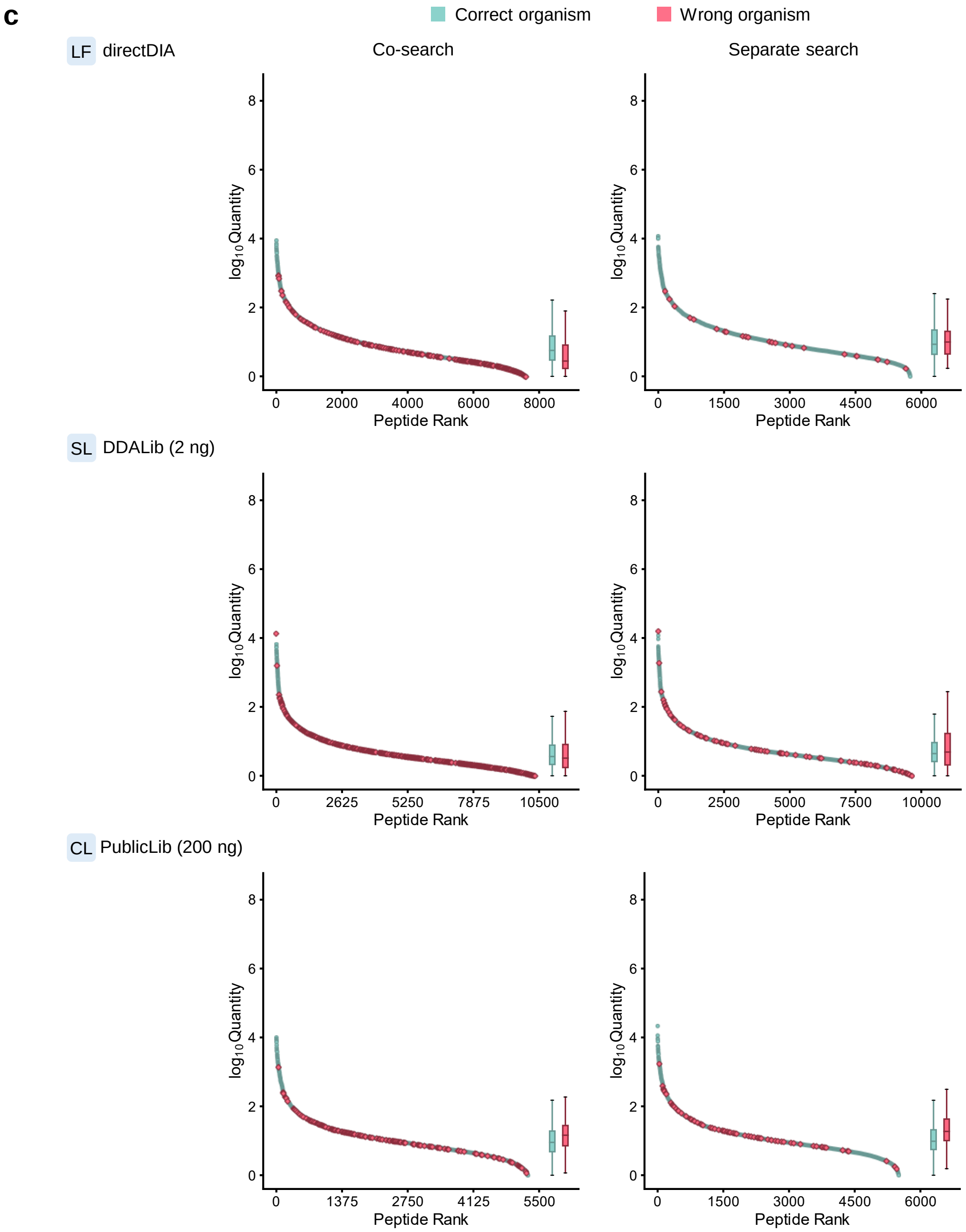

Figure SD2-30. (Continued from previous page)
c Organism-matched (in green) and potential false positive (in red) peptides ranked by their quantities (mean value across the runs for each sample). The boxes mark the first and third quantile and the lines inside the boxes mark the median; the whiskers extend from the box to the farthest point lying within 1.5 times the inter-quartile range.

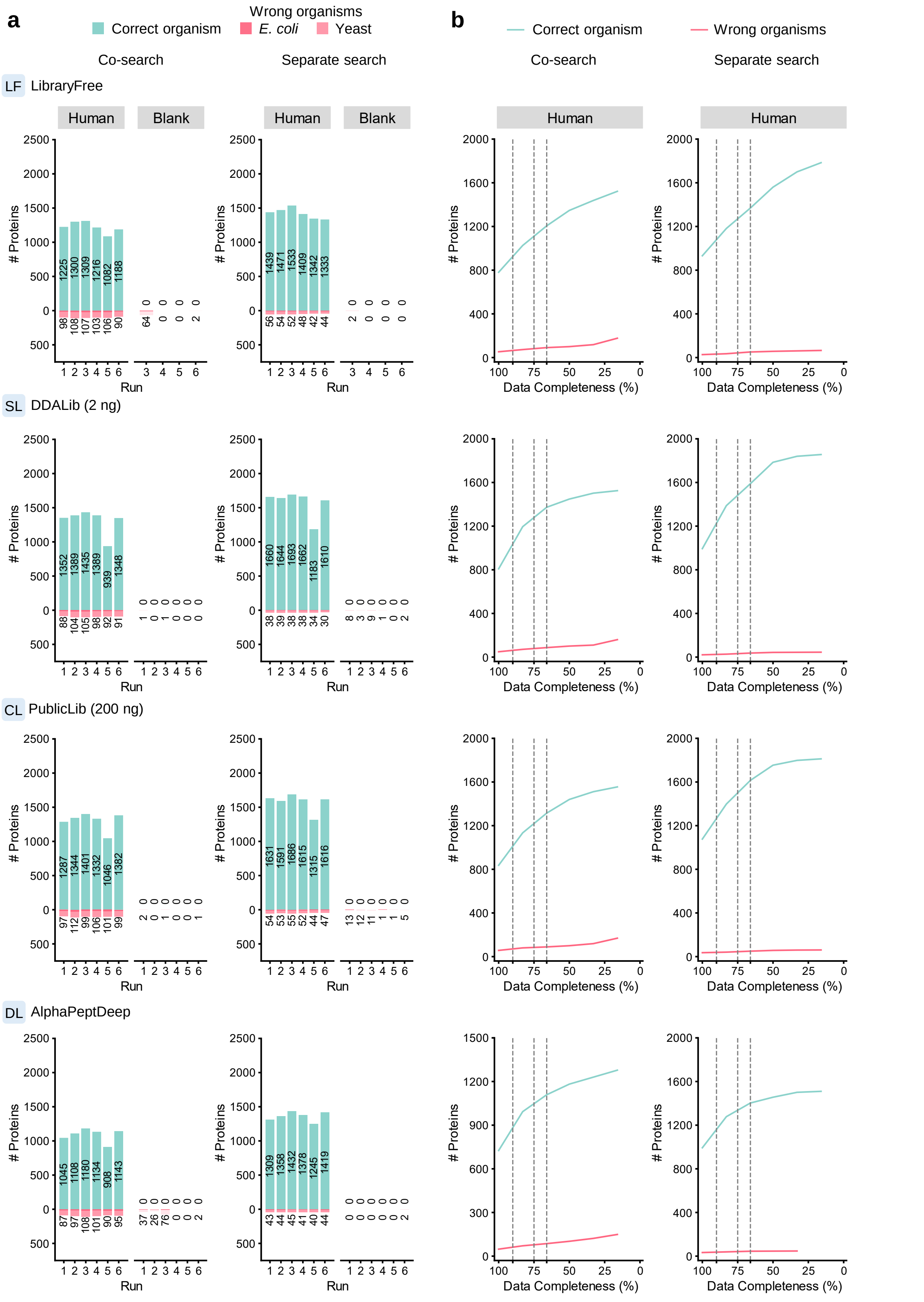

Figure SD2-31. Comparison of false positive detection by different searching strategies using PEAKS at the protein level.

a Numbers of quantified proteins per run. For each sample, correctly detected proteins should be from the organism specific to the sample (in green), while those from other organisms (in red) are potential false positives. Results of blank injections are shown to assess potential contaminants. **b** Numbers of organism-matched and potential false positive proteins quantified in at least specified percentages (data completeness) of runs. (Continued on next page)

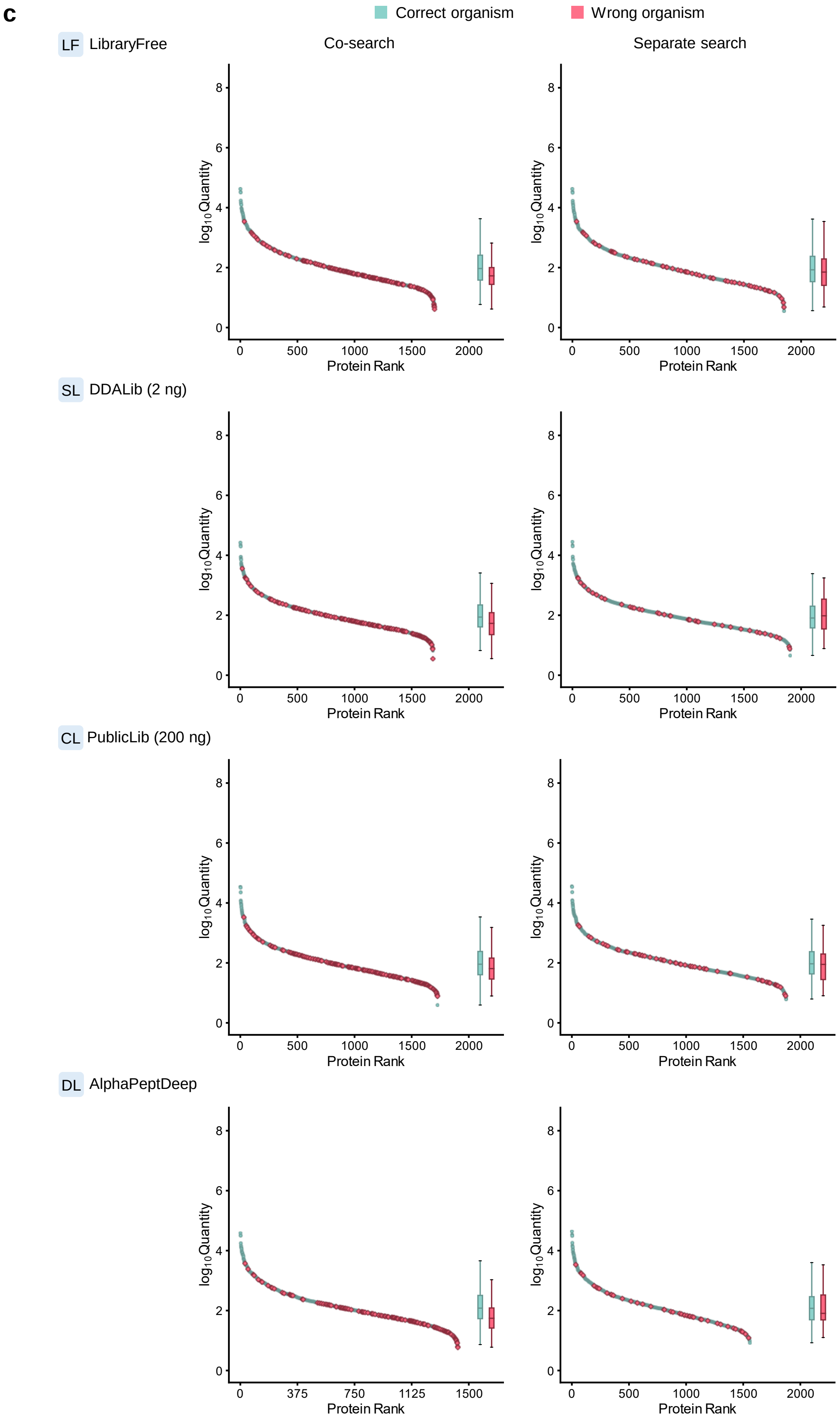

Figure SD2-31. (Continued from previous page)
c Organism-matched (in green) and potential false positive (in red) proteins ranked by their quantities (mean value across the runs for each sample). The boxes mark the first and third quantile and the lines inside the boxes mark the median; the whiskers extend from the box to the farthest point lying within 1.5 times the inter-quartile range.

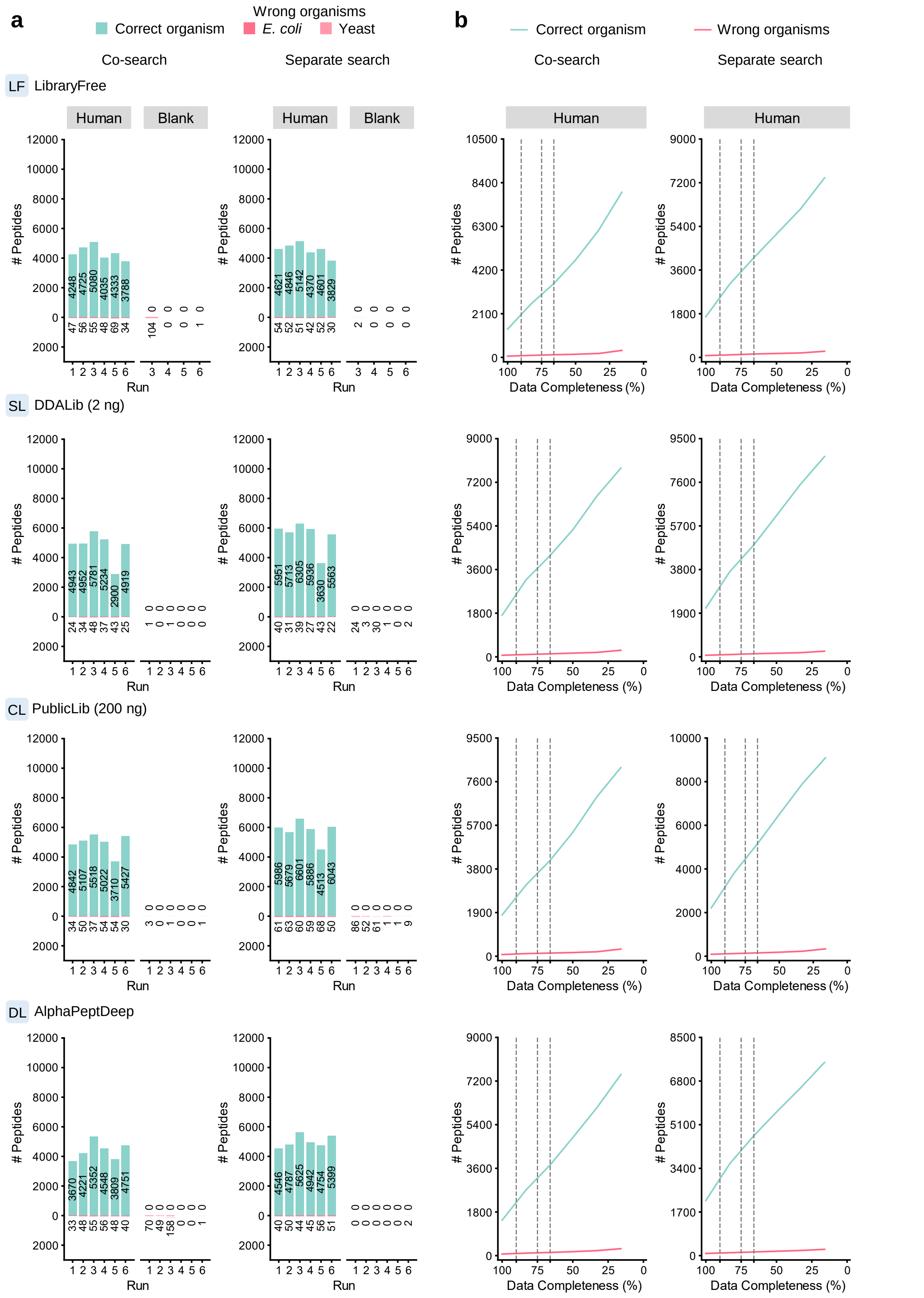

Figure SD2-32. Comparison of false positive detection by different searching strategies using PEAKS at the peptide level.

a Numbers of quantified peptides per run. For each sample, correctly detected peptides should be from the organism specific to the sample (in green), while those from other organisms (in red) are potential false positives. Results of blank injections are shown to assess potential contaminants. **b** Numbers of organism-matched and potential false positive peptides quantified in at least specified percentages (data completeness) of runs. (Continued on next page)

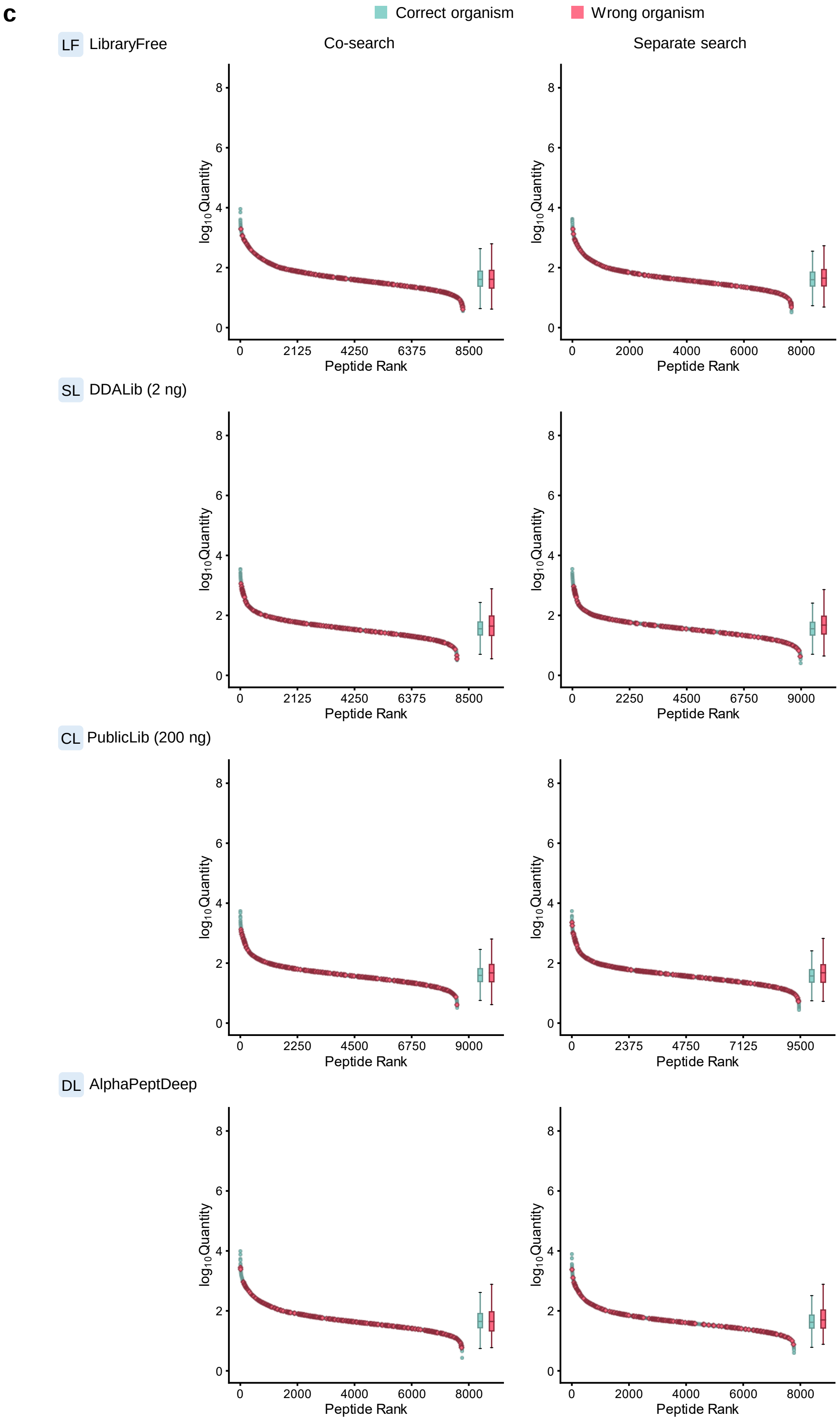

Figure SD2-32. (Continued from previous page)

c Organism-matched (in green) and potential false positive (in red) peptides ranked by their quantities (mean value across the runs for each sample). The boxes mark the first and third quantile and the lines inside the boxes mark the median; the whiskers extend from the box to the farthest point lying within 1.5 times the inter-quartile range.
