# Supplementary material for: Benchmarking informatics workflows for data-independent acquisition single-cell proteomics
Source: Nat Commun. 2025 Nov 21;16:10276. doi: 10.1038/s41467-025-65174-4 (PMC12639053; doi:10.1038/s41467-025-65174-4)
Supplement: Supplementary file 5 — Supplementary Data 3 [file 41467_2025_65174_MOESM5_ESM.zip › FigSD3-[1-7] DIA-NN Varying SR.pdf]

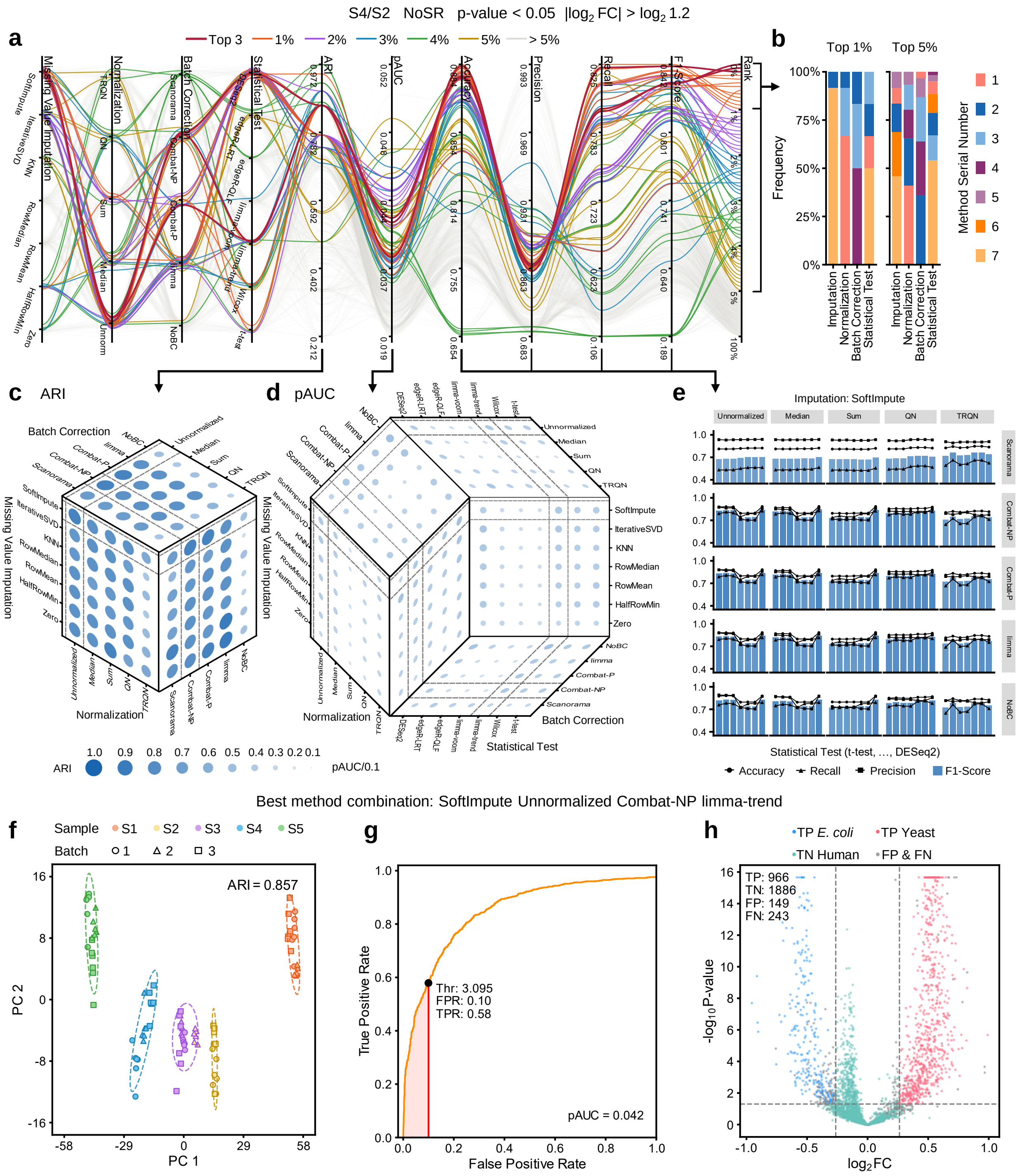

(Legend on next page)

Figure SD3-1. Performance comparison of method combinations for differential analysis (DIA-NN S4/S2 NoSR) **a** Parallel coordinate representation showing metrics using different method combinations. Line colors indicate the percentile rank of the method combinations. **b** Compositions of the top 1% and 5% method combinations in **a**. Mappings of the serial numbers to detailed methods for each step are present in Fig. 2a. **c** Adjusted Rand index (ARI) metrics. **d** Partial area under receiver operator characteristic curve (pAUC) metrics. In **c** and **d**, the metrics are visualized in a hyperbox, where each face displays the metrics with two steps variable and the other steps fixed to those of the best method combination. For the best method combination, the method choice in each step is marked with dashed lines. Dot sizes and colors indicate the metric values. **e** Accuracy (dots), recall (triangles), precision (squares), and F1-score (bars) metrics. Rows represent batch effect correction methods and columns represent normalization methods. The other steps are those of the best method combination. **f** Clustering result of the 5 groups of samples visualized using principal component analysis for dimension reduction. The fill colors indicate the sample groups and the shape indicate the batches. The border colors indicate the clusters. **g** Receiver operator characteristic (ROC) curves using −log10 p-value as scores. The optimal cut-offs with false positive rate (FPR) ≤ 0.1 are marked using black dots with score threshold (Thr), FPR, and true positive rate (TPR) values indicated. **h** Volcano plots. Blue dots represent TP *E. coli* proteins, red dots represent TP yeast proteins, green dots represent TN human proteins, and gray dots represent FP or FN proteins. For **f**–**h**, the data were processed through the best method combinations. Benchmarks are performed on protein quantification results by DIA-NN. The data are processed starting with NoSR. Differential analysis was performed between the S4 and S2 sample groups. Differential proteins are determined with p-value < 0.05 and $|\log_2 \mathrm{FC}| > \log_2 1.2$.

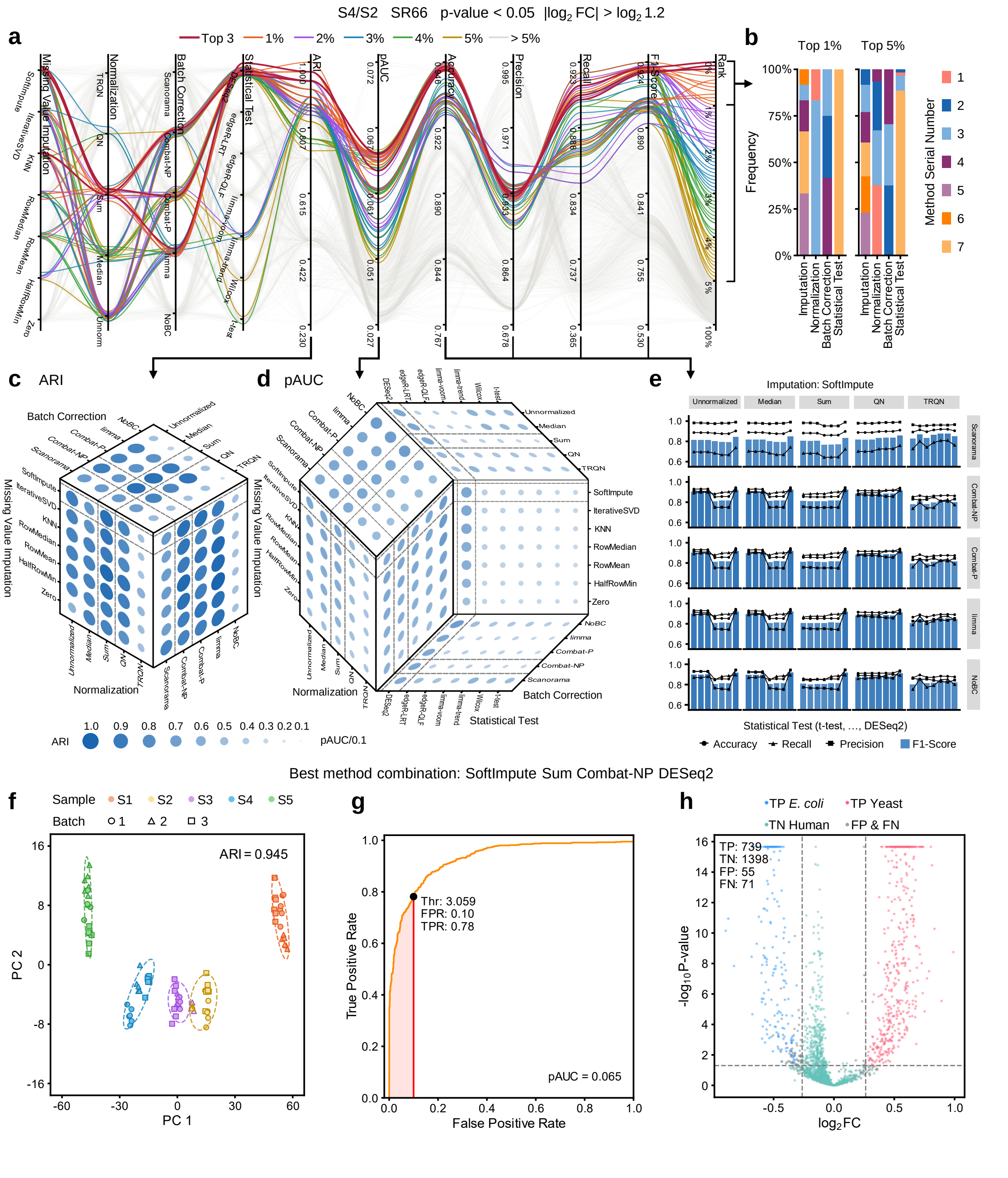

(Legend on next page)

Figure SD3-2. Performance comparison of method combinations for differential analysis (DIA-NN S4/S2 SR66) **a** Parallel coordinate representation showing metrics using different method combinations. Line colors indicate the percentile rank of the method combinations. **b** Compositions of the top 1% and 5% method combinations in **a**. Mappings of the serial numbers to detailed methods for each step are present in Fig. 2a. **c** Adjusted Rand index (ARI) metrics. **d** Partial area under receiver operator characteristic curve (pAUC) metrics. In **c** and **d**, the metrics are visualized in a hyperbox, where each face displays the metrics with two steps variable and the other steps fixed to those of the best method combination. For the best method combination, the method choice in each step is marked with dashed lines. Dot sizes and colors indicate the metric values. **e** Accuracy (dots), recall (triangles), precision (squares), and F1-score (bars) metrics. Rows represent batch effect correction methods and columns represent normalization methods. The other steps are those of the best method combination. **f** Clustering result of the 5 groups of samples visualized using principal component analysis for dimension reduction. The fill colors indicate the sample groups and the shape indicate the batches. The border colors indicate the clusters. **g** Receiver operator characteristic (ROC) curves using −log10 p-value as scores. The optimal cut-offs with false positive rate (FPR) ≤ 0.1 are marked using black dots with score threshold (Thr), FPR, and true positive rate (TPR) values indicated. **h** Volcano plots. Blue dots represent TP *E. coli* proteins, red dots represent TP yeast proteins, green dots represent TN human proteins, and gray dots represent FP or FN proteins. For **f**–**h**, the data were processed through the best method combinations. Benchmarks are performed on protein quantification results by DIA-NN. The data are processed starting with SR66. Differential analysis was performed between the S4 and S2 sample groups. Differential proteins are determined with p-value < 0.05 and $|\log_2 FC| > \log_2 1.2$.

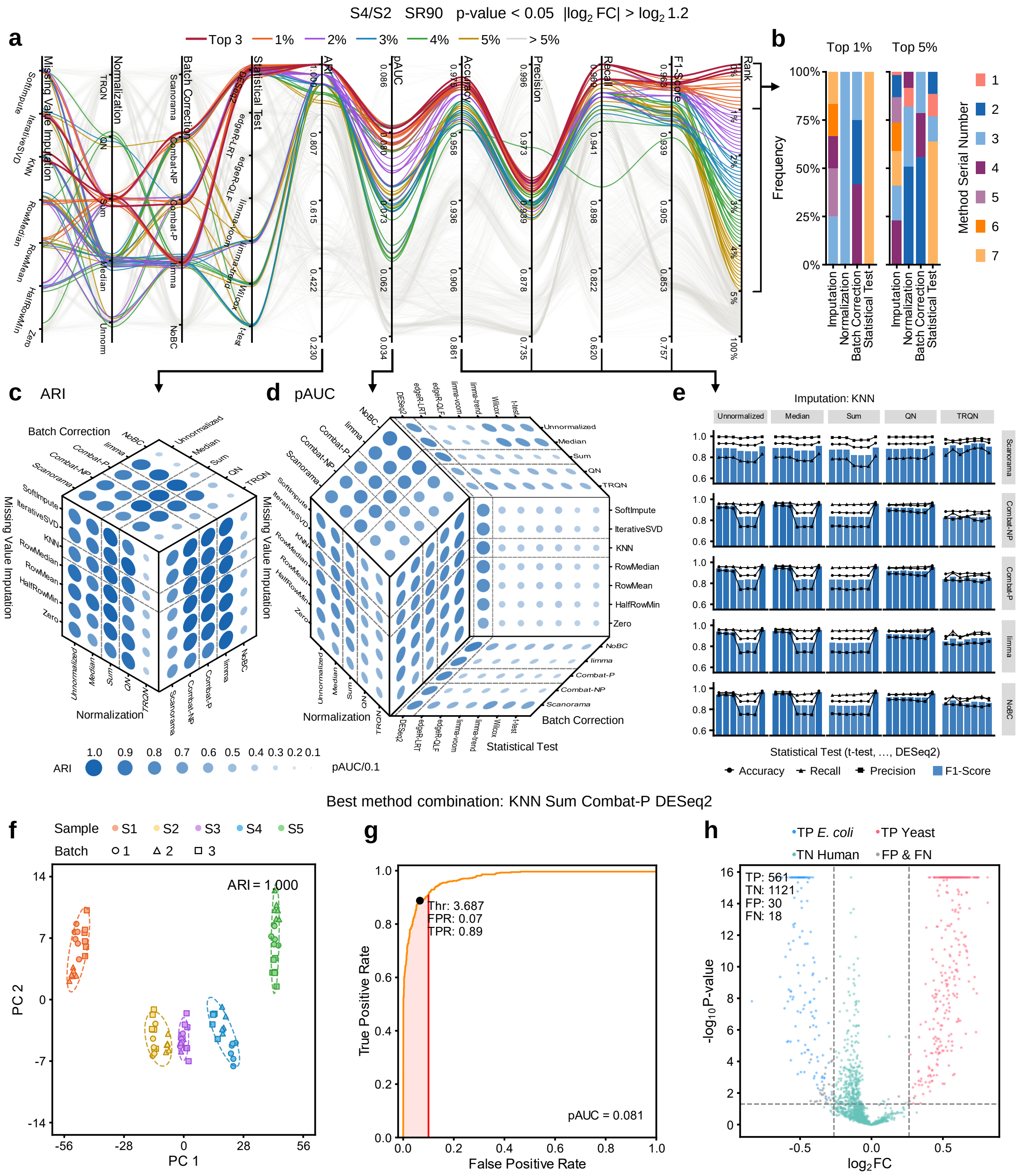

(Legend on next page)

Figure SD3-3. Performance comparison of method combinations for differential analysis (DIA-NN S4/S2 SR90) **a** Parallel coordinate representation showing metrics using different method combinations. Line colors indicate the percentile rank of the method combinations. **b** Compositions of the top 1% and 5% method combinations in **a**. Mappings of the serial numbers to detailed methods for each step are present in Fig. 2a. **c** Adjusted Rand index (ARI) metrics. **d** Partial area under receiver operator characteristic curve (pAUC) metrics. In **c** and **d**, the metrics are visualized in a hyperbox, where each face displays the metrics with two steps variable and the other steps fixed to those of the best method combination. For the best method combination, the method choice in each step is marked with dashed lines. Dot sizes and colors indicate the metric values. **e** Accuracy (dots), recall (triangles), precision (squares), and F1-score (bars) metrics. Rows represent batch effect correction methods and columns represent normalization methods. The other steps are those of the best method combination. **f** Clustering result of the 5 groups of samples visualized using principal component analysis for dimension reduction. The fill colors indicate the sample groups and the shape indicate the batches. The border colors indicate the clusters. **g** Receiver operator characteristic (ROC) curves using −log10 p-value as scores. The optimal cut-offs with false positive rate (FPR) ≤ 0.1 are marked using black dots with score threshold (Thr), FPR, and true positive rate (TPR) values indicated. **h** Volcano plots. Blue dots represent TP *E. coli* proteins, red dots represent TP yeast proteins, green dots represent TN human proteins, and gray dots represent FP or FN proteins. For **f**–**h**, the data were processed through the best method combinations. Benchmarks are performed on protein quantification results by DIA-NN. The data are processed starting with SR90. Differential analysis was performed between the S4 and S2 sample groups. Differential proteins are determined with p-value < 0.05 and $|\log_2 \mathrm{FC}| > \log_2 1.2$.

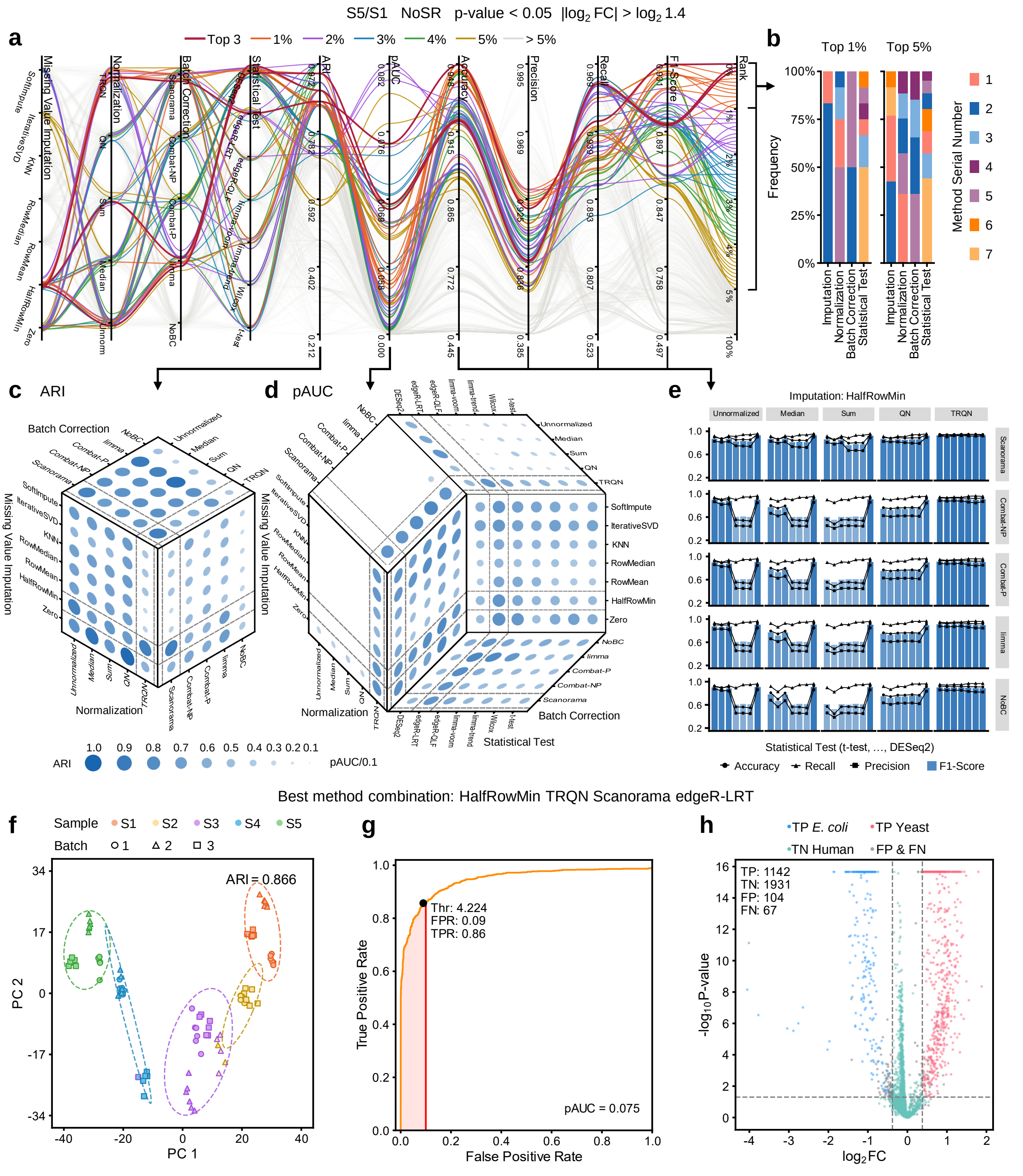

(Legend on next page)

Figure SD3-4. Performance comparison of method combinations for differential analysis (DIA-NN S5/S1 NoSR) **a** Parallel coordinate representation showing metrics using different method combinations. Line colors indicate the percentile rank of the method combinations. **b** Compositions of the top 1% and 5% method combinations in **a**. Mappings of the serial numbers to detailed methods for each step are present in Fig. 2a. **c** Adjusted Rand index (ARI) metrics. **d** Partial area under receiver operator characteristic curve (pAUC) metrics. In **c** and **d**, the metrics are visualized in a hyperbox, where each face displays the metrics with two steps variable and the other steps fixed to those of the best method combination. For the best method combination, the method choice in each step is marked with dashed lines. Dot sizes and colors indicate the metric values. **e** Accuracy (dots), recall (triangles), precision (squares), and F1-score (bars) metrics. Rows represent batch effect correction methods and columns represent normalization methods. The other steps are those of the best method combination. **f** Clustering result of the 5 groups of samples visualized using principal component analysis for dimension reduction. The fill colors indicate the sample groups and the shape indicate the batches. The border colors indicate the clusters. **g** Receiver operator characteristic (ROC) curves using −log10 p-value as scores. The optimal cut-offs with false positive rate (FPR) ≤ 0.1 are marked using black dots with score threshold (Thr), FPR, and true positive rate (TPR) values indicated. **h** Volcano plots. Blue dots represent TP *E. coli* proteins, red dots represent TP yeast proteins, green dots represent TN human proteins, and gray dots represent FP or FN proteins. For **f**–**h**, the data were processed through the best method combinations. Benchmarks are performed on protein quantification results by DIA-NN. The data are processed starting with NoSR. Differential analysis was performed between the S5 and S1 sample groups. Differential proteins are determined with p-value < 0.05 and $|\log_2 FC| > \log_2 1.4$.

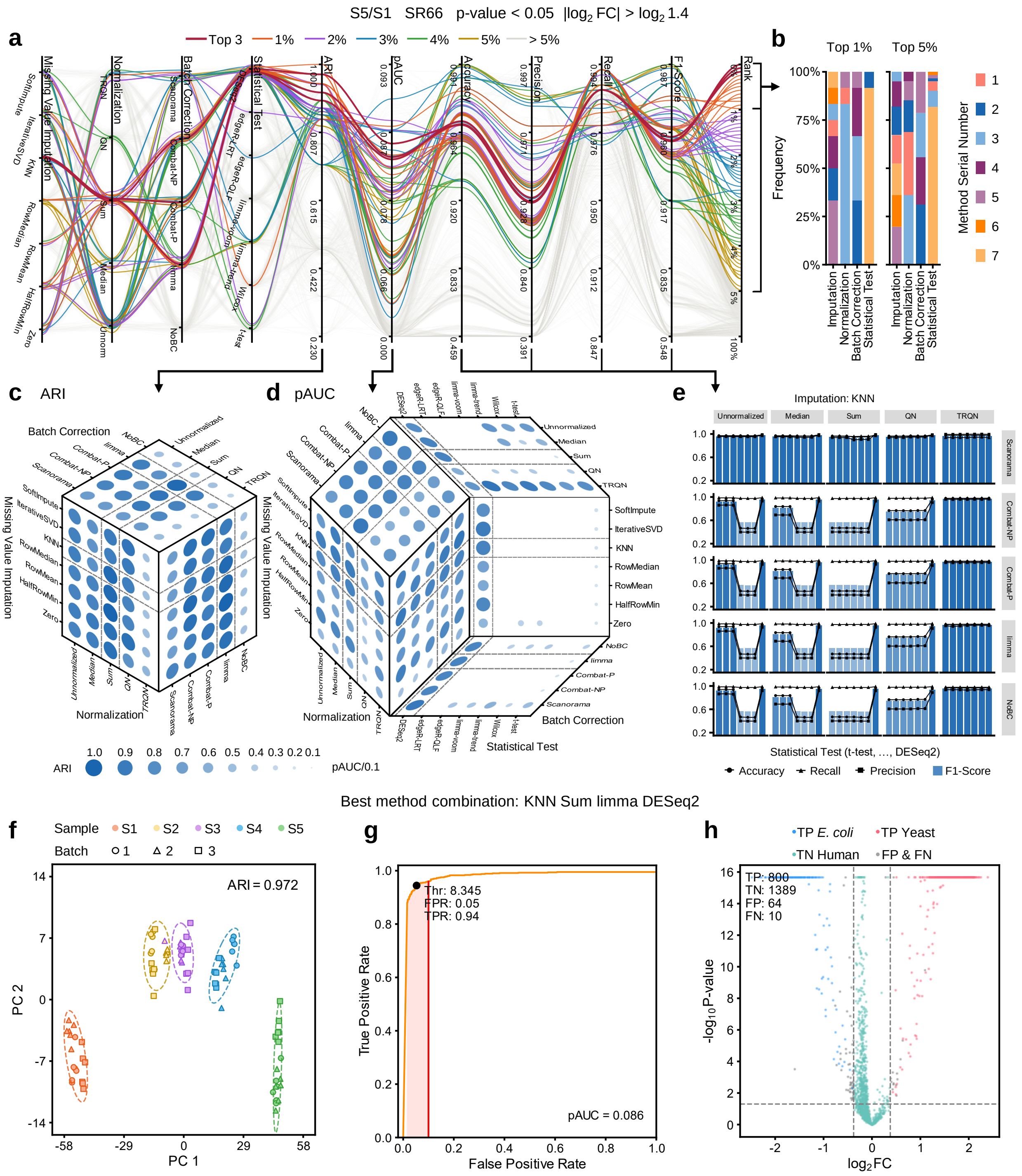

(Legend on next page)

Figure SD3-5. Performance comparison of method combinations for differential analysis (DIA-NN S5/S1 SR66) **a** Parallel coordinate representation showing metrics using different method combinations. Line colors indicate the percentile rank of the method combinations. **b** Compositions of the top 1% and 5% method combinations in **a**. Mappings of the serial numbers to detailed methods for each step are present in Fig. 2a. **c** Adjusted Rand index (ARI) metrics. **d** Partial area under receiver operator characteristic curve (pAUC) metrics. In **c** and **d**, the metrics are visualized in a hyperbox, where each face displays the metrics with two steps variable and the other steps fixed to those of the best method combination. For the best method combination, the method choice in each step is marked with dashed lines. Dot sizes and colors indicate the metric values. **e** Accuracy (dots), recall (triangles), precision (squares), and F1-score (bars) metrics. Rows represent batch effect correction methods and columns represent normalization methods. The other steps are those of the best method combination. **f** Clustering result of the 5 groups of samples visualized using principal component analysis for dimension reduction. The fill colors indicate the sample groups and the shape indicate the batches. The border colors indicate the clusters. **g** Receiver operator characteristic (ROC) curves using −log10 p-value as scores. The optimal cut-offs with false positive rate (FPR) ≤ 0.1 are marked using black dots with score threshold (Thr), FPR, and true positive rate (TPR) values indicated. **h** Volcano plots. Blue dots represent TP *E. coli* proteins, red dots represent TP yeast proteins, green dots represent TN human proteins, and gray dots represent FP or FN proteins. For **f**–**h**, the data were processed through the best method combinations. Benchmarks are performed on protein quantification results by DIA-NN. The data are processed starting with SR66. Differential analysis was performed between the S5 and S1 sample groups. Differential proteins are determined with p-value < 0.05 and $|\log_2 FC| > \log_2 1.4$.

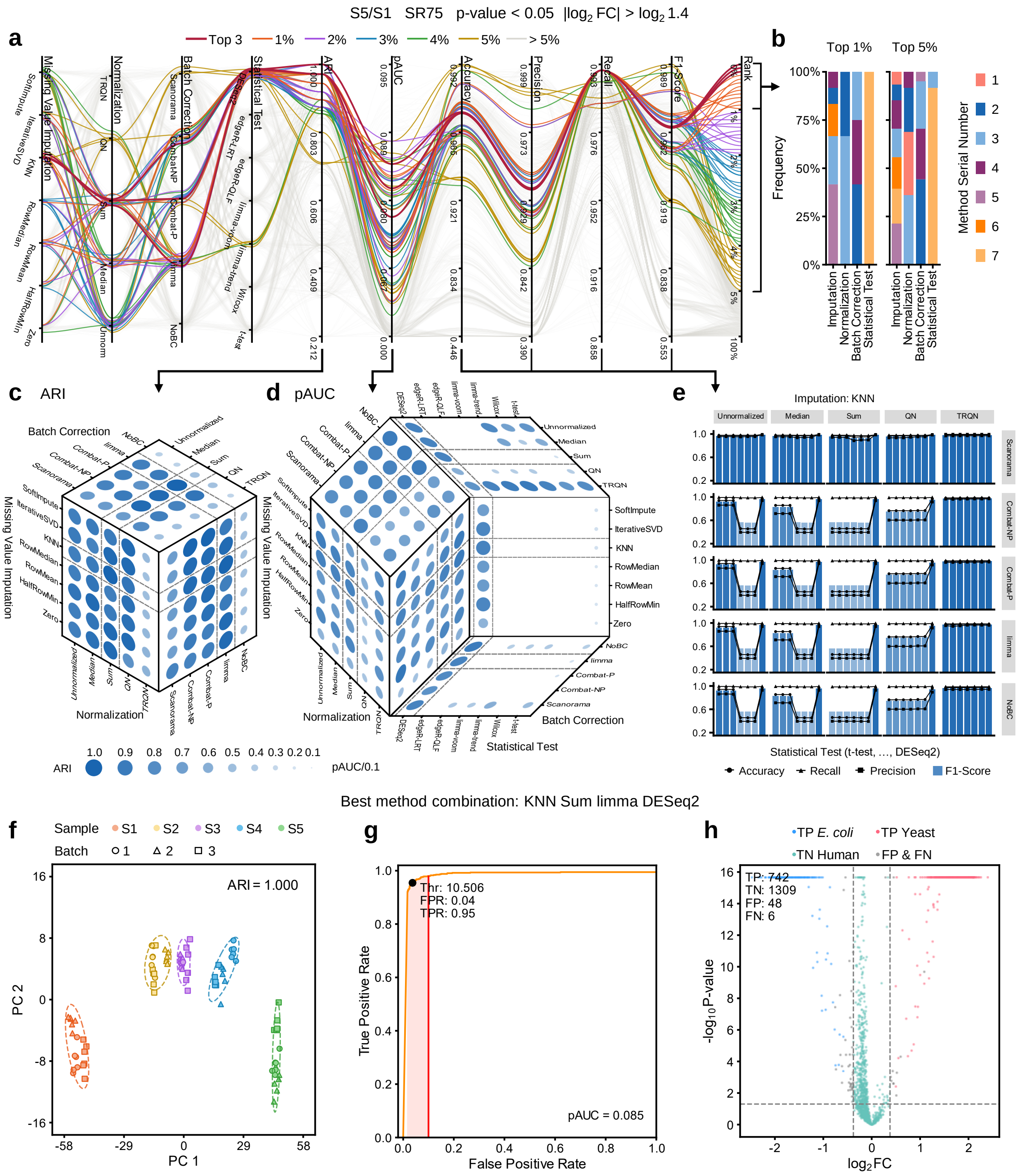

(Legend on next page)

Figure SD3-6. Performance comparison of method combinations for differential analysis (DIA-NN S5/S1 SR75) **a** Parallel coordinate representation showing metrics using different method combinations. Line colors indicate the percentile rank of the method combinations. **b** Compositions of the top 1% and 5% method combinations in **a**. Mappings of the serial numbers to detailed methods for each step are present in Fig. 2a. **c** Adjusted Rand index (ARI) metrics. **d** Partial area under receiver operator characteristic curve (pAUC) metrics. In **c** and **d**, the metrics are visualized in a hyperbox, where each face displays the metrics with two steps variable and the other steps fixed to those of the best method combination. For the best method combination, the method choice in each step is marked with dashed lines. Dot sizes and colors indicate the metric values. **e** Accuracy (dots), recall (triangles), precision (squares), and F1-score (bars) metrics. Rows represent batch effect correction methods and columns represent normalization methods. The other steps are those of the best method combination. **f** Clustering result of the 5 groups of samples visualized using principal component analysis for dimension reduction. The fill colors indicate the sample groups and the shape indicate the batches. The border colors indicate the clusters. **g** Receiver operator characteristic (ROC) curves using −log10 p-value as scores. The optimal cut-offs with false positive rate (FPR) ≤ 0.1 are marked using black dots with score threshold (Thr), FPR, and true positive rate (TPR) values indicated. **h** Volcano plots. Blue dots represent TP *E. coli* proteins, red dots represent TP yeast proteins, green dots represent TN human proteins, and gray dots represent FP or FN proteins. For **f**–**h**, the data were processed through the best method combinations. Benchmarks are performed on protein quantification results by DIA-NN. The data are processed starting with SR75. Differential analysis was performed between the S5 and S1 sample groups. Differential proteins are determined with p-value < 0.05 and $|\log_2 FC| > \log_2 1.4$.

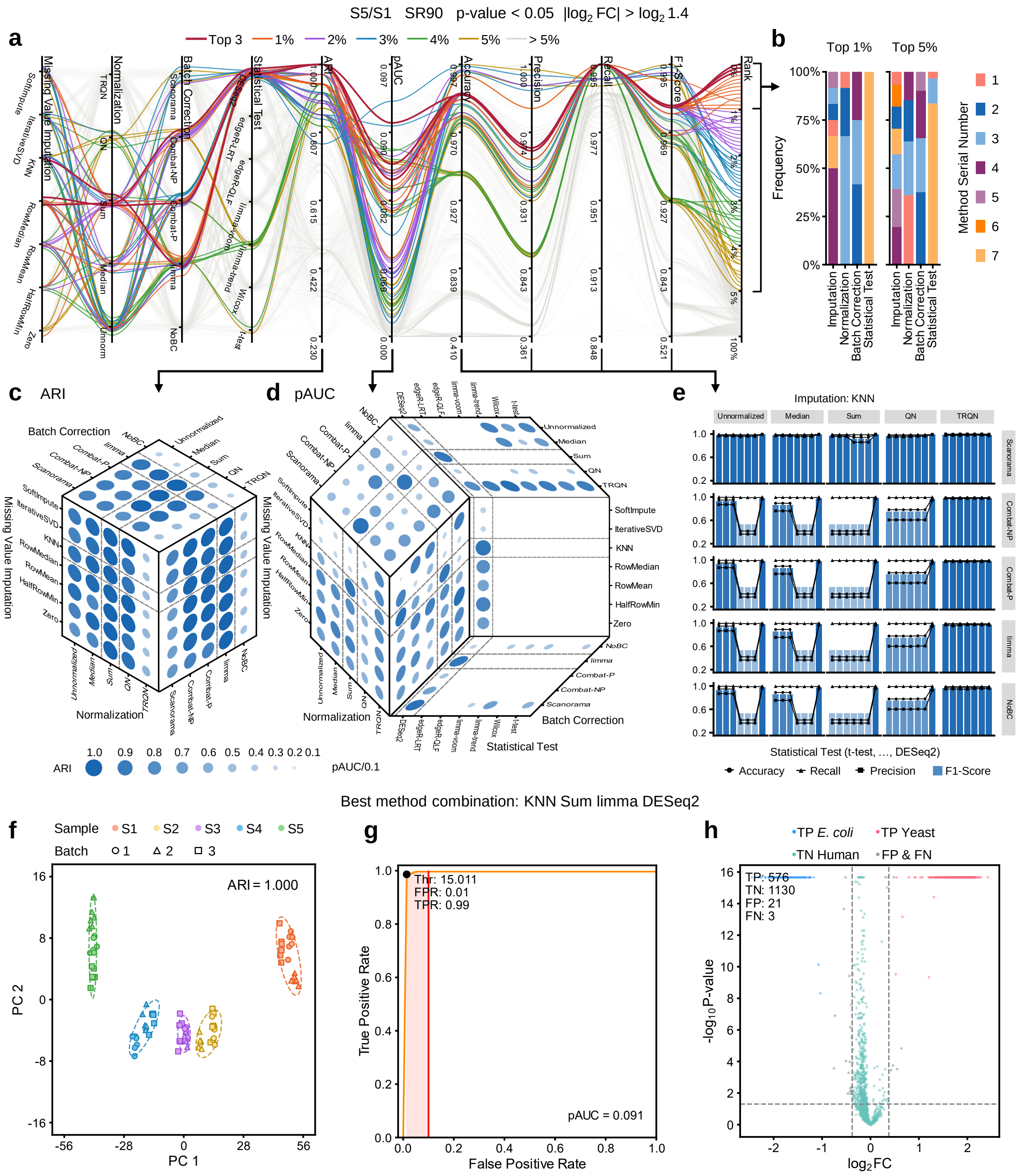

(Legend on next page)

Figure SD3-7. Performance comparison of method combinations for differential analysis (DIA-NN S5/S1 SR90) **a** Parallel coordinate representation showing metrics using different method combinations. Line colors indicate the percentile rank of the method combinations. **b** Compositions of the top 1% and 5% method combinations in **a**. Mappings of the serial numbers to detailed methods for each step are present in Fig. 2a. **c** Adjusted Rand index (ARI) metrics. **d** Partial area under receiver operator characteristic curve (pAUC) metrics. In **c** and **d**, the metrics are visualized in a hyperbox, where each face displays the metrics with two steps variable and the other steps fixed to those of the best method combination. For the best method combination, the method choice in each step is marked with dashed lines. Dot sizes and colors indicate the metric values. **e** Accuracy (dots), recall (triangles), precision (squares), and F1-score (bars) metrics. Rows represent batch effect correction methods and columns represent normalization methods. The other steps are those of the best method combination. **f** Clustering result of the 5 groups of samples visualized using principal component analysis for dimension reduction. The fill colors indicate the sample groups and the shape indicate the batches. The border colors indicate the clusters. **g** Receiver operator characteristic (ROC) curves using −log10 p-value as scores. The optimal cut-offs with false positive rate (FPR) ≤ 0.1 are marked using black dots with score threshold (Thr), FPR, and true positive rate (TPR) values indicated. **h** Volcano plots. Blue dots represent TP *E. coli* proteins, red dots represent TP yeast proteins, green dots represent TN human proteins, and gray dots represent FP or FN proteins. For **f**–**h**, the data were processed through the best method combinations. Benchmarks are performed on protein quantification results by DIA-NN. The data are processed starting with SR90. Differential analysis was performed between the S5 and S1 sample groups. Differential proteins are determined with p-value < 0.05 and $|\log_2 FC| > \log_2 1.4$.
