# Supplementary material for: Benchmarking informatics workflows for data-independent acquisition single-cell proteomics
Source: Nat Commun. 2025 Nov 21;16:10276. doi: 10.1038/s41467-025-65174-4 (PMC12639053; doi:10.1038/s41467-025-65174-4)
Supplement: Supplementary file 8 — Supplementary Data 6 [file 41467_2025_65174_MOESM8_ESM.zip › FigSD6-[1-3] Method Selection.pdf]

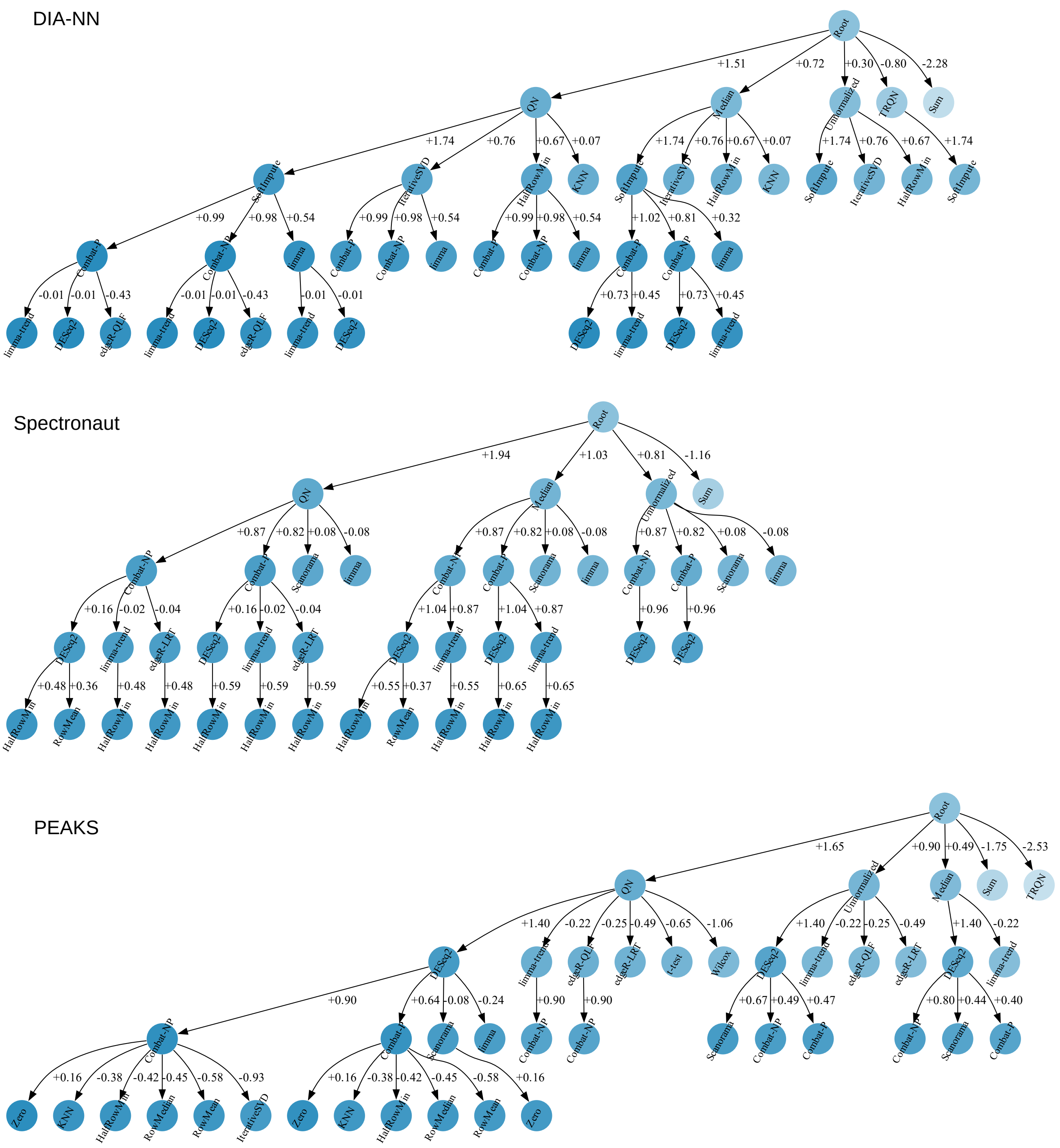

Figure SD6-1. Selection of the high-performing method combinations based on the benchmarking results (NoSR) From the benchmarking result (S4/S2, NoSR) of the simulated samples (batches of sample preparation with independent digestion) searched by each software, 12 method combinations (~1% of all the method combinations) were selected by beam search.

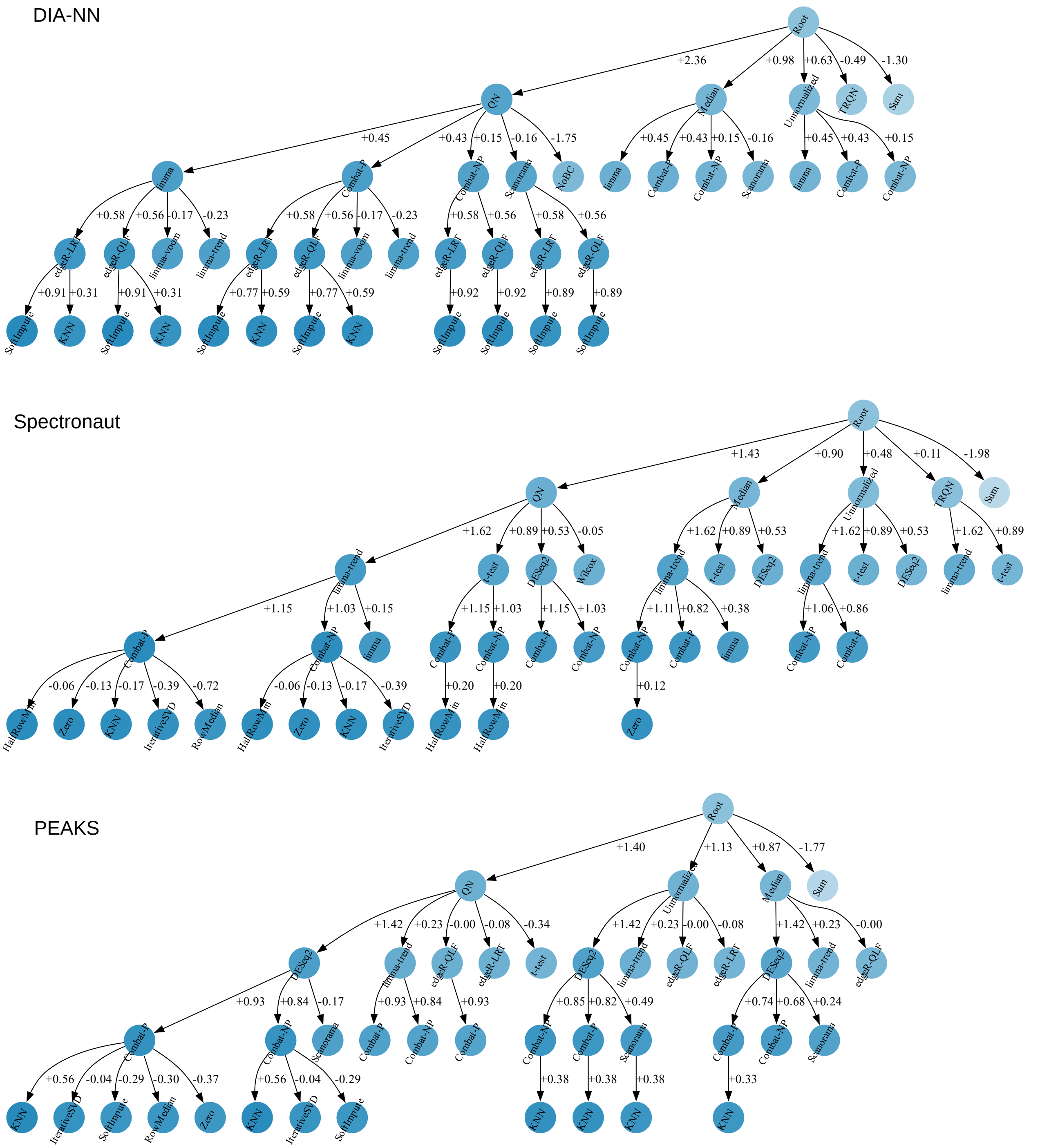

Figure SD6-2. Selection of the high-performing method combinations based on the benchmarking results (SR66) From the benchmarking result (S4/S2, SR66) of the simulated samples (batches of sample preparation with independent digestion) searched by each software, 12 method combinations (~1% of all the method combinations) were selected by beam search.

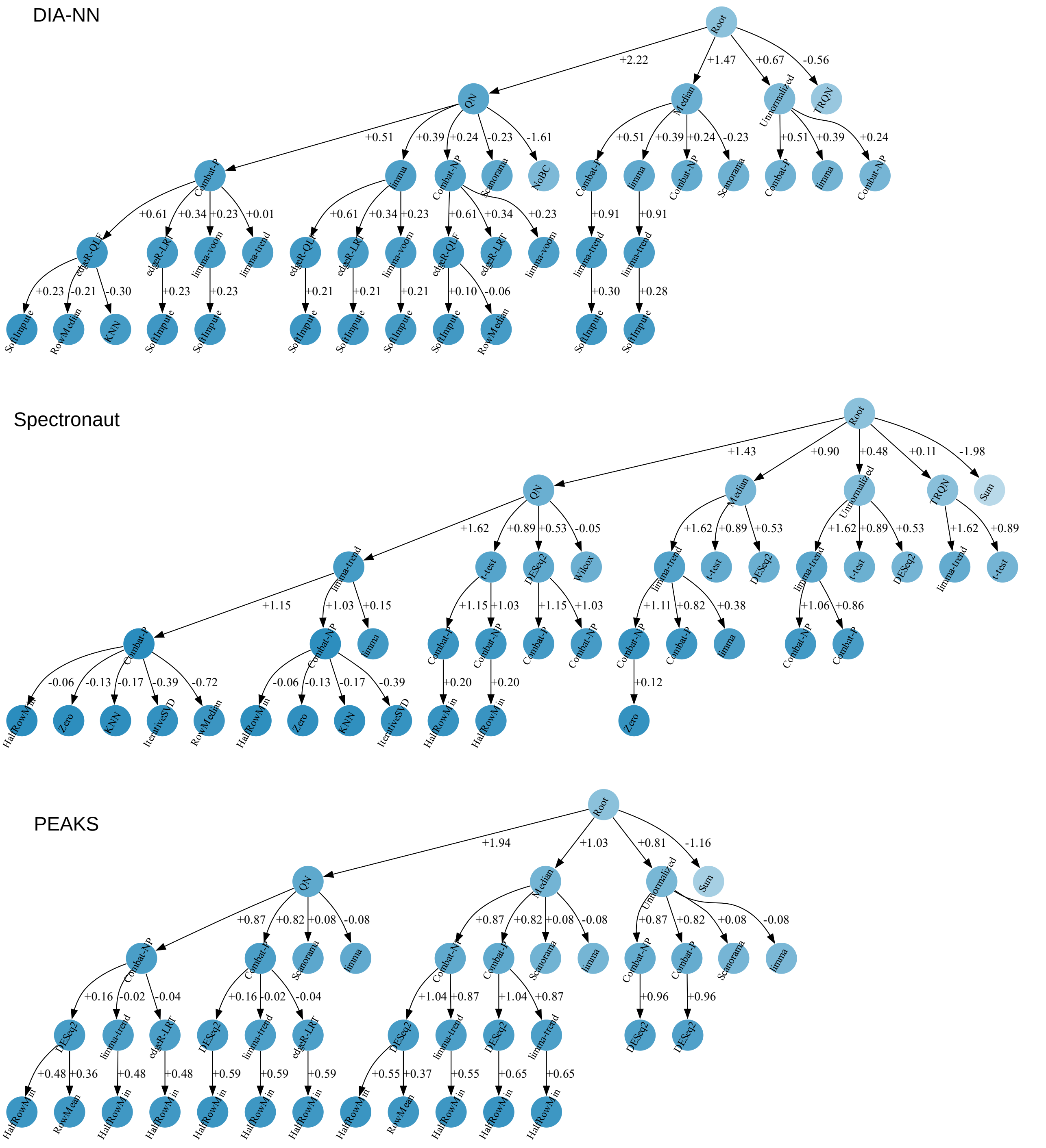

Figure SD6-3. Selection of the high-performing method combinations based on the benchmarking results (SR90) From the benchmarking result (S4/S2, SR90) of the simulated samples (batches of sample preparation with independent digestion) searched by each software, 12 method combinations (~1% of all the method combinations) were selected by beam search.
