# Supplementary material for: Benchmarking informatics workflows for data-independent acquisition single-cell proteomics
Source: Nat Commun. 2025 Nov 21;16:10276. doi: 10.1038/s41467-025-65174-4 (PMC12639053; doi:10.1038/s41467-025-65174-4)
Supplement: Supplementary file 8 — Supplementary Data 6 [file 41467_2025_65174_MOESM8_ESM.zip › FigSD6-[4-6] SpikeIn Metrics.pdf]

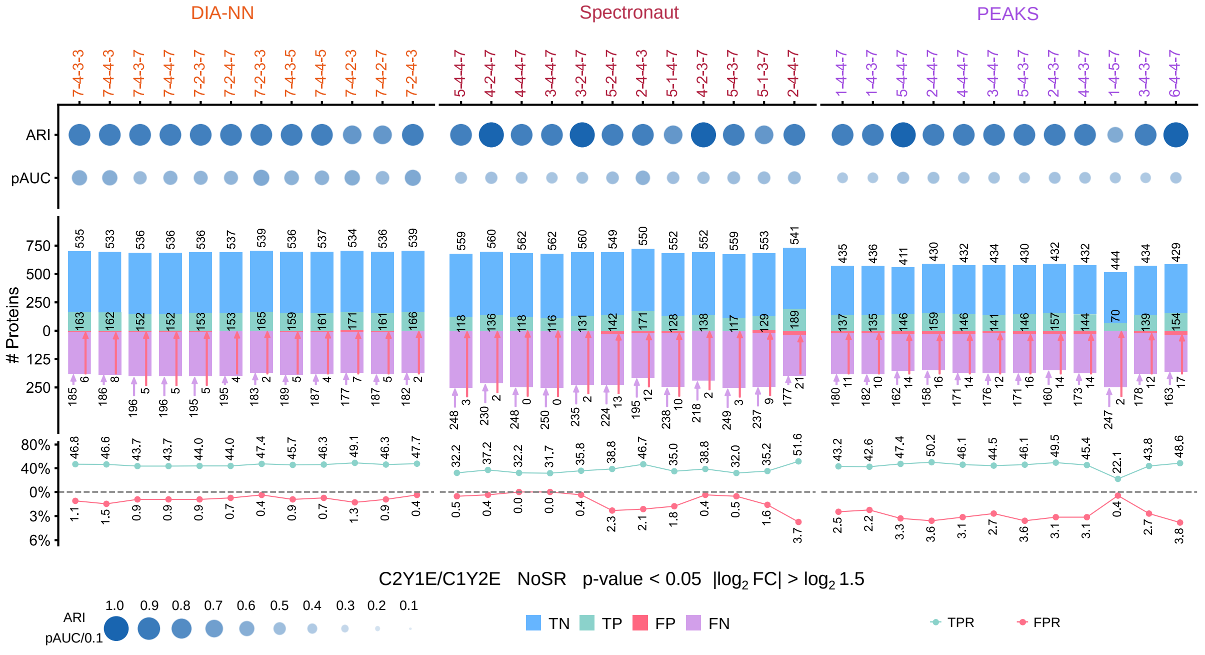

Figure SD6-4. Performance evaluation of high-performing method combinations on spike-in single-cell samples (NoSR)

Performance of the selected high-performing method combinations on the spike-in single-cell samples. Metrics include: the ARI and pAUC values (indicated by dot sizes and colors), numbers of detected TN (blue bars), TP (green bars), FP (red bars), and FN (purple bars) proteins, as well as TPR (green lines) and FPR (red lines) values. Mappings of the serial numbers to detailed methods for each step are present in Fig 2a. The data are processed starting with NoSR. Differential analysis was performed between the C2Y1E and C1Y2E sample groups. Differential proteins are determined with p-value < 0.05 and $|\log_2 FC| > \log_2 1.5$.

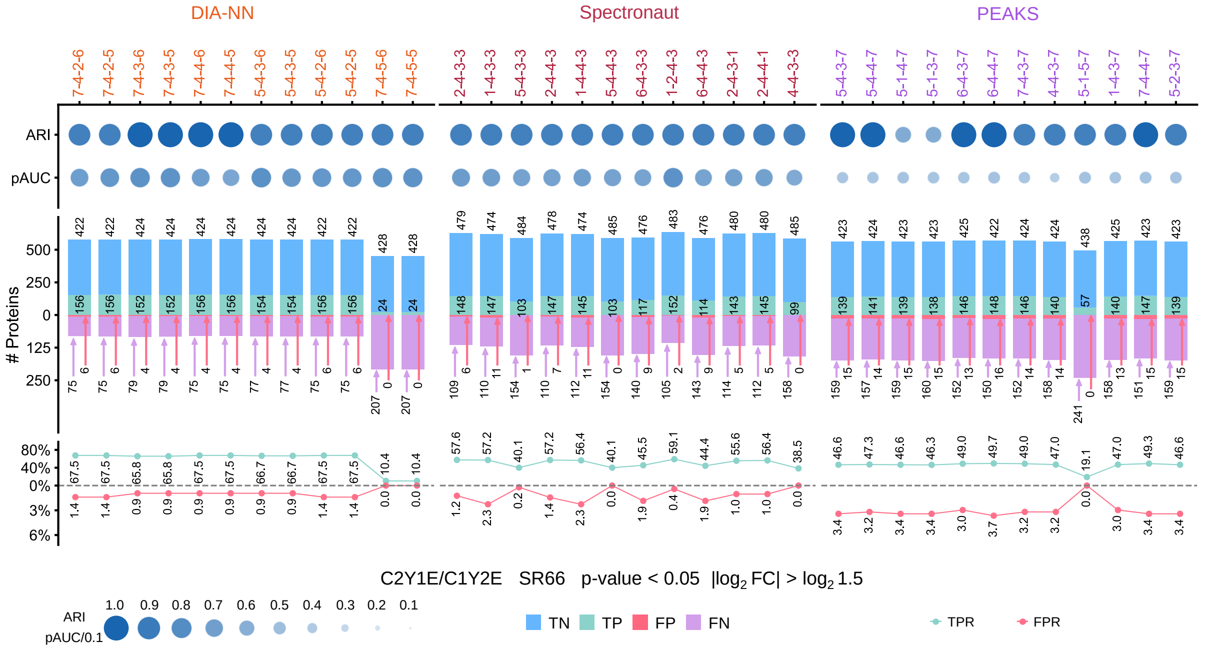

Figure SD6-5. Performance evaluation of high-performing method combinations on spike-in single-cell samples (SR66)

Performance of the selected high-performing method combinations on the spike-in single-cell samples. Metrics include: the ARI and pAUC values (indicated by dot sizes and colors), numbers of detected TN (blue bars), TP (green bars), FP (red bars), and FN (purple bars) proteins, as well as TPR (green lines) and FPR (red lines) values. Mappings of the serial numbers to detailed methods for each step are present in Fig 2a. The data are processed starting with SR66. Differential analysis was performed between the C2Y1E and C1Y2E sample groups. Differential proteins are determined with p-value < 0.05 and $|\log_2 FC| > \log_2 1.5$.

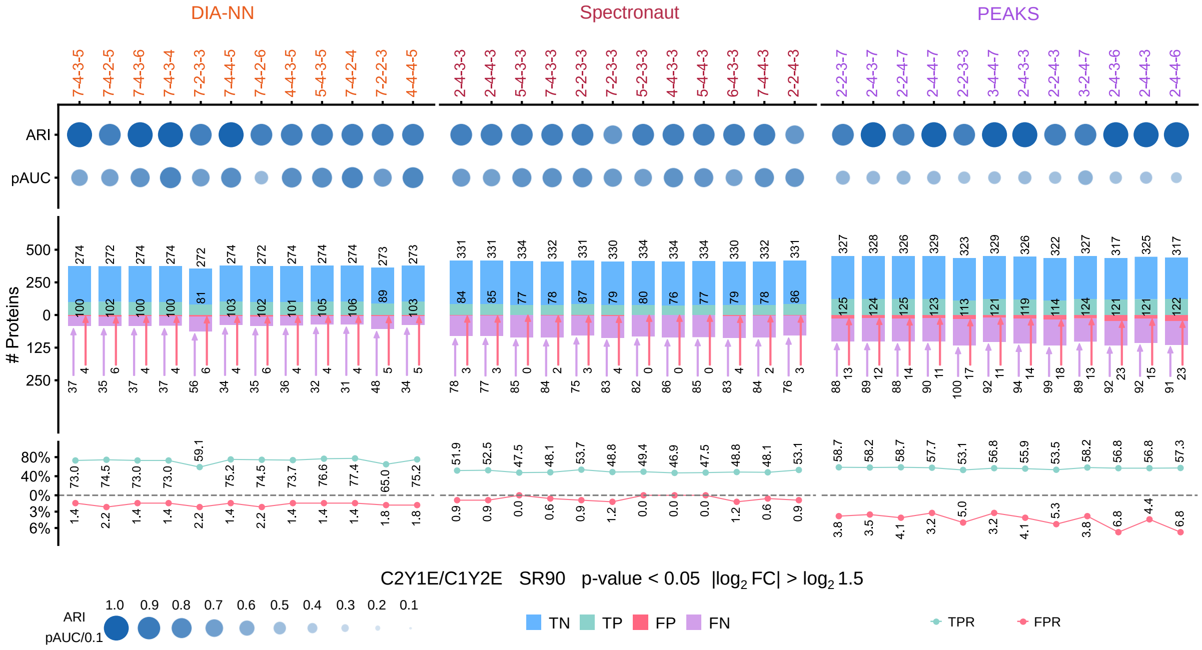

Figure SD6-6. Performance evaluation of high-performing method combinations on spike-in single-cell samples (SR90)

Performance of the selected high-performing method combinations on the spike-in single-cell samples. Metrics include: the ARI and pAUC values (indicated by dot sizes and colors), numbers of detected TN (blue bars), TP (green bars), FP (red bars), and FN (purple bars) proteins, as well as TPR (green lines) and FPR (red lines) values. Mappings of the serial numbers to detailed methods for each step are present in Fig 2a. The data are processed starting with SR90. Differential analysis was performed between the C2Y1E and C1Y2E sample groups. Differential proteins are determined with p-value < 0.05 and $|\log_2 FC| > \log_2 1.5$.
