# Supplementary material for: Benchmarking informatics workflows for data-independent acquisition single-cell proteomics
Source: Nat Commun. 2025 Nov 21;16:10276. doi: 10.1038/s41467-025-65174-4 (PMC12639053; doi:10.1038/s41467-025-65174-4)
Supplement: Supplementary file 9 — Supplementary Data 7 [file 41467_2025_65174_MOESM9_ESM.zip › FigSD7-[1-12] GO Enrichment.pdf]

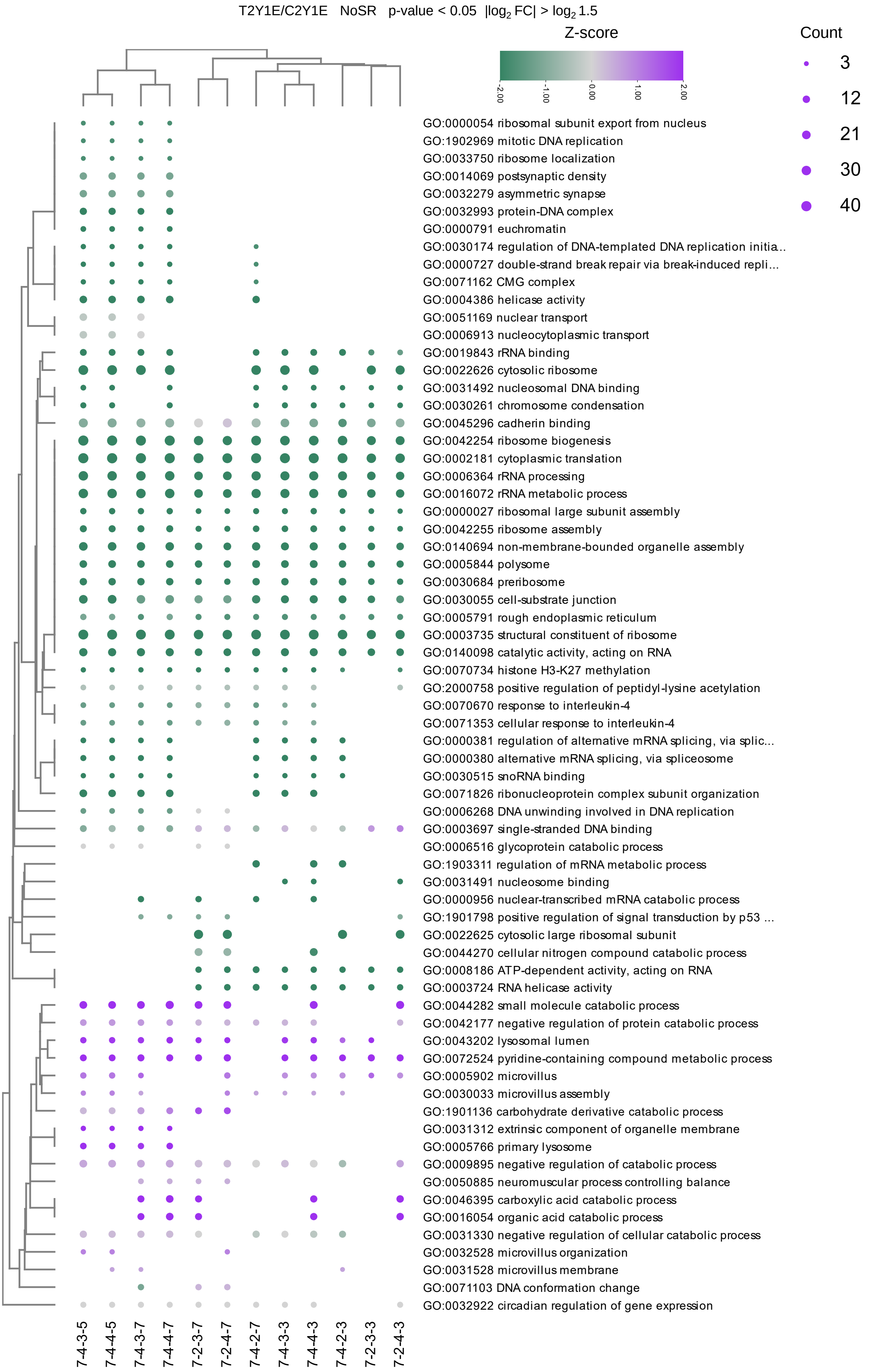

(Legend on next page)

Figure SD7-1. Comparison of GO enrichment results from the differential proteins found by high-performing method combinations (DIA-NN NoSR)

Colors indicate Z-scores of terms. Dot sizes indicate the number of differential proteins. Rows and columns were clustered with Jaccard distances. Mappings of the serial numbers to detailed methods for each step are present in Fig 2a. The data are processed starting with NoSR. Differential analysis was performed between the T2Y1E and C2Y1E sample groups. Differential proteins are determined with p-value < 0.05 and $|\log_2 FC| > \log_2 1.5$.

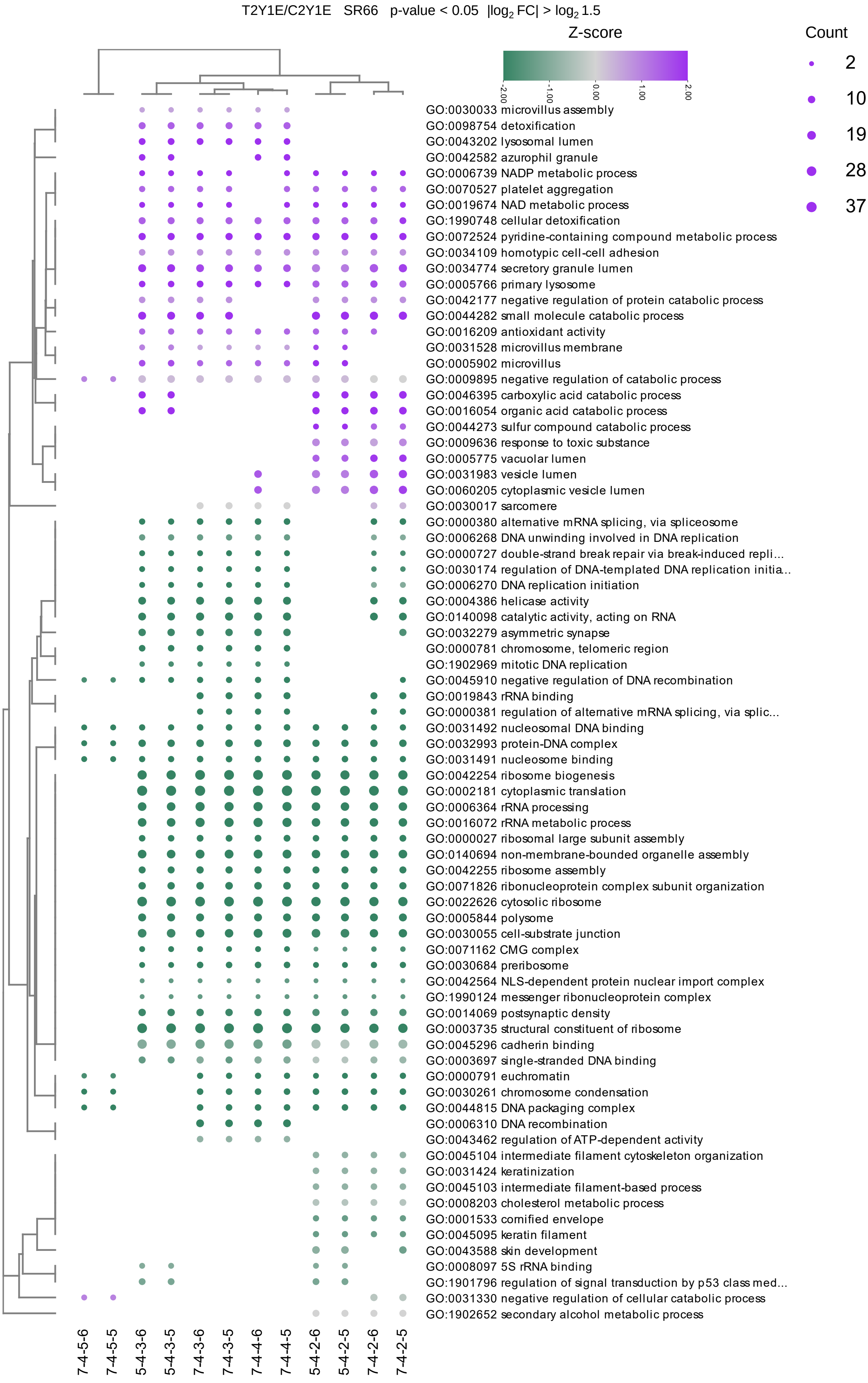

(Legend on next page)

Figure SD7-2. Comparison of GO enrichment results from the differential proteins found by high-performing method combinations (DIA-NN SR66)

Colors indicate Z-scores of terms. Dot sizes indicate the number of differential proteins. Rows and columns were clustered with Jaccard distances. Mappings of the serial numbers to detailed methods for each step are present in Fig 2a. The data are processed starting with SR66. Differential analysis was performed between the T2Y1E and C2Y1E sample groups. Differential proteins are determined with p-value < 0.05 and $|\log_2 FC| > \log_2 1.5$.

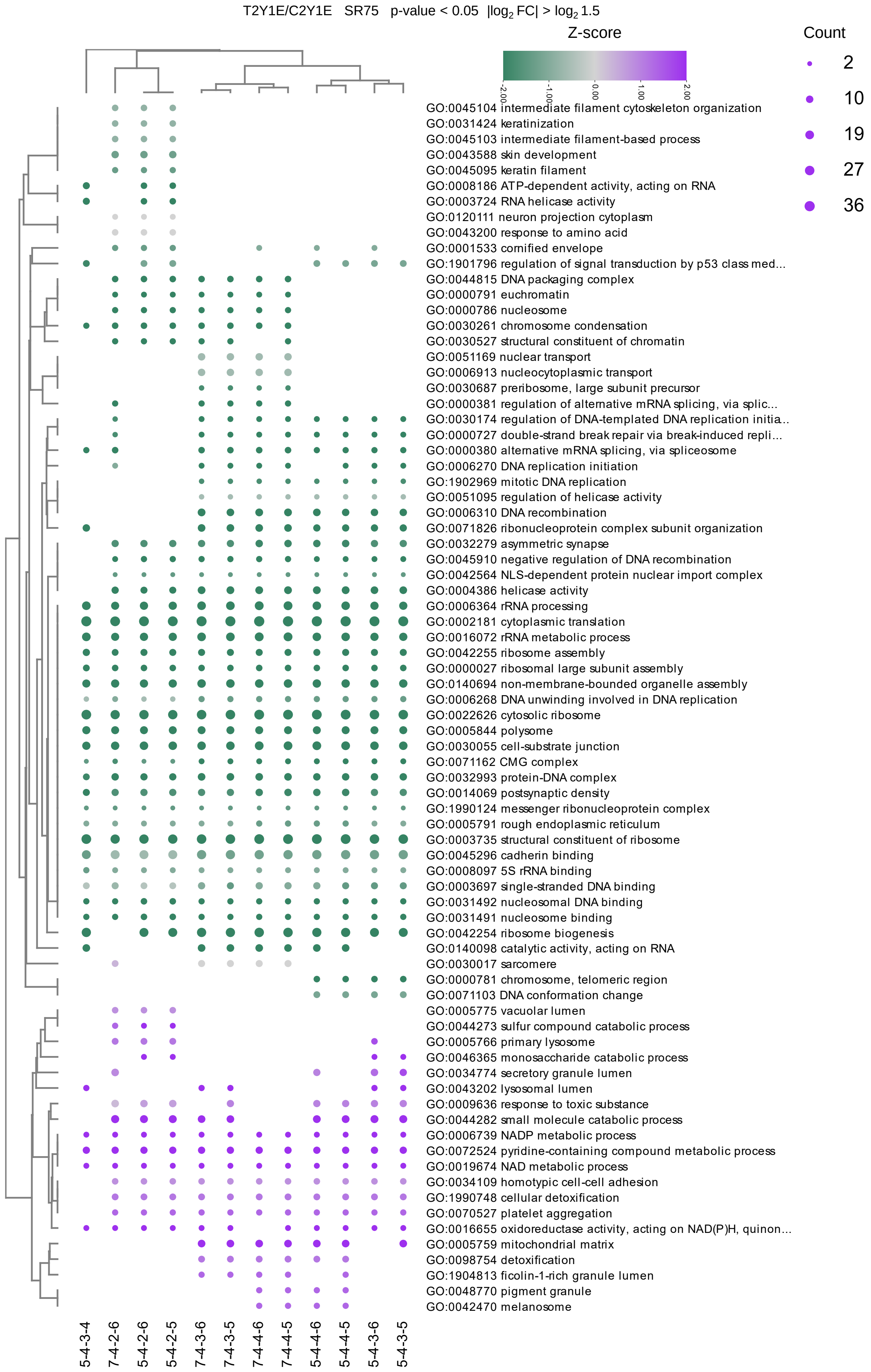

(Legend on next page)

Figure SD7-3. Comparison of GO enrichment results from the differential proteins found by high-performing method combinations (DIA-NN SR75)

Colors indicate Z-scores of terms. Dot sizes indicate the number of differential proteins. Rows and columns were clustered with Jaccard distances. Mappings of the serial numbers to detailed methods for each step are present in Fig 2a. The data are processed starting with SR75. Differential analysis was performed between the T2Y1E and C2Y1E sample groups. Differential proteins are determined with p-value < 0.05 and $|\log_2 FC| > \log_2 1.5$.

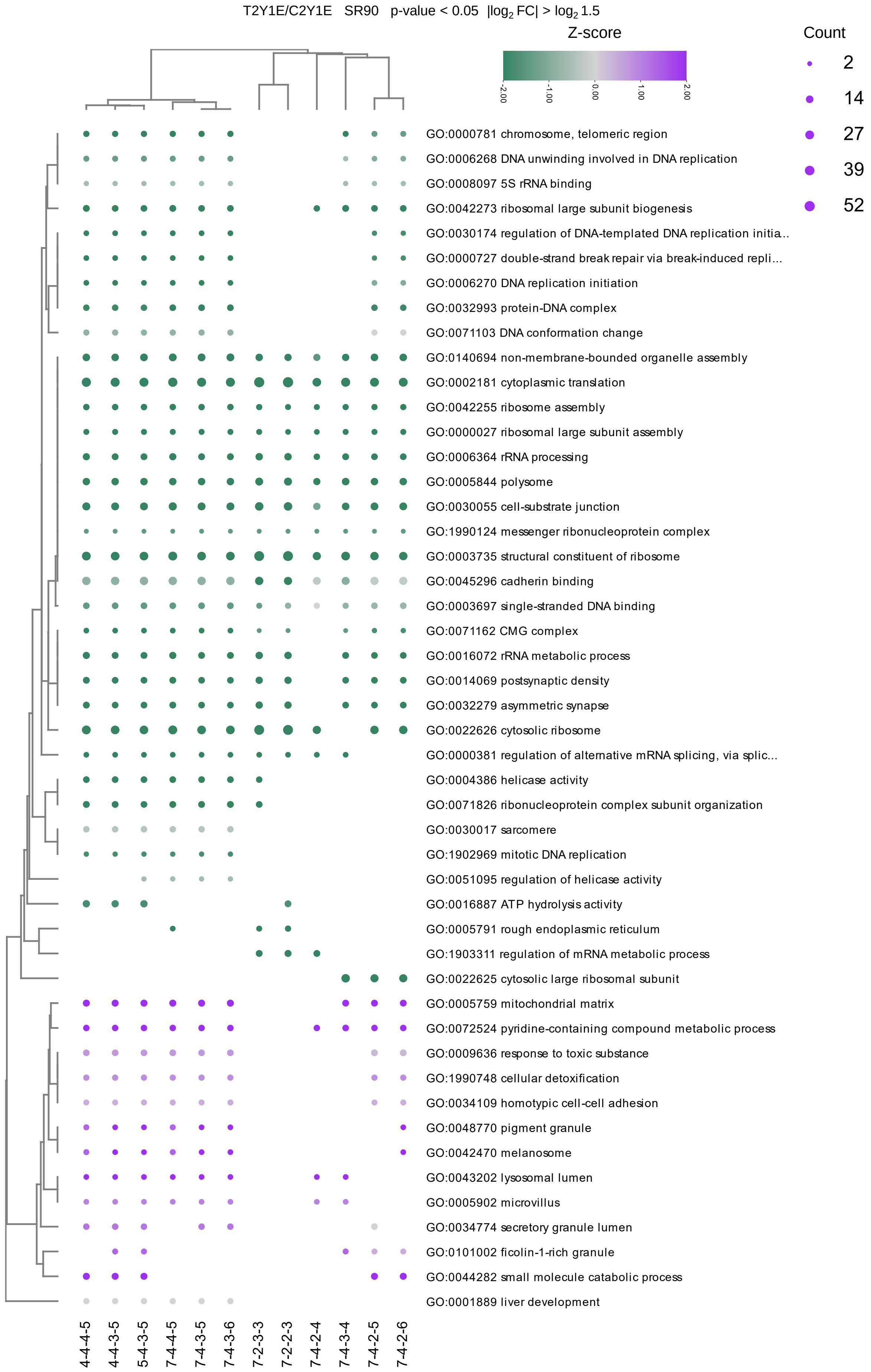

(Legend on next page)

Figure SD7-4. Comparison of GO enrichment results from the differential proteins found by high-performing method combinations (DIA-NN SR90)

Colors indicate Z-scores of terms. Dot sizes indicate the number of differential proteins. Rows and columns were clustered with Jaccard distances. Mappings of the serial numbers to detailed methods for each step are present in Fig 2a. The data are processed starting with SR90. Differential analysis was performed between the T2Y1E and C2Y1E sample groups. Differential proteins are determined with p-value < 0.05 and $|\log_2 FC| > \log_2 1.5$.

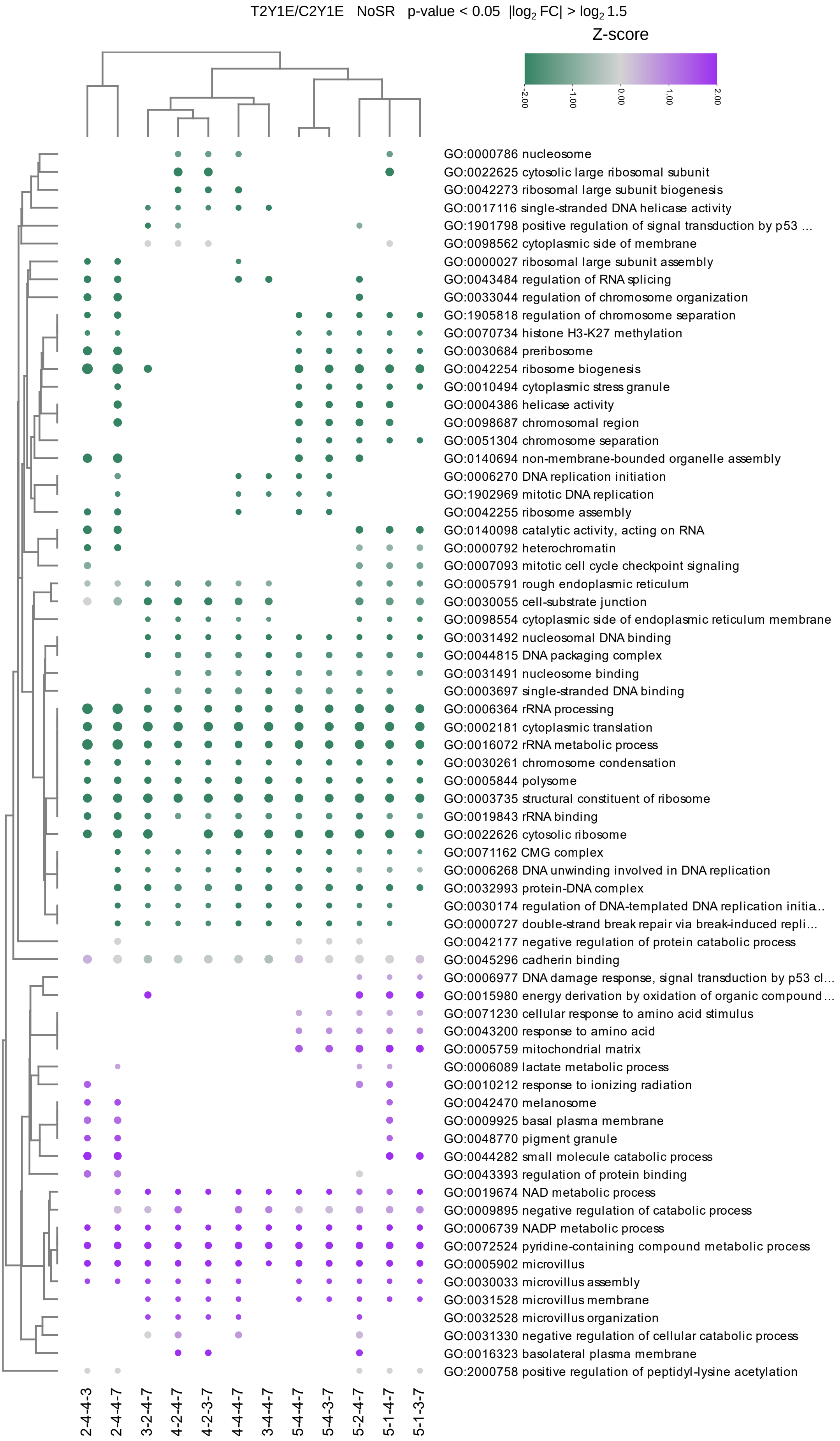

(Legend on next page)

Figure SD7-5. Comparison of GO enrichment results from the differential proteins found by high-performing method combinations (Spectronaut NoSR)

Colors indicate Z-scores of terms. Dot sizes indicate the number of differential proteins. Rows and columns were clustered with Jaccard distances. Mappings of the serial numbers to detailed methods for each step are present in Fig 2a. The data are processed starting with NoSR. Differential analysis was performed between the T2Y1E and C2Y1E sample groups. Differential proteins are determined with p-value < 0.05 and $|\log_2 FC| > \log_2 1.5$.

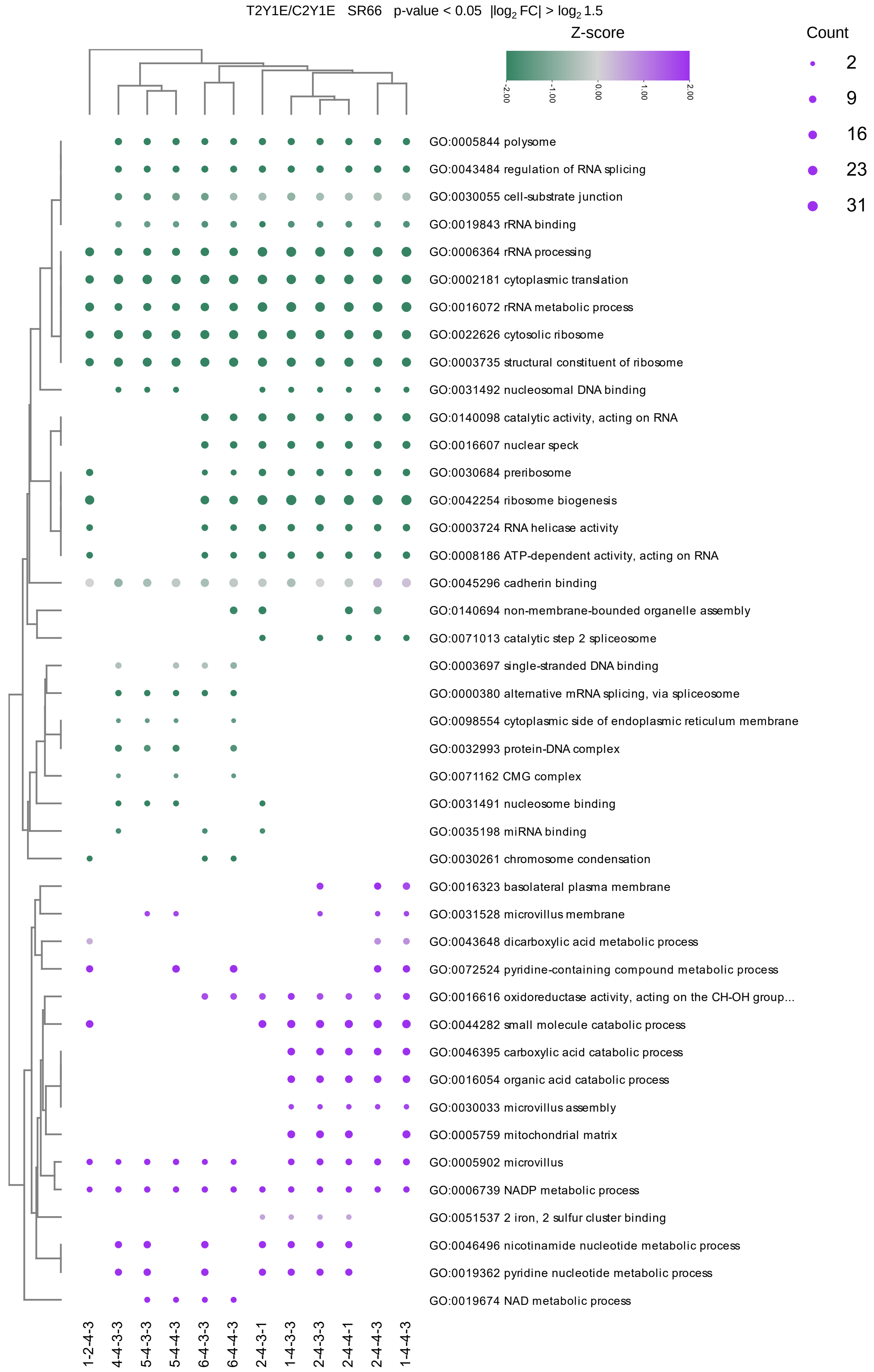

(Legend on next page)

Figure SD7-6. Comparison of GO enrichment results from the differential proteins found by high-performing method combinations (Spectronaut SR66)

Colors indicate Z-scores of terms. Dot sizes indicate the number of differential proteins. Rows and columns were clustered with Jaccard distances. Mappings of the serial numbers to detailed methods for each step are present in Fig 2a. The data are processed starting with SR66. Differential analysis was performed between the T2Y1E and C2Y1E sample groups. Differential proteins are determined with p-value < 0.05 and $|\log_2 FC| > \log_2 1.5$.

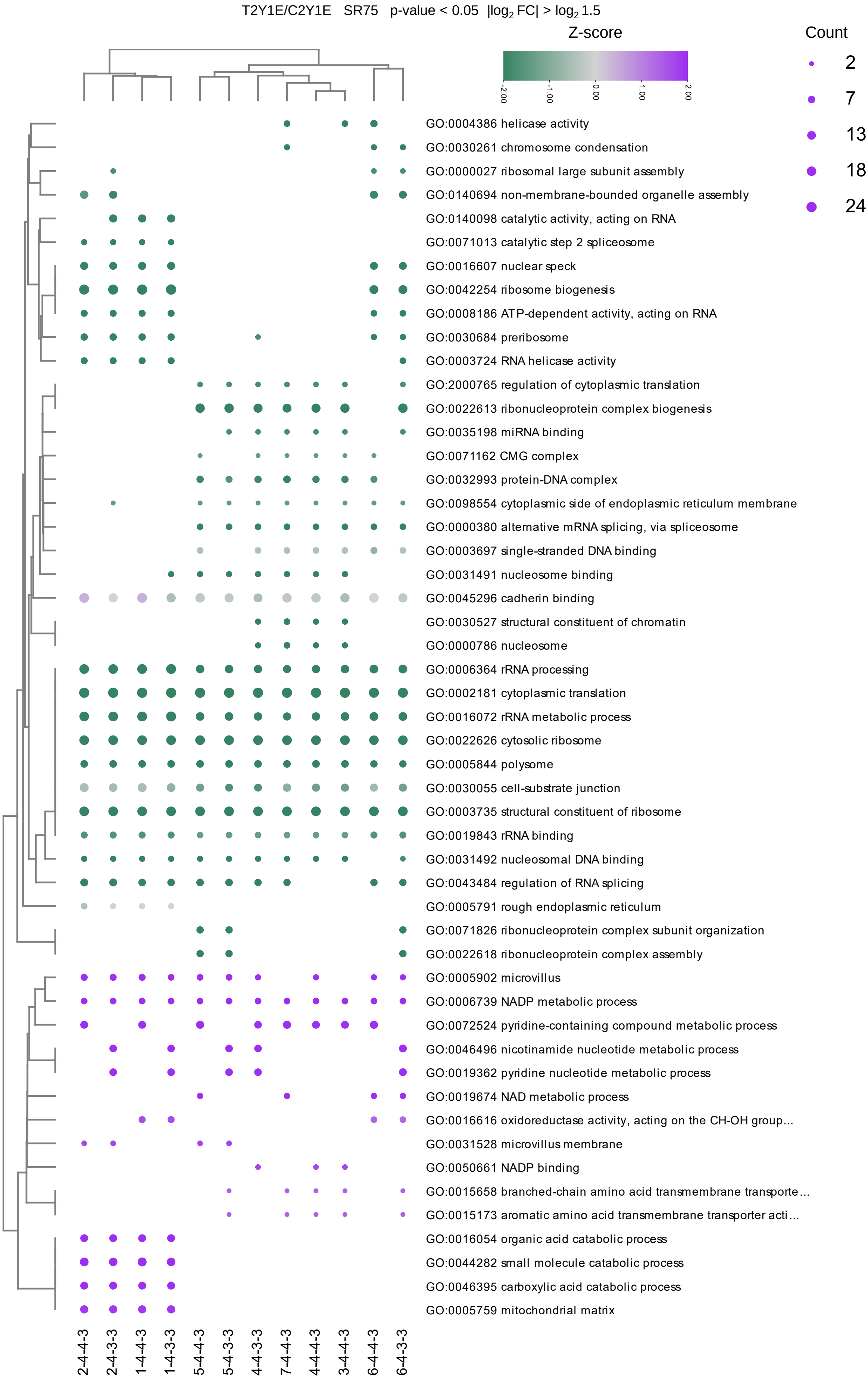

(Legend on next page)

Figure SD7-7. Comparison of GO enrichment results from the differential proteins found by high-performing method combinations (Spectronaut SR75)

Colors indicate Z-scores of terms. Dot sizes indicate the number of differential proteins. Rows and columns were clustered with Jaccard distances. Mappings of the serial numbers to detailed methods for each step are present in Fig 2a. The data are processed starting with SR75. Differential analysis was performed between the T2Y1E and C2Y1E sample groups. Differential proteins are determined with $p\text{-value} < 0.05$ and $|\log_2 FC| > \log_2 1.5$.

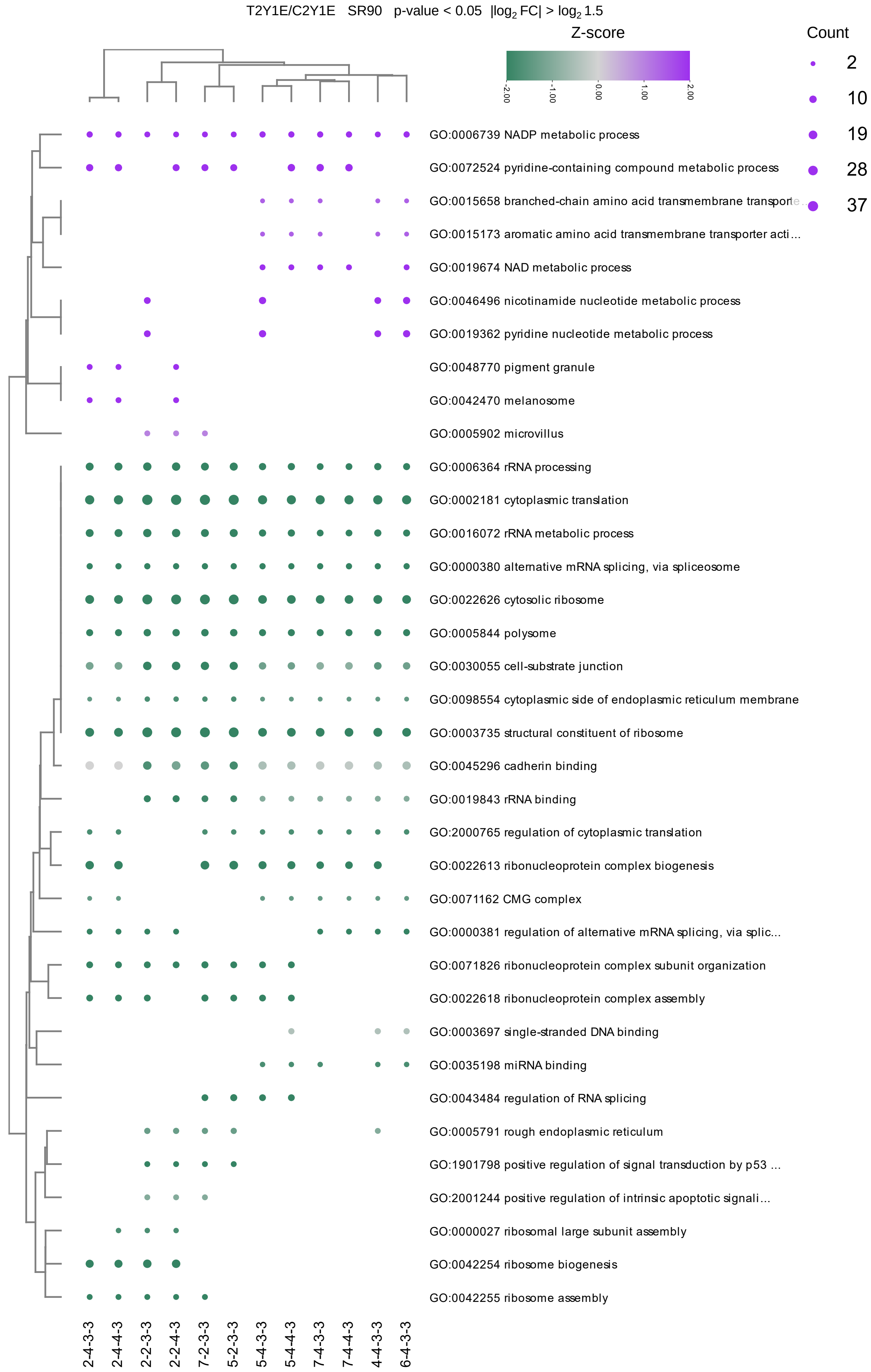

(Legend on next page)

Figure SD7-8. Comparison of GO enrichment results from the differential proteins found by high-performing method combinations (Spectronaut SR90)

Colors indicate Z-scores of terms. Dot sizes indicate the number of differential proteins. Rows and columns were clustered with Jaccard distances. Mappings of the serial numbers to detailed methods for each step are present in Fig 2a. The data are processed starting with SR90. Differential analysis was performed between the T2Y1E and C2Y1E sample groups. Differential proteins are determined with $p\text{-value} < 0.05$ and $|\log_2 FC| > \log_2 1.5$.

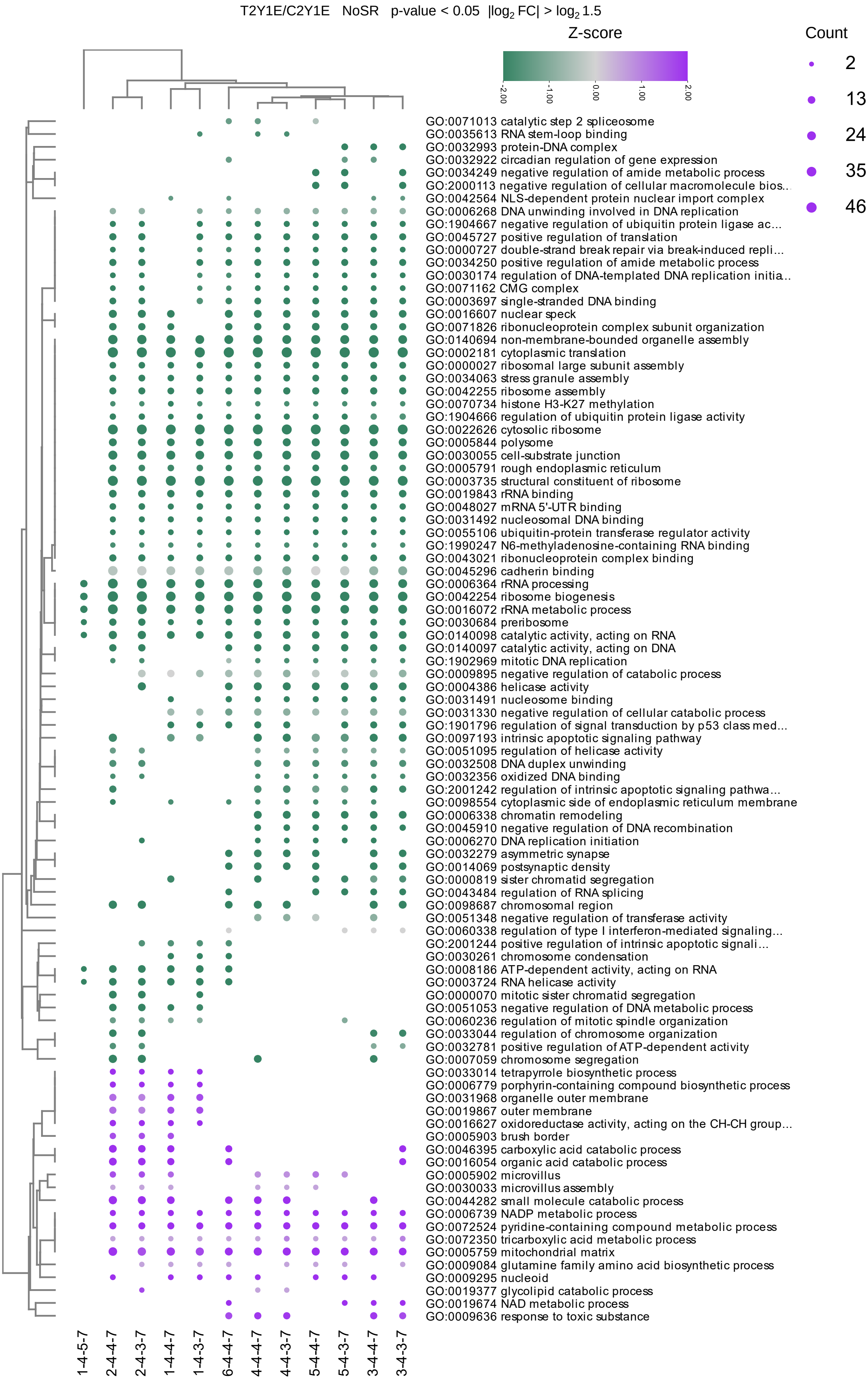

(Legend on next page)

Figure SD7-9. Comparison of GO enrichment results from the differential proteins found by high-performing method combinations (PEAKS NoSR)

Colors indicate Z-scores of terms. Dot sizes indicate the number of differential proteins. Rows and columns were clustered with Jaccard distances. Mappings of the serial numbers to detailed methods for each step are present in Fig 2a. The data are processed starting with NoSR. Differential analysis was performed between the T2Y1E and C2Y1E sample groups. Differential proteins are determined with p-value < 0.05 and $|\log_2 FC| > \log_2 1.5$.

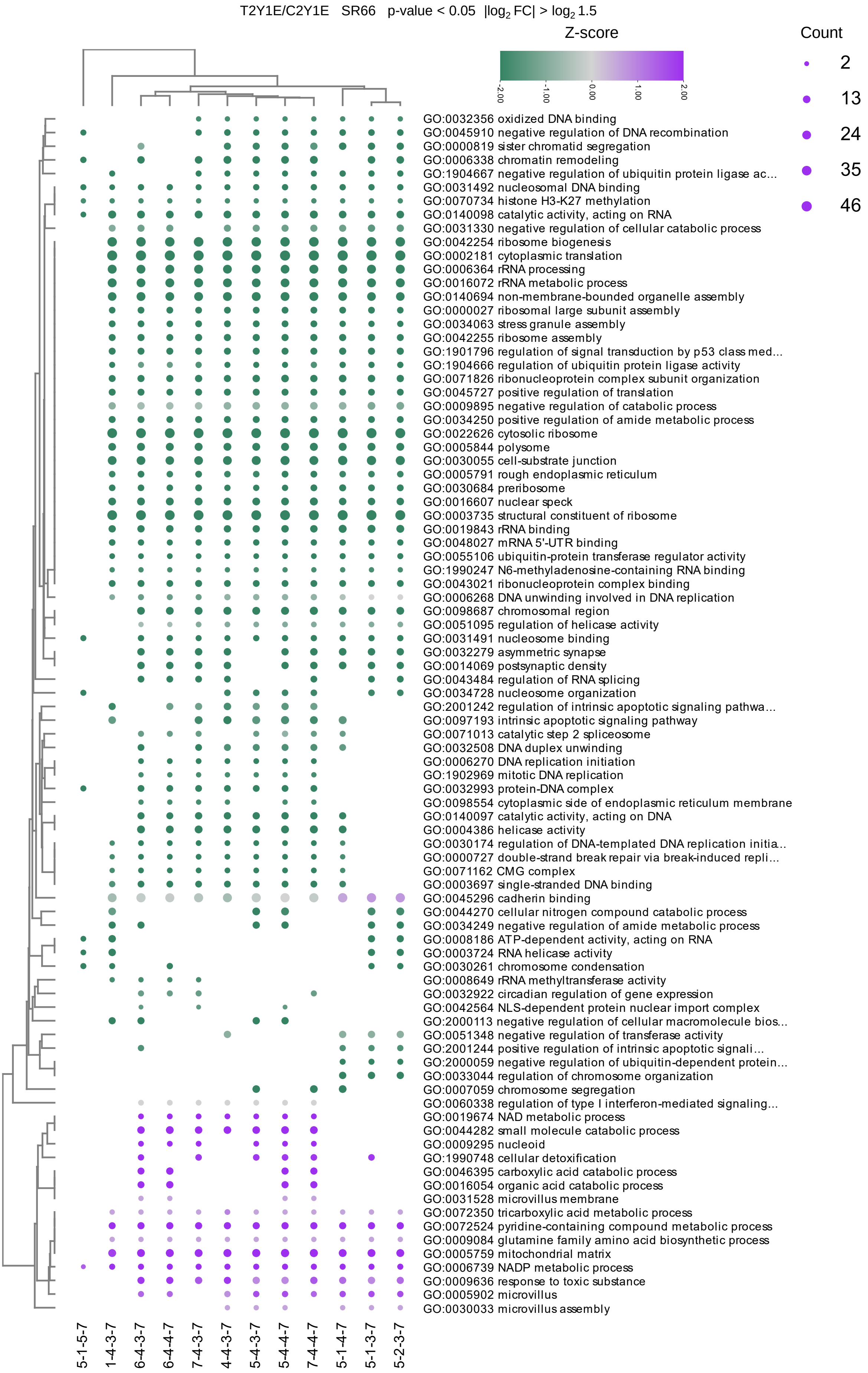

(Legend on next page)

Figure SD7-10. Comparison of GO enrichment results from the differential proteins found by high-performing method combinations (PEAKS SR66)

Colors indicate Z-scores of terms. Dot sizes indicate the number of differential proteins. Rows and columns were clustered with Jaccard distances. Mappings of the serial numbers to detailed methods for each step are present in Fig 2a. The data are processed starting with SR66. Differential analysis was performed between the T2Y1E and C2Y1E sample groups. Differential proteins are determined with p-value < 0.05 and $|\log_2 FC| > \log_2 1.5$.

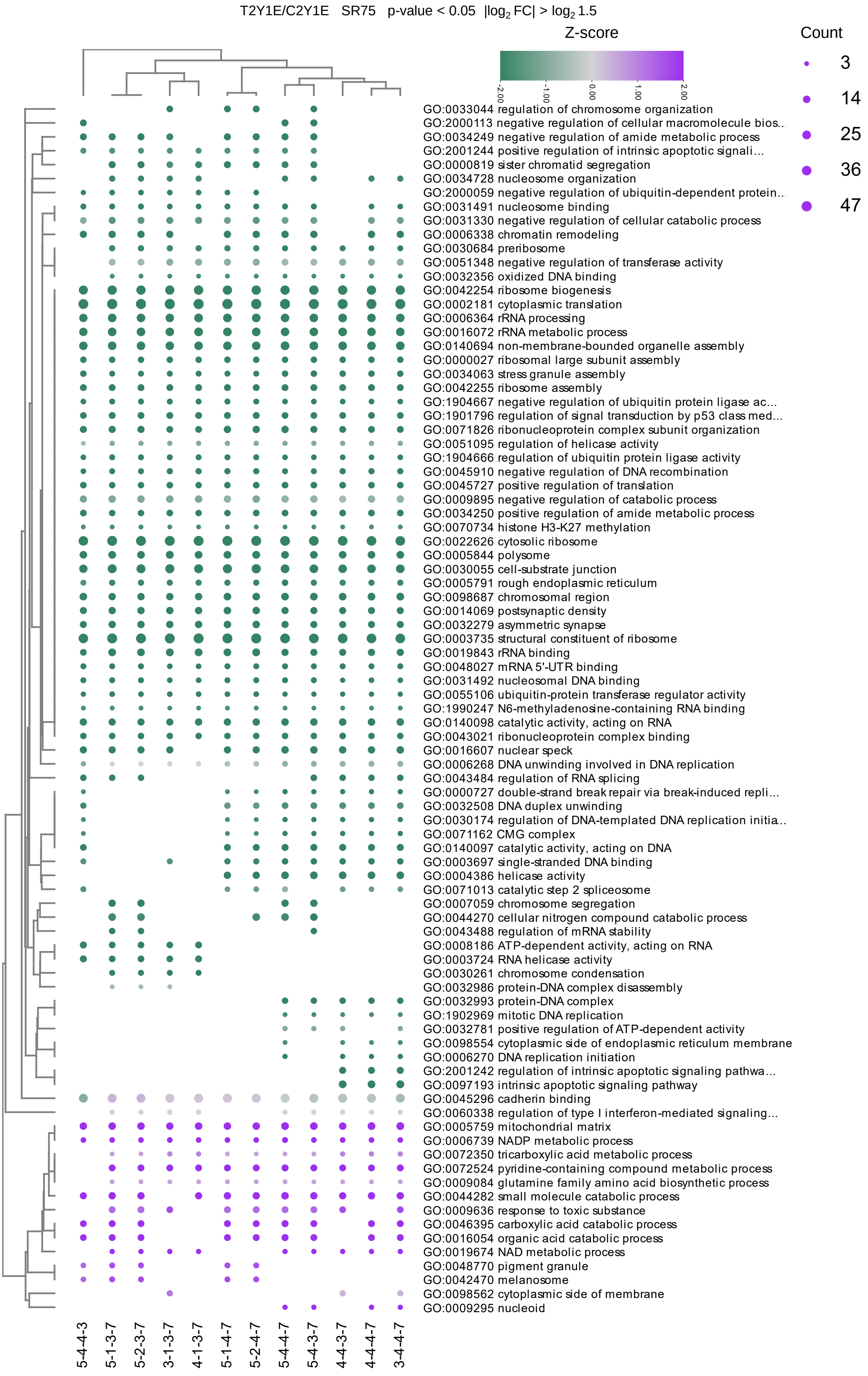

(Legend on next page)

Figure SD7-11. Comparison of GO enrichment results from the differential proteins found by high-performing method combinations (PEAKS SR75)

Colors indicate Z-scores of terms. Dot sizes indicate the number of differential proteins. Rows and columns were clustered with Jaccard distances. Mappings of the serial numbers to detailed methods for each step are present in Fig 2a. The data are processed starting with SR75. Differential analysis was performed between the T2Y1E and C2Y1E sample groups. Differential proteins are determined with p-value < 0.05 and $|\log_2 FC| > \log_2 1.5$.

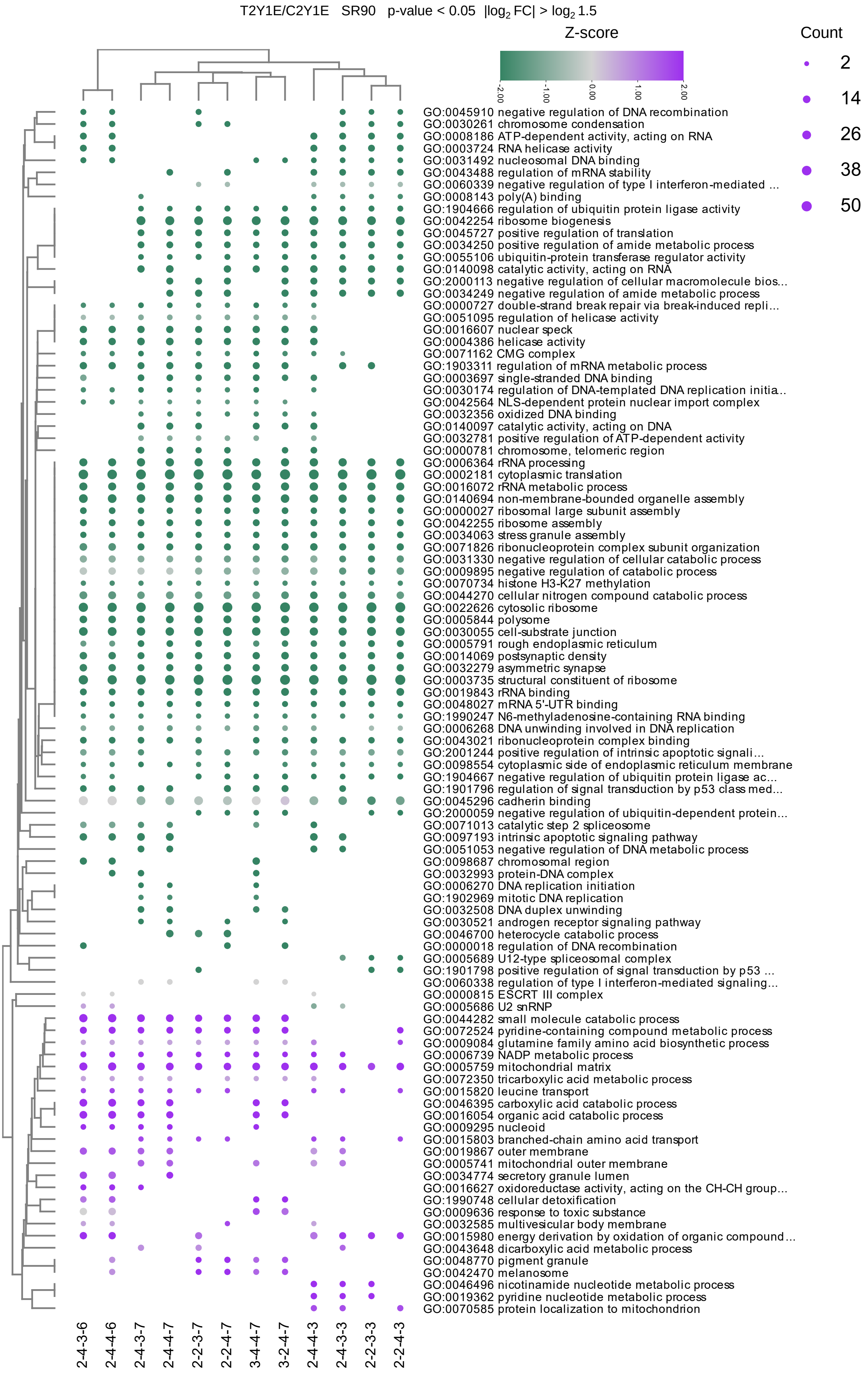

(Legend on next page)

Figure SD7-12. Comparison of GO enrichment results from the differential proteins found by high-performing method combinations (PEAKS SR90)

Colors indicate Z-scores of terms. Dot sizes indicate the number of differential proteins. Rows and columns were clustered with Jaccard distances. Mappings of the serial numbers to detailed methods for each step are present in Fig 2a. The data are processed starting with SR90. Differential analysis was performed between the T2Y1E and C2Y1E sample groups. Differential proteins are determined with p-value < 0.05 and $|\log_2 FC| > \log_2 1.5$.
