# Supplementary material for: Benchmarking informatics workflows for data-independent acquisition single-cell proteomics
Source: Nat Commun. 2025 Nov 21;16:10276. doi: 10.1038/s41467-025-65174-4 (PMC12639053; doi:10.1038/s41467-025-65174-4)
Supplement: Supplementary file 10 — Reporting Summary [file 41467_2025_65174_MOESM10_ESM.pdf]

Corresponding author(s): Yi Yang, Qun Fang

Last updated by author(s): Sep 17, 2025

Reporting Summary

Nature Portfolio wishes to improve the reproducibility of the work that we publish. This form provides structure for consistency and transparency in reporting. For further information on Nature Portfolio policies, see our Editorial Policies and the Editorial Policy Checklist.

Statistics

For all statistical analyses, confirm that the following items are present in the figure legend, table legend, main text, or Methods section.

n/a	Confirmed	
☐	☒	The exact sample size (*n*) for each experimental group/condition, given as a discrete number and unit of measurement
☐	☒	A statement on whether measurements were taken from distinct samples or whether the same sample was measured repeatedly
☐	☒	The statistical test(s) used AND whether they are one- or two-sided<br>*Only common tests should be described solely by name; describe more complex techniques in the Methods section.*
☒	☐	A description of all covariates tested
☐	☒	A description of any assumptions or corrections, such as tests of normality and adjustment for multiple comparisons
☐	☒	A full description of the statistical parameters including central tendency (e.g. means) or other basic estimates (e.g. regression coefficient) AND variation (e.g. standard deviation) or associated estimates of uncertainty (e.g. confidence intervals)
☐	☒	For null hypothesis testing, the test statistic (e.g. *F*, *t*, *r*) with confidence intervals, effect sizes, degrees of freedom and *P* value noted<br>*Give P values as exact values whenever suitable.*
☒	☐	For Bayesian analysis, information on the choice of priors and Markov chain Monte Carlo settings
☒	☐	For hierarchical and complex designs, identification of the appropriate level for tests and full reporting of outcomes
☐	☒	Estimates of effect sizes (e.g. Cohen's *d*, Pearson's *r*), indicating how they were calculated

Our web collection on statistics for biologists contains articles on many of the points above.

Software and code

Policy information about availability of computer code

Data collection	Mass spectrometry data was collected using Compass HyStar software (version 6.0 and 5.1).
Data analysis	Raw DIA data were analyzed by DIA-NN (version 1.9.2), Spectronaut (version 9.5.241126.62635, Biognosys), and PEAKS Studio (version 12.0, Bioinformatics Solutions). In addition,FragPipe (version 22.0) with MSFragger (version 4.1), IonQuant (version 1.10.27), and Philosopher (version 5.1.1) were used to build experimental spectral libraries. AlphaPeptDeep (version 1.0.2) was used to generate predicted spectral libraries.<br>Bioinformatics analysis was conducted with the Python packages fancyimpute (version 0.7.0), scanorama (version 1.7.4), scipy (version 1.5.4), and scikit-learn (version 0.24.2), as well as the R packages limma (version 3.56.2), MBQN (version 2.12.0), sva (version 3.48.0), edgeR (version 3.42.4), DESeq2 (version 1.40.2), Seurat (version 4.3.0.1), clusterprofiler (version 4.12.6), and ReactomePA (version 1.48.0). Machine learning and explanation were performed with the Python packages xgboost (version 1.5.2) and shap (version 0.41.0). Visualization of the tree was implemented by Graphviz Online (https://dreampuf.github.io/GraphvizOnline/).<br>Custom code is available at Github (https://github.com/WangJianwei1991/SCPDA) and Zenodo (https://zenodo.org/records/17140070).

For manuscripts utilizing custom algorithms or software that are central to the research but not yet described in published literature, software must be made available to editors and reviewers. We strongly encourage code deposition in a community repository (e.g. GitHub). See the Nature Portfolio guidelines for submitting code & software for further information.

Data

Policy information about availability of data

All manuscripts must include a data availability statement. This statement should provide the following information, where applicable:

- Accession codes, unique identifiers, or web links for publicly available datasets
- A description of any restrictions on data availability
- For clinical datasets or third party data, please ensure that the statement adheres to our policy

Raw mass spectrometry data, spectral libraries, and search results have been deposited in the ProteomeXchange Consortium via the iProX partner repository with the dataset identifier PXD056832 [https://proteomecentral.proteomexchange.org/cgi/GetDataset?ID=PXD056832] or IPX0009767000 [https://www.iprox.cn/page/project.html?id=IPX0009767000]. Source data are provided with this paper.

Research involving human participants, their data, or biological material

Policy information about studies with human participants or human data. See also policy information about sex, gender (identity/presentation), and sexual orientation and race, ethnicity and racism.

Reporting on sex and gender	No human participants involved.
Reporting on race, ethnicity, or other socially relevant groupings	No human participants involved.
Population characteristics	No human participants involved.
Recruitment	No human participants involved.
Ethics oversight	No human participants involved.

Note that full information on the approval of the study protocol must also be provided in the manuscript.

Field-specific reporting

Please select the one below that is the best fit for your research. If you are not sure, read the appropriate sections before making your selection.

☒ Life sciences ☐ Behavioural & social sciences ☐ Ecological, evolutionary & environmental sciences

For a reference copy of the document with all sections, see nature.com/documents/nr-reporting-summary-flat.pdf

Life sciences study design

All studies must disclose on these points even when the disclosure is negative.

Sample size	We did not perform sample size calculations. The sample size was chosen based on previous benchmarking studies [doi:10.1038/s41467-022-30094-0, doi:10.1016/j.mcpro.2023.100623, doi:10.1038/s41467-022-35740-1] and is consistent with established practices in similar research.
Data exclusions	Samples of failed cell picking-up or preparation were excluded based on very low protein identification numbers.
Replication	For all samples included in this study multiple replicates are shown, indicated with the respective sample size. The benchmarking was performed on multiple datasets to verify the reproducibility of the findings. All attempts at replication were successful.
Randomization	The order of LC-MS/MS runs were alternated between the biological replicates.
Blinding	The investigators were not blinded to the sample groups. The same set of computational pipelines were carried out for data analysis, independent on samples.

Reporting for specific materials, systems and methods

We require information from authors about some types of materials, experimental systems and methods used in many studies. Here, indicate whether each material, system or method listed is relevant to your study. If you are not sure if a list item applies to your research, read the appropriate section before selecting a response.

Materials & experimental systems

n/a	Involved in the study
☒	☐ Antibodies
☐	☒ Eukaryotic cell lines
☒	☐ Palaeontology and archaeology
☒	☐ Animals and other organisms
☒	☐ Clinical data
☒	☐ Dual use research of concern
☒	☐ Plants

Methods

n/a	Involved in the study
☒	☐ ChIP-seq
☒	☐ Flow cytometry
☒	☐ MRI-based neuroimaging

Eukaryotic cell lines

Policy information about cell lines and Sex and Gender in Research

Cell line source(s)	Human breast adenocarcinoma MCF-7 cells (HTB-22) were from American Type Culture Collection.
Authentication	Cells were authenticated by STR profiling.
Mycoplasma contamination	Cells were tested mycoplasma negative
Commonly misidentified lines (See ICLAC register)	No commonly misidentified lines.

Plants

Seed stocks	No plants involved.
Novel plant genotypes	No plants involved.
Authentication	No plants involved.
